# Desert plants, arbuscular mycorrhizal fungi and associated bacteria: Exploring the diversity and role of symbiosis under drought

**DOI:** 10.1111/1758-2229.13300

**Published:** 2024-07-09

**Authors:** Jose Daniel Chávez‐González, Víctor M. Flores‐Núñez, Irving U. Merino‐Espinoza, Laila Pamela Partida‐Martínez

**Affiliations:** ^1^ Departamento de Ingeniería Genética, Centro de Investigación y de Estudios Avanzados del Instituto Politécnico Nacional (Cinvestav) Irapuato Mexico; ^2^ Present address: Christian‐Albrechts University of Kiel Kiel Germany

## Abstract

Desert plants, such as *Agave tequilana*, *A. salmiana* and *Myrtillocactus geometrizans*, can survive harsh environmental conditions partly due to their symbiotic relationships with microorganisms, including arbuscular mycorrhizal fungi (AMF). Interestingly, some of these fungi also harbour endosymbiotic bacteria. Our research focused on investigating the diversity of these AMFs and their associated bacteria in these plants growing in arid soil. We found that agaves have a threefold higher AMF colonization than *M. geometrizans*. Metabarcoding techniques revealed that the composition of AMF communities was primarily influenced by the plant host, while the bacterial communities were more affected by the specific plant compartment or niche they inhabited. We identified both known and novel endofungal bacterial taxa, including Burkholderiales, and confirmed their presence within AMF spores using multiphoton microscopy. Our study also explored the effects of drought on the symbiosis between *A. tequilana* and AMF. We discovered that the severity of drought conditions could modulate the strength of this symbiosis and its outcomes for the plant holobiont. Severe drought conditions prevented the formation of this symbiosis, while moderate drought conditions promoted it, thereby conferring drought tolerance in *A. tequilana*. This research sheds light on the diversity of AMF and associated bacteria in Crassulacean Acid Metabolism (CAM) plants and underscores the crucial role of drought as a factor modulating the symbiosis between *A. tequilana* and AMF. Further research is needed to understand the role of endofungal bacteria in this response.

## Graphical abstract

Arbuscular mycorrhizal fungi, along with their endobacteria, could potentially aid desert plants, specifically those using Crassulacean Acid Metabolism (CAM), in surviving drought conditions prevalent in arid ecosystems. This research highlights the diversity and presence of these three‐way symbiotic relationships in agave and cactus plants. Furthermore, it underscores the significant role of drought as a factor that influences the symbiotic relationship between the plant and fungi, and the subsequent effects on the overall plant organism, or ‘holobiont’.

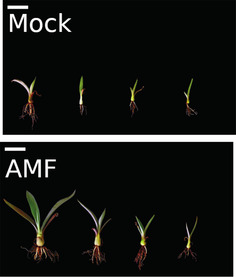

## INTRODUCTION

Arbuscular mycorrhizal fungi (AMF) belong to the monophyletic subphylum Glomeromycotina (Spatafora et al., 2016) and form the oldest known symbiotic associations with the roots of about 72%–79% of terrestrial plants, including essential crops (Genre et al., 2020; Tedersoo et al., 2020).

Studies focused on the interactions of AMF with microorganisms revealed, since 1970 (Mosse, 1970), that some AMF harbour morphologically diverse lineages of endofungal bacteria (Agnolucci et al., 2019; Araldi‐Brondolo et al., 2017; Bianciotto et al., 1996, 2000; Bonfante & Anca, 2009; Lumini et al., 2007; Scannerini & Bonfante, 1991; Uehling et al., 2023). Most of the endofungal bacteria hitherto reported in Glomeromycotina have been classified into two main lineages: BRE for *Burkholderia*‐related endobacteria and MRE for *Mycoplasma*‐related endobacteria (Bianciotto et al., 2003; Naito et al., 2017; Naumann et al., 2010) being MRE most diverse and frequent in AMF than BRE (Lastovetsky et al., 2018, 2024).

Lumini et al. (2007) gave the first report on the influence of endobacteria on AMF physiology. These authors described a yet unculturable and obligate BRE known as *Candidatus* Glomeribacter gigasporarum (*Ca*Gg) hosted by *Gigaspora margarita* (*Gm*). They generated monosporic *Gm* cultures and obtained spores that lacked *Ca*Gg. These cured spores showed changes in vacuole morphology, cell wall organization, lipid bodies and pigment granules. Importantly, the lack of *Ca*Gg severely affected pre‐symbiotic fungal growth, such as hyphal elongation and branching after treatment with root exudates (Lumini et al., 2007). Studies using ‘omics’ approaches on these *Gm* (B+) and *Gm* (B−) lines have revealed that *Ca*Gg enhances fungal fitness by priming mitochondrial and antioxidant metabolism (Salvioli et al., 2016; Vannini et al., 2016). In addition, a study focused on the interaction of *Gm*‐*Ca*Gg and tomato plants under combined drought and nutrient stress revealed that in this system, tomato mycorrhization was low with both B+ and B− lines, and no significant effects on plant growth or tolerance to stress were noticeable. However, plant genes that sense AMF were turned on (Chialva et al., 2020).

MRE from Glomeromycotina encompasses the novel species *Candidatus* Moeniiplasma glomeromycotorum (*Ca*Mg) (Naito et al., 2017). The impact of this group of endobacteria in AMF biology remains a tantalizing mystery, as AMF spores devoid of these MRE symbionts have yet to be obtained, and efforts to cultivate *Ca*Mg outside their fungal hosts have been unsuccessful to date. However, it has been hypothesized that *Ca*Mg may be non‐lethal parasites of Glomeromycotina and Mortierellomycotina fungi (Desiro et al., 2018; Naito et al., 2017) or conditional mutualists of their AMF hosts (Lastovetsky et al., 2024).

Besides BRE and MRE, some reports have suggested that AMF could harbour a greater diversity of bacterial endosymbionts, but to date, evidence of their role in the plant‐fungal symbiosis does not exist (Faghihinia et al., 2023; Lastovetsky et al., 2024; Pandit et al., 2022; Wang et al., 2022).

Arid zones represent 44% of the world's surface area and crops in these ecosystems are exposed to high temperatures, high solar radiation and drought (Gaur & Squires, 2018). Drought is the actual major abiotic stress‐reducing plant survival, growth and productivity (Oguz et al., 2022; Seleiman et al., 2021). However, desert plants have developed different strategies to tolerate drought events common in drylands (Bahadur et al., 2019). One of these strategies is the establishment of symbioses with microorganisms.

Our pioneer work on five keystone species of agaves and cacti in arid and semi‐arid ecosystems in the American continent shed light on the prokaryotic and fungal communities associated with the soils, rhizosphere, roots, leaves and phyllosphere of these photosynthetic efficient Crassulacean Acid Metabolism (CAM) plants (Citlali et al., 2018; Coleman‐Derr et al., 2016; Fonseca‐Garcia et al., 2016). From these molecular studies using the ITS2 marker, we learned that only 48 out of nearly 4000 fungal OTUs were classified as AMF, representing on average 1.1% of the relative abundance in the below‐ground fungal communities associated with wild agaves and cacti. Samples from the cultivated *A. tequilana* were nearly devoid of AMF taxa in all niches investigated, including the soils. We also identified that AMF richness is inversely correlated with the availability of phosphorus in the soil, being highest in San Felipe, Guanajuato, Mexico (Figure S1, Tables S1–S3).

Other ecological studies on agaves and cacti have quantified notable benefits on plant development, water and nutrient uptake, and biomass when plants are grown with AMF (Cui & Nobel, 1992; Hernandez‐Cuevas et al., 2023; Lahbouki, Anli, et al., 2022; Lahbouki, Ben‐Laouane, et al., 2022; Montoya‐Martínez et al., 2019; Pimienta‐Barrios et al., 2003; Quiñones‐Aguilar et al., 2016). Nevertheless, the occurrence and diversity of endofungal bacteria associated with native arbuscular mycorrhizal fungi in CAM plants have never been investigated. Also, the impact of AMF on the tolerance to drought in *A. tequilana* has not been addressed. We investigated the diversity and prevalence of bacteria associated with AMF in the desert CAM plants *A. tequilana*, *A. salmiana* and the cactus *M. geometrizans*. Furthermore, we evaluated the role played by AMF natives of arid ecosystems in the responses of *A. tequilana* plants to drought under greenhouse conditions.

## EXPERIMENTAL PROCEDURES

### Soil and AMF sampling

We collected bulk soil and root‐zone soil associated with the wild and native plants of *A. salmiana*, *M. geometrizans* and *O. robusta* growing in San Felipe (SF), Guanajuato, Mexico (Figure S1c). We chose this site since it harboured the highest richness of AMF across seven arid and semi‐arid sites in the United States and Mexico (Figure S1) (Citlali et al., 2018; Coleman‐Derr et al., 2016; Fonseca‐Garcia et al., 2016). We mixed all the recovered soil and used it to grow 15 micropropagated plants each of *A. tequilana*, *A. salmiana* and *M. geometrizans* for 12 months under greenhouse conditions. For this experiment, only one irrigation per week was conducted to maintain the substrate at field capacity (Figure S2). We randomly sampled four plants of each species every 4 months (at T4, T8 and T12) to determine the colonization of the roots and the amount of spores produced. We employed the clearing and staining technique with Trypan blue (0.05%) as described by Phillips and Hayman (1970) and we quantified the percentage of mycorrhizal colonization as described by McGonigle et al. (1990). We obtained the total number of AMF spores per gram of soil from 100 g of air‐dried soil suspended in 500 mL water, as described by Gerdemann and Nicolson (1963).

We disinfected the recovered spores, as reported by Mondo et al. (2012). In brief, we washed the pool of spores with two 15‐min rinses in hydrogen peroxide (1 and 50 mM solution, respectively), then a wash with 4% Chloramine‐T and Chlorphenamine 0.3%, followed by a final wash with sterile water. Disinfected spores were then kept at 4°C and used for genomic DNA extraction, microscopic analyses and inoculation of *A. tequilana* plants for the drought experiment.

### DNA extraction, sequencing and downstream analyses

**FIGURE 1:**
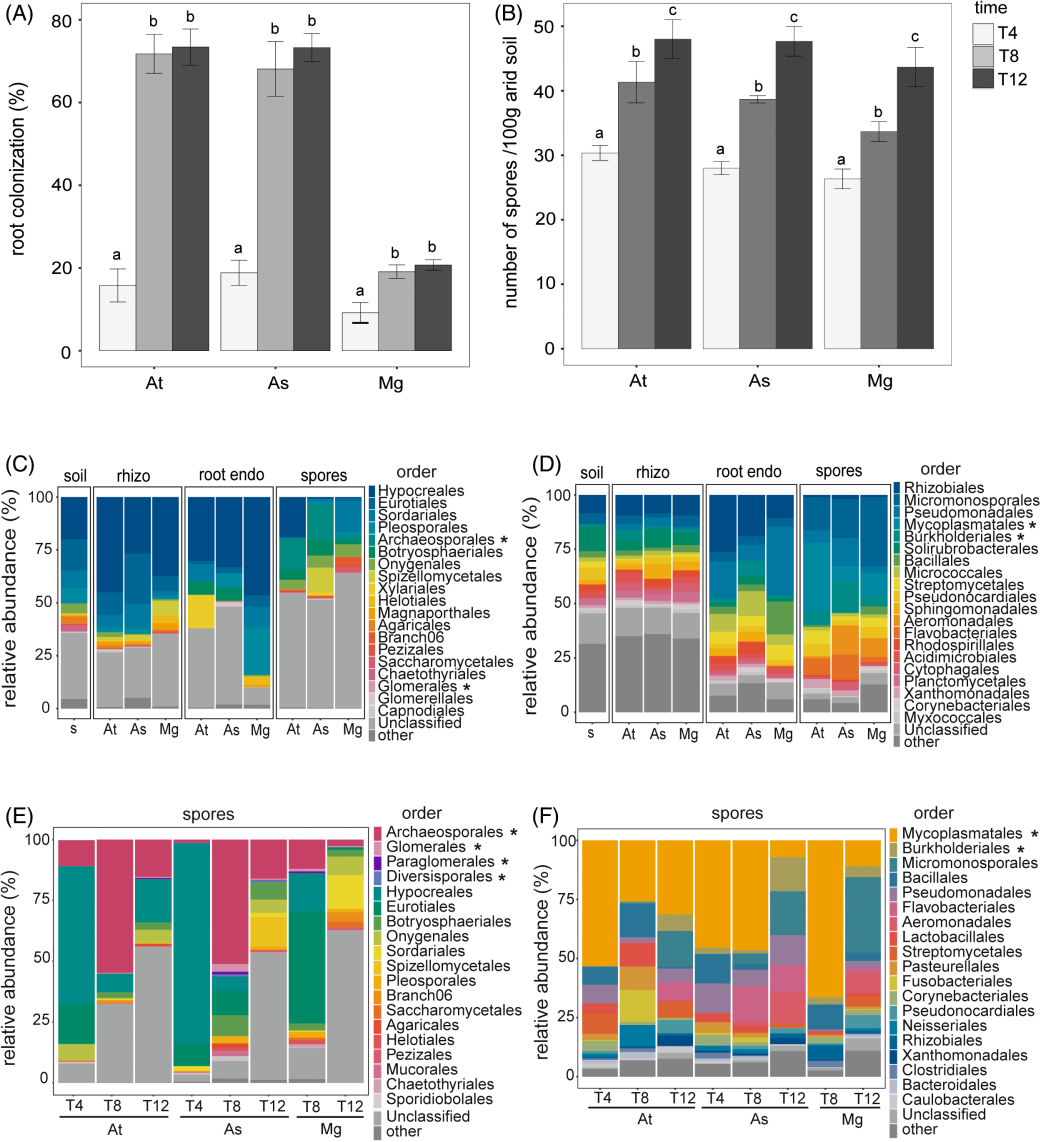
Agaves and cacti are colonized by AMF when grown in arid soil. (A) Root colonization (%) and (B) number of spores/100 g of soil by plant species and sampling time. For (A) *n* = 20, the Kruskal–Wallis test/Dunn with adjusted *P*‐value ≤0.05 was used. For (B) *n* = 3, One‐way ANOVA, Tukey's post hoc test, *P*‐value ≤0.05 was used. Order‐level relative abundance of fungal (C) and bacterial (D) communities across plant compartments/niches (soil, rhizosphere, root endosphere and spores) and plant species at 12 months of growing in arid soil. Order‐level relative abundance of the fungal (E) and bacterial (F) OTUs identified in the spores recovered from each plant species through time. At = *A. tequilana*, As = *A. salmiana* and Mg = *M. geometrizans*. T4, T8 and T12: after 4, 8 and 12 months of growth in arid soil, respectively. Taxa marked with an * represent AMF and reported endofungal bacterial orders, respectively.

We performed amplicon sequencing of the ITS2 and 16S‐rRNA‐V4 gene at T12 when both AMF colonization and the number of fungal spores in the soil were highest (Figure 1A,B). We collected samples and extracted genomic DNA from the bulk soil (only at T0), rhizosphere (the soil firmly attached to the roots, only at T12) and root endosphere (at T12) with the DNeasy power soil kit (Qiagen) from three biological replicates for each plant species as before (Coleman‐Derr et al., 2016; Desgarennes et al., 2014; Fonseca‐Garcia et al., 2016). We also collected fungal spores from the soil associated with each plant species at each of the sampling times (T4, T8 and T12). We pooled genomic DNA from the spores recovered by each plant species at each sampling time since there was little starting material. We extracted gDNA with the Quick DNA Microprep Plus Kit (Zymo). We used the ITS9F (5′‐GAACGCAGCRAAIIGYGA‐3′) and ITS4R (5′‐TCCTCCGCTTATTGATATGC‐3′) and 515F (5′‐GTGCCAGCMGCCGCGGTAA‐3′) and 816R (5′‐GGACTACHVGGGTWTCTAAT‐3′) primers to amplify the ITS2 and 16S‐rRNA‐V4 regions, respectively. We used these primers to compare these datasets with our previous research (Coleman‐Derr et al., 2016, Fonseca‐Garcia et al., 2016).

We randomized the 29 samples (3 from bulk soil, 9 from rhizosphere, 9 from root endosphere and 8 from spores), each having 80–2000 ng of gDNA, in 96 well microplates. We sent the samples to AIM—Advanced Identification Methods in Leipzig, Germany, for amplicon sequencing. Peptide nucleic acids (PNAs) were used for the 16S‐rRNA‐V4 marker to reduce the amplification of plant chloroplast and mitochondrial DNA and increase the number of prokaryotic reads (PNA1: GGCAAGTGTTCTTCGGA and PNA2: GGCTCAACCCTGGACAG, from PNABio) (Lundberg et al., 2013). After ligation, the paired‐end 2 × 300 bp sequencing was performed in an Illumina MiSeq v3 instrument. Negative controls for the PCR and ligations were used.

The Data was demultiplexed in 29 libraries for the ITS2 and the 16S‐rRNA regions. An in‐house command line modified from Flores‐Núñez et al. (2023) was followed to process the raw reads (https://github.com/danielcg91/AMF-Diversity, Table S6).

We analysed the overall sequencing quality using FastQC v0.11.9 (Andrews, 2010). Low‐quality bases and reads were trimmed using Trimmomatic (Bolger et al., 2014), and the resulting read pairs were assembled using FLASH (Magoc & Salzberg, 2011).

Forward and reverse reads were merged to process the 2,291,463 and 3,824,642 paired reads for ITS2 and the 16S‐rRNA‐V4, respectively, using VSEARCH (Rognes et al., 2016).

High‐quality sequences were de‐replicated and clustered in operational taxonomic units (OTUs) using a 95% and 97% similarity cutoff for fungi and prokaryotes, as before (Coleman‐Derr et al., 2016). Then, singletons and chimeras were removed resulting in 2750 and 19,553 OTUs for the ITS2 and the 16S‐rRNA datasets, respectively, using VSEARCH (Table S6) (Rognes et al., 2016).

The OTUs were annotated using the syntax algorithm with a bootstrap threshold of 0.80 using the UNITE database (Abarenkov et al., 2024) for ITS2 and the RDP database (Cole et al., 2014) for 16S‐rRNA‐V4.

Subsequently, we followed the thresholds previously determined by Coleman‐Derr et al. (2016) and Flores‐Núñez et al. (2023). We removed low abundant OTUs, abundant OTUs in the negative controls, non‐microbial OTUs (e.g., Eukaryota, Chloroplast, Viridiplantae) and samples with less than 500 reads. We obtained 1533 and 1522 OTUs for the ITS2 and 16S‐rRNA‐V4 genes, respectively.

### Bioinformatic and statistical analyses

We used R software (version 4.1.3) (Team, 2022) to perform all data analyses and plots. In‐house R scripts and data used for each step have been deposited at github.com/danielcg91/AMF-Diversity.

We used the Shapiro–Wilk test to check data normality in the variables root colonization and the number of spores. Then, we used a Kruskal–Wallis test with a post hoc Dunn test for root colonization, and a one‐way ANOVA with a post hoc Tukey test for the number of spores to compare the differences between plant species.

We first rarefied the OTU table for the diversity analyses to the minimum read count depending on each data set (7068 for fungi and 9321 reads for bacteria). We made OTU abundance plots using the package ggplot2 (Wickham, 2016). We estimated beta diversity using the binary Bray–Curtis distance between samples and calculated the OTU richness and Shannon indexes. We used a permutational multivariate analysis of variance (PERMANOVA) (Anderson, 2001) of binary Bray–Curtis distances with 1000 permutations to identify the main factors that influence the microbial community, then we used the distance matrix to ordinate the samples using a non‐metric multidimensional scaling (NDMS) and calculated the distance to their centroid. We calculated all diversity indexes, distances and ordinations with the package vegan (Oksanen et al., 2022) and MASS (Venables & Ripley, 2002). We tested the differences between groups of samples by plant compartment and plant species using a Kruskal–Wallis test with a post hoc Dunn test. We generated all Venn diagrams with the package ggVennDiagram (Chun‐Hui, 2022).

### Phylogeny reconstructions

We aligned identified and known AMF, MRE and BRE sequences using MUSCLE MEGA v11.0 (Stecher et al., 2020) (Tables S13 and S14). We constructed AMF and bacterial phylogenies employing the GTR + I + Γ nucleotide substitution model implemented in MrBayes 3.2.5 (Ronquist et al., 2012), with a 1,000,000 number of generations and the average standard deviation of split frequencies (<0.01) used as a convergence diagnostic (Figures 3 and S6).

### Co‐occurrence network analyses

We constructed a microbial network to investigate the potential interactions between fungal and bacterial OTUs found in the recovered spores at the three times sampled (Figure S7). We used the R package SParse InversE Covariance Estimation for Ecological Association Inference (SPIEC‐EASI) (Kurtz et al., 2015). We applied the zdk123 example code (https://github.com/zdk123/SpiecEasi) to our data using the default parameters on algorithms for sparse neighbourhood and inverse covariance selection. We detected microbial hubs by their centrality and connectivity (degree >6 and betweenness >750, respectively). Finally, we analysed sub‐networks to show the interactions between AMF and their associated bacteria (Figure 4).

### Multiphoton microscopy

We evaluated the presence and localization of bacteria in recovered and disinfected AMF spores. To achieve this, we used Syto9 (to stain nucleic acids) and FM4‐64 (normally used to stain vacuolar membranes as well as Gram‐positive and negative bacteria) to distinguish bacterial cells from fungal nuclei. We stained spores in a diluted solution (1:100 from stock) of SynaptoRedTM C2 (FM4‐64) (Sigma‐Aldrich, St. Louis, MO) for 1 h at 4°C in darkness. We washed the samples with distilled water for 15 min and stained them with Syto9 LIVE/DEAD® BacLight™ Bacterial Viability Kit (ThermoFisher Scientific, USA) for 15 min at room temperature. We mounted samples in glass slides with PIPES buffer (EMS, Hatfield, PA) and covered them with high‐performance Gold Seal cover glasses 24 × 50 mm No.1 (ThermoFisher Waltham, MA). We detected Syto9 and SynaptoRedTM C2 using a multiphoton microscopic system (LSM 880‐NLO, Zeiss, Germany) with an immersion objective (63×/1.3, NA ∞–0.17, Zeiss Plan NEOFLUAR). The SynaptoRedTM C2 was excited at 543 nm, and the 687–744 nm emission was recorded. Syto9 was excited with an argon laser at 488 nm, and the emission from 506 to 561 nm was recorded. We captured micrographs in CZI format at 1131 × 1131 pixels, RGB colour and we processed them in the software Zeiss Zen Lite 3.6 (blue edition). We used three control strains: *Gigaspora margarita* BEG34+ with its bacterial symbiont Ca. Glomeribacter gigasporarum (*Ca*Gg) served as the positive control (Figure S8a), while *Gigaspora margarita* BEG34 without *Ca*Gg (Figure S8b) and *Trichoderma atroviridae* IMI 206040 (Figure S8c) served as negative controls as they lack bacterial symbionts. As several authors reported, FM4‐64 does not stain mitochondria in fungi exposed to short periods of staining (Fischer‐Parton et al., 2000; Vida & Emr, 1995).

### Drought experiment and measurement of biochemical, root, leaf and biomass plant traits

We conducted this experiment employing micropropagated plants of *A. tequilana*. We first transplanted 2‐month‐old in vitro plants to Cinvestav‐Agave‐soil (tezontle and red oakleaf soil 1:1) for 4 weeks in a growth room (28°C) and then transferred them to the greenhouse (35°C) in trays with the same substrate (58 cm × 36 cm × 9 cm) with six plants per tray. After 1 week in the greenhouse, we inoculated 35 ∓ 4 spores per tray of the characterized and pooled mix (At, As and Mg) of AMF recovered at T12 and kept at 4°C from the previous experiment. We exposed *A. tequilana* plants to four treatments: (1) Well‐Watered (WW) treatment: 70% of relative humidity in soil (RHS); (2) Light Drought (LD): 50% of RHS; (3) Moderate drought (MD): 30% of RHS and (4) Severe Drought (SD): 20% of RHS. Soil humidity was measured online using devices from Meteomex (Winkler, 2021), and trays were watered as needed to keep the RHS at the set point in each of the four treatments. Plants were divided into two groups for each drought treatment: Mock–Control treatment without the AMF application and AMF‐Plants inoculated with the AMF consortia. After 6 months, we carefully harvested plants for further analysis.

We quantified the photosynthetic pigments (chlorophyll a, b, total chlorophylls and carotenoids) using 80% acetone according to (Wellburn, 1994) with the adjustments of (Poorter & de Jong‐Van Berkel, 2010). Absorption was read at 663 (Chl a), 646 (Chl b) and 470 (Carotenoids) on a microplate in Multiskan GO, Thermo scientific®. We determined the proline content by the method of (Bates et al., 1973). Pure toluene was used as blank, while l‐Proline standards (Sigma‐Aldrich) from 0.1 to 10 mM were used for the quantification.

To measure plant morphological traits, we washed harvested plants five times by immersion in distilled water to remove debris. Then, we immediately took a set of photos to automatically assess root architecture with the RhizoVision Explorer software (Version 2.0.3) (Seethepalli et al., 2021) and plant height and leaf area with the ImageJ2 (Fiji) software (Schindelin et al., 2012).

We also quantified the fresh and dry plant biomass. We measured fresh weight on an analytical scale OHAUS® (PA214, ±0.01 g) at 22°C (±3°C) by removing excess moisture with paper towels. We dried samples in an oven at 60°C for 72 h when constant weight was reached. Dry weight was measured in an analytical scale AND® HR‐60 (±0.0001 g) at 23°C (Huang et al., 2017). We quantified AMF root colonization as described above.

## RESULTS

### *Root colonization by AM fungi is threefold higher in* A. tequilana *and* A. salmiana *than in the cactus* M. geometrizans

Our assays confirmed that native AMF effectively colonized the roots of *A. tequilana*, *A. salmiana* and *M. geometrizans* (Figure 1). Mycorrhizal colonization varied over time and between species, ranging from 9% to 73% of root colonization (Figure 1A). Colonization increased from T4 to T8 but not from T8 to T12 (Table S4). At T12, *M. geometrizans* had the lowest root colonization of the three species (21%), while the agaves had the highest with 73%. The number of spores produced by the interaction with the plants ranged between 26 and 48 per 100 g of arid soil (Figure 1B). This number increased over time, reaching the highest values after 12 months with no differences between plant species (Figure 1B, Table S5). We identified arbuscules only at T12 in both agaves, but none in *M. geometrizans* (Figure S3).

### Diverse and dynamic AMF and spores‐associated bacterial communities are associated with desert plants growing in arid soil

We identified 7 and 23 fungal and prokaryotic phyla in our dataset, respectively (Figure S4). Microbial communities in the soil, rhizosphere and root endosphere were dominated by Ascomycota fungi (826 OTUs) from the orders Hypocreales, Eurotiales, Sordariales and Pleosporales (Figure 1C), and by Proteobacteria (433 OTUs) and Actinobacteria (456 OTUs) from the orders Rhizobiales and Micromonosporales, respectively (Figures 1D and S4).

We recognized 61 out of 1533 fungal OTUs as AMF (4%, Table S7) and 550 out of 1522 bacterial OTUs as bacteria in association with the fungal spores (36.14%, Table S8). We labelled this latter group as ‘spores‐associated bacteria’. Archaeosporales (*Ambispora* spp., 9 OTUs) and Glomerales (*Glomus* spp., 22 OTUs) were the most abundant AMF orders (genera) in the spores (Figure 1C,E), while Mycoplasmatales, Burkholderiales, Micromonosporales and Bacillales were the most abundant bacterial orders associated with the spores (Figure 1D,F). Mycoplasmatales were significantly enriched in the spores (16.1%) but were virtually absent (<0.01%) in the root endosphere, rhizosphere and soil (Figure 1D, Table S9). The order Burkholderiales showed an increased prevalence, although not statistically significant, in the spores (8.7%) compared with the root endosphere (2.6%), rhizosphere (1%) and soil (0.85%, Figure 1D, Table S9). Analyses focused on the spores reflected that the fungal and bacterial communities were dynamic and varied along time and plant species (Figure 1E,F). Spores belonging to the Archaeosporales peaked at T8 in the three species and showed an average of 20.5% of relative abundance across the three sampling times, while Glomerales averaged 0.7%, Paraglomerales 0.3% and Diversisporales 0.1% of the relative abundance, respectively. Members of the Hypocreales were important in the spores, showing an average of 23% relative abundance (Figure 1E). Mycoplasmatales were persistently abundant within the spores through time, with an average relative abundance of 35.7%, while Burkholderiales had an average of 4.2% (Figure 1F).

**FIGURE 2:**
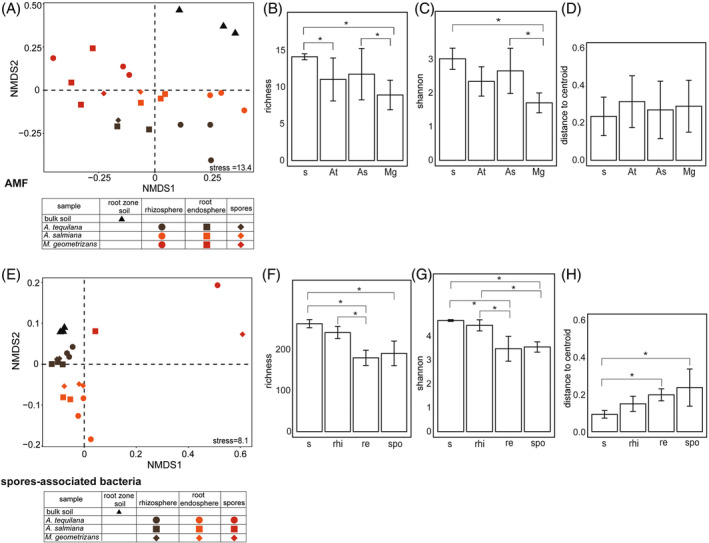
Nonmetric multidimensional scaling (NMDS) plots for Bray–Curtis distances and diversity indexes of the (A–D) AMF and (E–H) spores‐associated bacterial communities associated with *A. tequilana*, *A. salmiana*, and *M. geometrizans*. Bars represent the mean ± SD of the observed OTUs richness, Shannon index, and distance to the centroid of the dissimilarities. Red asterisks indicate significant changes between treatments. Kruskal–Wallis test/Dunn test: adjusted *P*‐value ≤0.05 was used. At = *A. tequilana*, As = *A. salmiana*, and Mg = *M. geometrizans*, s = soil, rhi = rhizosphere, re = root endosphere, spo = spores.

Our PERMANOVA and NDMS analyses of the binary Bray–Curtis distances between samples showed a strong influence by the plant species in the AMF communities while the plant compartment/niche chiefly influenced the spores‐associated bacteria subset (*R* ^2^ = 0.34 and 0.57, *P* < 0.001, respectively, Figure 2A,E, Tables S10 and S11). The AMF communities in the arid soil and *A. salmiana* were more diverse, both in species richness and in the Shannon index, than in *M. geometrizans* (Figure 2B–D). In the spores‐associated bacteria subset, the soil and rhizosphere exhibited the highest alpha diversity, contrary to the root endosphere and spores where it was significantly lower (Figure 2F–H). However, when the full fungal and prokaryotic datasets were considered, the plant compartment/niche was the strongest factor influencing community assembly (*R* ^2^ = 0.53 and 0.65, *P* < 0.001, respectively) (Figure S5a,e; Tables S10 and S11), having the soil and rhizosphere a greater alpha diversity than the root endosphere and spores in both datasets (Figure Sb–d,f–h).

### MRE and novel BRE taxa are associated with native arid AMF

**FIGURE 3:**
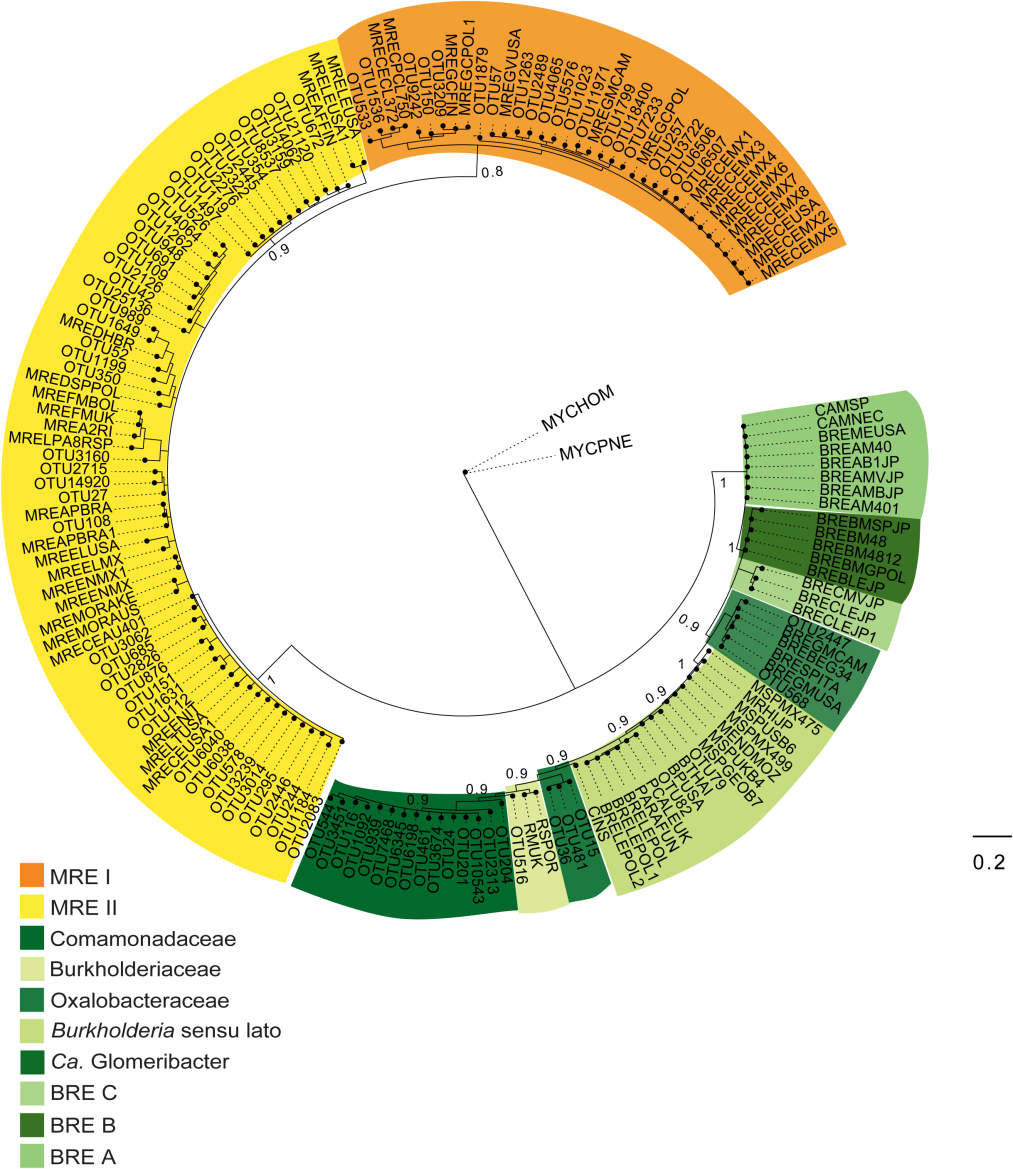
Bayesian phylogeny of the Mycoplasmatales‐related endosymbionts (MRE) and Burkholderiales‐related endosymbionts (BRE) identified in recovered AMF spores. MrBayes analyses indicate the formation of supported clades of spores‐associated bacteria coloured by their phylogenetic identity. MREs are coloured yellow and orange. All Burkholderiales clades are coloured in different greens. All sequences labelled as ‘OTU##’ were generated in this study.

We filtered and selected all the bacterial OTUs belonging to the orders Mycoplasmatales and Burkholderiales associated with the spores since these are the most likely endosymbionts of AMF (Table S12) (Uehling et al., 2023). Our phylogenetic analysis of these 90 OTUs showed two MRE clusters: MRE I with 20 OTUs from this study, and MRE II with 47. Remarkably, the OTUs associated with BRE did not group with any of the groups BRE A, B and C previously reported (Amses et al., 2023; Okrasinska et al., 2021; Takashima et al., 2018) (Figure 3, Table S13). We recovered two OTUs that are similar to the *Candidatus* Glomeribacter group and only present in the spores, and two OTUs within *Burkholderia* sensu lato. Our data revealed three new groups related to the order Burkholderiales: 14 novel OTUs within the Comamonadaceae, 2 within Burkholderiaceae and 3 within the Oxalobacteraceae. These bacterial OTUs might be associated with AMF in arid environments (Table S14).

We inferred possible connections among fungi and bacteria in the spores using co‐occurrence networks. This network, representing the microbe‐microbe interactions in the spores, included 259 vertices and 718 edges (Figure S7). We determined 453 positive edges, 265 negative edges and 46 hubs (8 from fungi and 38 from bacteria) (Table S15). From these, four hubs were associated with AMF (FOTU_490, FOTU_541, FOTU_1134, FOTU_1854), suggesting that these AMF OTUs might be pivotal for the root microbial community in agaves and cacti (Table S16). We focused on the connections between the AMF nodes and the putative endofungal bacteria (Figure S7).

**FIGURE 4:**
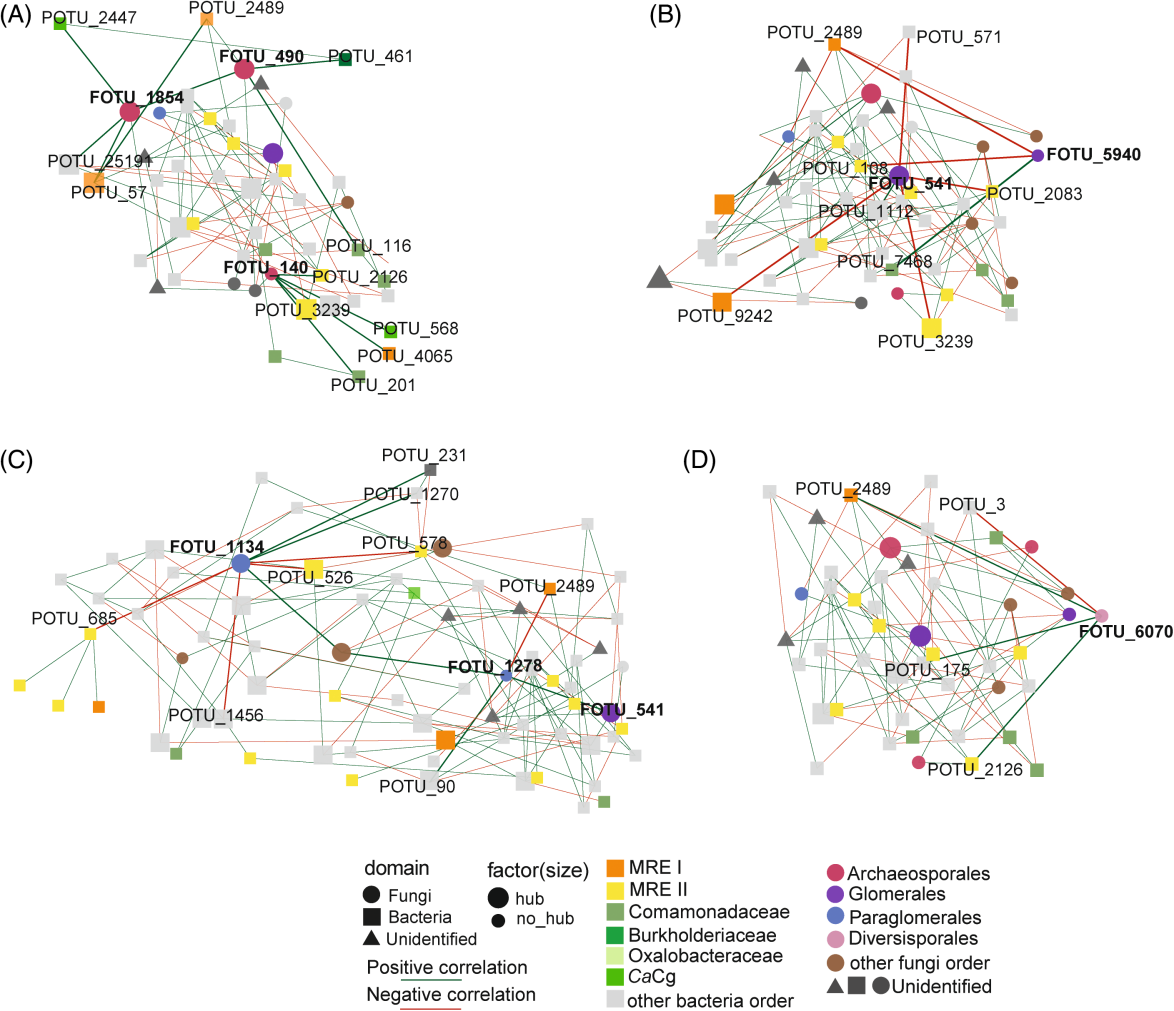
Co‐occurrence sub‐networks of fungal and bacterial OTUs from recovered spores focused on AMF. Sub‐networks focused on OTUs identified as (A) Archaeosporales, (B) Glomerales, (C) Paraglomerales and (D) Diversisporales. Vertices (Nodes, OTUs) are coloured based on the AMF order and sized based on their classification (Hub, no hub). Edges (interactions) are coloured based on their type of interaction (green‐positive/red‐negative) and their weight.

We found positive connections between the most abundant order Archaeosporales (*Ambispora*, FOTU_140, **FOTU_490** and **FOTU_1854**) with MRE I, MRE II and the specific node of *Candidatus* Glomeribacter (POTU_2447), Comamonadaceae, Burkholderiaceae and Pseudomonadales (POTU_25191) (Figure 4A).

The order Glomerales (*Glomus*, **FOTU_541** and FOTU_5940) had positive connections with Comamonadaceae and Pseudomonadales (POTU_1112), but negative connection with members of both MRE clades and Xanthomonadales (POTU_571) (Figure 4B).

The order Paraglomerales (*Paraglomus*, **FOTU_1134** and FOTU_1278) exhibited positive connections with Rhizobiales (POTU_1270), Enterobacteriales (POTU_90) and Alphaproteobacteria (POTU_231), but negative with members of both MRE clades, Bacillales (POTU_14) and Bacteroidales (POTU_2220) (Figure 4C).

The order Diversisporales (*Acaulospora*, FOTU_6070) showed positive connections with MRE I (POTU_2489), MRE II (POTU_2126) and Pseudomonadales (POTU_175) (Figure 4D).

### Bacterial cells are present inside arbuscular mycorrhizal fungi from arid soil

**FIGURE 5:**
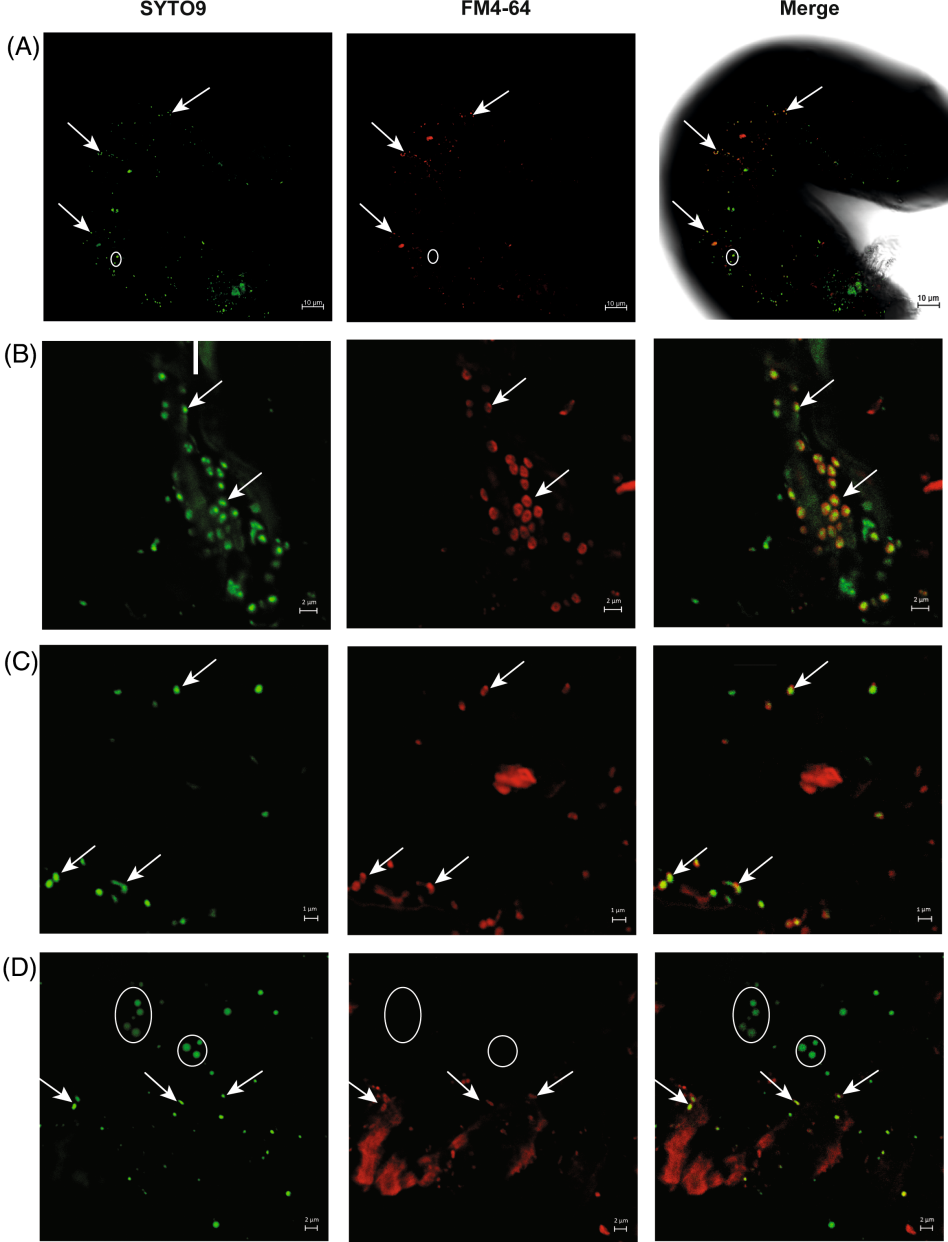
Multiphoton microscopy of AMF spores and their associated bacteria dyed with SYTO9 and FM4‐64. (A) A single mycorrhizal spore shows both nuclei and intracellular bacterial cells. Arrows were used to indicate the presence of bacteria where signals of SYTO9 and FM4‐64 were visible, and circles were used to indicate the presence of fungal nuclei, where only the SYTO9 signal was visible. (B) A cluster of coccoid‐shaped bacteria (likely MRE) within an AMF spore. (C) Rod‐shaped bacteria (likely BRE) with different sizes are shown within the spore. (D) The presence of bacteria (arrows, green and red dots) and fungal nuclei (circles, only green dots) are shown.

We confirmed the presence of bacteria inside the recovered AMF spores employing multiphoton microscopy and two different dyes, Syto9 and FM4‐64, as depicted in Figure 5. The combination of these dyes allowed us to distinguish fungal nuclei (marked in circles, only coloured by Syto9) from bacterial cells (pointed by arrows, coloured by both Syto9 and FM4‐64) in the most abundant AMF spores, potentially *Ambispora* spp. These micrographs showed that bacterial cells were embedded in the cytoplasm of the spores (Figure 5A). Also, we observed coccoid‐shaped (likely MRE, Figure 5B,C) and rod‐shaped bacteria (likely BRE, Figure 5A,B,D) with variable sizes (0.5–1 μm). We observed the presence of fungal nuclei (marked in circles, Figure 5A,D) with variable sizes (1.5–3 μm, Figure S8). All the AMF spores analysed by microscopy (ca. 30 spores) revealed the presence of endofungal bacteria, suggesting a high prevalence of these symbionts in AMF, as shown in different environments (Lastovetsky et al., 2018, 2024).

### *AMF inoculation enhances drought tolerance in* A. tequilana

**FIGURE 6:**
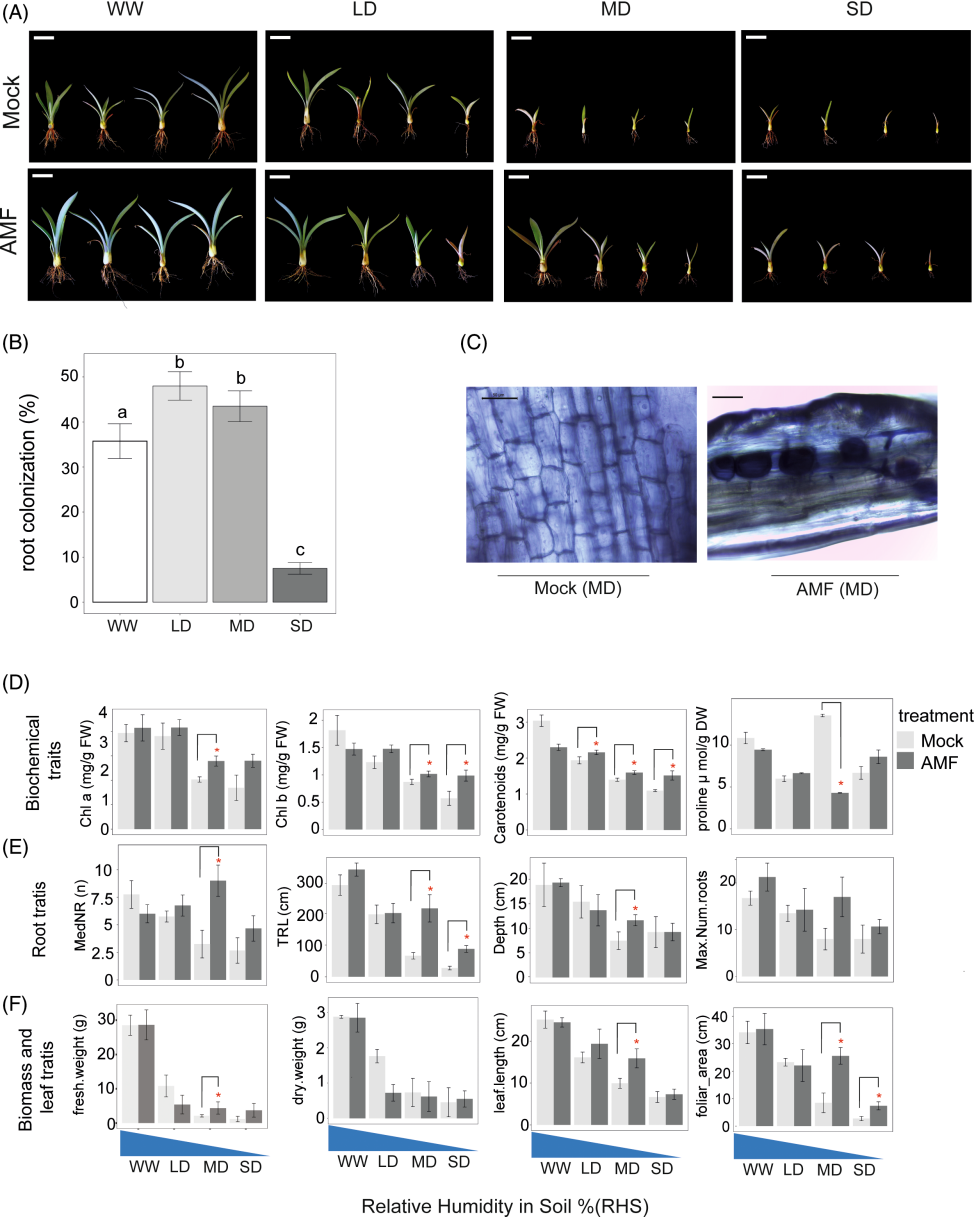
AMF increase drought tolerance in *A. tequilana*. (A) Phenotypes of *A. tequilana* plants submitted for 6 months to four drought treatments with or without being inoculated by native AMF recovered at T12. (B) Root colonization (%) in AMF‐inoculated *A. tequilana* plants after 6 months of drought in each of the four treatments. Kruskal–Wallis test/Dunn with adjusted *P*‐value ≤0.05 was used. (C) Representative micrographs of MOCK and AMF‐inoculated plants of *A. tequilana* under MD (30% of RHS) (Scale bar = 50 mm). Effects of drought stress on (D) Biochemical traits (Chl a, Chl b, Carotenoids and proline), (E) Root traits (MedNR = Median Number of roots, TRL = Total root length, Depth, and Maximum Number of roots), (F) Biomass and leaf traits (fresh and dry weight, leaf length and foliar area) with Mock and AMF Inoculation. Data presented are the means (±SE) of three replicates and the asterisks indicate significant differences; One‐way ANOVA, Tukey's post hoc test, and *P*‐value ≤0.05 were used.

To gain some functional insights into these native AMF in desert CAM plants, we designed a greenhouse study applying four different treatments, well‐watered WW (70% relative humidity in soil (RHS)), low drought LD (50% RHS), middle drought MD (30% RHS) and severe drought SD (20% RHS), in micropropagated plants of *A. tequilana* during 6 months (Figure 6).

As observed in Figure 6A, the vigour and size of *A. tequilana* plants decreased with increasing levels of drought. Also, plants inoculated with AMF showed a healthier phenotype than Mock plants (Figure 6A). We confirmed that plants inoculated with AMF were indeed colonized by these fungi observing the presence of vesicles (V) and hyphae (H), while the Mock plants were not (Figure 6C). However, we observed that the drought treatments influenced the % of AMF root colonization (Figure 6B), being highest in the LD and MD treatments (ca. 40%–50%) and lowest in SD (<10%).

The biochemical, root, leaf and biomass plant traits showed that under WW, no differences between Mock and AMF‐inoculated plants existed, suggesting a likely commensal role of AMF under these circumstances (Figure 6D–F). Only in MD (30% of RHS), we consistently observed that AMF alleviated the negative effects of drought in the plants of *A. tequilana*, increasing the quantity of chlorophyll a, b and carotenoids in the leaves, while reducing proline (Figure 6D). Also, the roots and leaves of the AMF plants under MD were larger both in length/depth and area (Figure 6E,F), and had fresher biomass (Figure 6F). These results support the idea of mutualistic interaction between plants of *A. tequilana* and their native AMF under these harsh conditions.

## DISCUSSION

Our study showed that agaves, including the cultivated *A. tequilana*, can be naturally and highly colonized by AMF from arid soils, while in the roots of the cactus *M. geometrizans*, Glomeromycotina fungi are less abundant and diverse. The tendency for higher AMF colonization in agaves than in cacti growing in the same soil has been previously observed (Cui & Nobel, 1992). Also, our study did not observe any significant changes in AMF root colonization after 8 and 12 months in young plants. However, a longer experiment would be necessary to track the dynamics of the AMF community along the development of agaves and cacti, as yearly growth outbreaks dominate these long‐living plant species (Nobel, 1998).

Our molecular data at T12 confirmed the lower AMF colonization of the cactus *M. geometrizans* in comparison with the two agaves. Intriguingly, the AMF genera that dominated the recovered spores were *Ambispora* and *Glomus*, while in our previous studies, we chiefly detected *Glomus* and *Entrophosphora*, albeit in the root endosphere of older plants (Coleman‐Derr et al., 2016; Fonseca‐Garcia et al., 2016). This difference might be due to the developmental stage of the sampled plants, the distinct abiotic conditions in which plants were growing, the succession of AMF communities (Gao et al., 2019), or a combination of them.

Several studies have reported the diversity of AMF associated with agaves and cacti based on morphological characters (Abrego et al., 2020; Carballar‐Hernández et al., 2013; Cui & Nobel, 1992; Gardezi et al., 2022; Hernandez‐Cuevas et al., 2023; Lahbouki, Ben‐Laouane, et al., 2022; Montoya‐Martínez et al., 2019; Ochoa‐Meza et al., 2009). However, this study is the first one to use metabarcoding to identify mycorrhizal fungi and their potential associations with endofungal bacteria in these important plant species native to the Mexican drylands.

Two other molecular studies on AMF in crops developing in arid environments in Mexico that included peppers, soybean, oranges, onion, walnut, alfalfa and maize (Guardiola‐Márquez et al., 2022; Senés‐Guerrero et al., 2020), together with ours, suggest that AMF communities associated with plants might be selected by the plant species, and also likely, by the biogeography (Coleman‐Derr et al., 2016; Fonseca‐Garcia et al., 2016; Martínez‐García & Pugnaire, 2011; Pagano et al., 2011; Senés‐Guerrero et al., 2020). However, the level of resolution is still very low to determine to which extent the ‘same’ AM fungi colonize several plants in the same or at distant sites.

Our phylogenetic and network analyses revealed specific associations of AMF taxa with known endobacterial lineages belonging to the orders Mycoplasmatales (MRE) and several novel Burkholderiales (BRE), as well as positive and negative connections with diverse bacteria from other phyla. These results, along with those reported from other studies (Agnolucci et al., 2015, 2019; Basiru et al., 2023; Emmettet al., 2021; Faghihinia et al., 2023; Lastovetsky et al., 2018, 2024; Mondo et al., 2012; Naumann et al., 2010; Robinson et al., 2021; Toomer et al., 2015; Ujvari et al., 2021; Wang et al., 2022), support the idea that in nature, diverse bacteria might transiently look for food and shelter inside Glomeromycotina fungi, while some others, such as MRE and BRE, might be adapted to permanently reside inside fungal hyphae and spores. However, we must recognize that our knowledge of these complex plant–fungal–bacterial interactions is still limited (Uehling et al., 2023).

Our microscopic observations from about 30 AMF spores recovered from *A. tequilana*, *A. salmiana* and *M. geometrizans* showed that they were internally colonized by coccoid and/or rod‐shaped bacteria. Unfortunately, we have been unable to identify bacteria‐free spores so far, and we also had problems unequivocally confirming the identity of the most abundant AMF spores (likely *Ambispora* spp., based on our molecular data and the morphological spore characters). In the near future, in situ hybridization experiments, or single‐spore DNA sequencing, could help confirm the predicted connections among AMF and specific endobacterial lineages.

Finally, to gain functional insights into the impact of the AMF symbiosis in CAM plants under the effects of climate change, we applied four levels of drought (WW, LD, MD and SD) for 6 months to Mock and AMF inoculated micropropagated young plants of *A. tequilana*. This experiment revealed that the strength of the interaction (% of AMF root colonization) and the outcome of this symbiosis are dependent on the abiotic context. In WW conditions, no phenotypic effect could be measured on the plant holobiont, while a clear positive, mutualistic effect was noticeable under MD. Although CAM‐photosynthetic plants are considered well‐adapted to deal with drought, the SD treatment showed that under these circumstances, the interaction with AMF fungi is drastically reduced, and less clear are also the benefits. Further work is necessary to elucidate the role of endofungal bacteria in this response. In sorghum [*Sorghum bicolor* (L.) Moench], a C4‐photosynthetic cereal, pre‐flowering and post‐flowering drought disrupted the AMF symbiosis (Varoquaux et al., 2019). These results and ours portray the ‘unforeseeable’ risks that living organisms on our planet, including drought‐tolerant plants, are facing due to the effects of global warming and climate change.

In arid desert plants, an augmented osmotic adjustment is essential to counteract the negative effects of drought stress (Madouh & Quoreshi, 2023). Our data revealed that, in general, drought reduced the carotenoids and chlorophylls, root and leaf traits, and biomass in *A. tequilana*. However, AMF colonization tended to increase carotenoids and chlorophylls. These findings align with the studies conducted on *Opuntia ficus‐indica* (Lahbouki, Ben‐Laouane, et al., 2022) and other important crops (Begum et al., 2019; Chitarra et al., 2016; Mathur et al., 2019; Soussani et al., 2023). Proline (Pro) is an important osmolyte that reduces ROS. Proline concentration has shown a variable behaviour in AMF colonized plants, increasing, reducing, or maintaining its concentration under water deficit (Bogati & Walczak, 2022; Jimenez‐Perez et al., 2022). Our results showed an inconsistent accumulation of proline, having a high peak under MD. However, in this treatment, AMF‐colonized plants accumulated 70% less proline than Mock plants, suggesting a reduced demand for this osmoprotectant. These results align with previous reports (Manoharan et al., 2010; Porcel & Ruiz‐Lozano, 2004; Varoquaux et al., 2019; Wu et al., 2017), in which AMF‐inoculated plants accumulated less proline in leaves than Mock plants under severe water stress conditions.

In sum, our study sheds light on the diversity of AMF and their associated bacteria in the desert CAM plants *A. tequilana*, *A. salmiana* and *M. geometrizans*. We also identified that severe drought limits the symbiosis between *A. tequilana* and native AMF, reducing the positive outcomes for the plant holobiont. However, the role of bacteria living inside AMF remains cryptic and needs to be investigated. A deeper understanding of the ecological roles of the tripartite symbiosis between plants‐AMF‐endobacteria will enable the development of tailored approaches to preserve the diversity and functionality of arid ecosystems while sustaining agriculture and human life in these regions.

## AUTHOR CONTRIBUTIONS

**Jose Daniel Chávez‐González:** Conceptualization (equal); data curation (lead); formal analysis (lead); investigation (lead); methodology (lead); validation (equal); visualization (lead); writing – original draft (lead); writing – review and editing (lead). **Víctor M. Flores‐Núñez:** Data curation (supporting); formal analysis (supporting); methodology (supporting); software (lead); writing – review and editing (supporting). **Irving U. Merino‐Espinoza:** Formal analysis (supporting); investigation (supporting); methodology (supporting); visualization (supporting); writing – review and editing (supporting). **Laila Pamela Partida‐Martínez:** Conceptualization (equal); formal analysis (supporting); funding acquisition (lead); investigation (supporting); methodology (supporting); project administration (lead); supervision (lead); validation (equal); visualization (supporting); writing – original draft (equal); writing – review and editing (lead).

## CONFLICT OF INTEREST STATEMENT

All authors declare that they have no conflicts of interest.

## Supporting information


**Data S1.** Supporting Information.

## Data Availability

The generated raw reads have been deposited under the SEA accessions SRR23871836 to SRR23871855 (Bioproject PRJNA944644).
