# Supplementary material for: Desert plants, arbuscular mycorrhizal fungi and associated bacteria: Exploring the diversity and role of symbiosis under drought
Source: Environ Microbiol Rep. 2024 Jul 9;16(4):e13300. doi: 10.1111/1758-2229.13300 (PMC11231939; doi:10.1111/1758-2229.13300)
Supplement: Supplementary file 1 — Data S1. Supporting Information. [file EMI4-16-e13300-s001.pdf]

The following Supporting Information is available for this article:

Index for Figures

Figure S1. AMF richness and soil collected in San Felipe, Gto., México.
Figure S2. Pictures of CAM plants sampled after 4, 8, and 12 months of growing in SF soil.
Figure S3. Micrographs of At, As, and Mg roots colonized by native AMF from San Felipe at T4, T8, and T12.
Figure S4. Identity and distribution of fungal and bacterial by plant compartment and species.
Figure S5. Nonmetric multidimensional scaling (NMDS) plots for Bray–Curtis distances and diversity indexes of the fungal and bacterial communities associated with *A. tequilana*, *A. salmiana*, and *M. geometrizans* species.
Figure S6. Phylogenetic analysis of ITS2 of AMF OTUs obtained in this study combined with the sequences from UNITE and with the best NCBI hit database.
Figure S7. Co-occurrence network of spores.
Figure S8. Multiphoton microscopy of control strains.

Index for Tables

Table S1 PERMANOVA analysis of the AMF communities associated with agaves and cacti in 2012.
Table S2 Kruskal-Wallis/Dunn test of the average relative abundance of AMF total richness associated with agaves and cacti among the Locations investigated.
Table S3 Linear regression analysis among total richness and phosphorus concentration where native agaves and cacti are growing.

Table S4 Kruskal-Wallis/Dunn test of the root colonization by time and plant species.

Table S5 Kruskal-Wallis/Dunn test of the number of spores recovered from each plant species.

Table S6 Sequencing statistics from row data to the measurable and rarefied OTU tables.

Table S7 List of the 61 AMF OTUs detected in this study.

Table S8 List of the 550 AMF spores-associated bacterial OTUs detected in this study.

Table S9 Kruskal-Wallis test/Dunn test of the spores-associated bacterial OTUs enrichment in spores compared with soil, rhizosphere, and root endosphere.

Table S10 PERMANOVA analyses of the microbial communities associated with *A. tequilana*, *A. salmiana,* and *M. geometrizans* growing in the same arid soil for 12 months.

Table S11 Kruskal-Wallis/Dunn test of richness, Shannon index and distance to centroid by plant compartment and plant species of agaves and cacti.

Table S12 List of the MRE and BRE OTUs found in this study.

Table S13 List of the 16S rRNA-V4 gene sequences used for the phylogenetic analysis included in Figure 3.

Table S14 Molecular identification of the AMF OTUs obtained in this study and the list of the ITS2 gene sequences used for phylogenetic analysis included in Figure S7.

Table S15 Network metrics used to generate the co-ocurrence network from the spores.

Table S16 Identified nodes and hubs in the co-occurrence network from the spores.

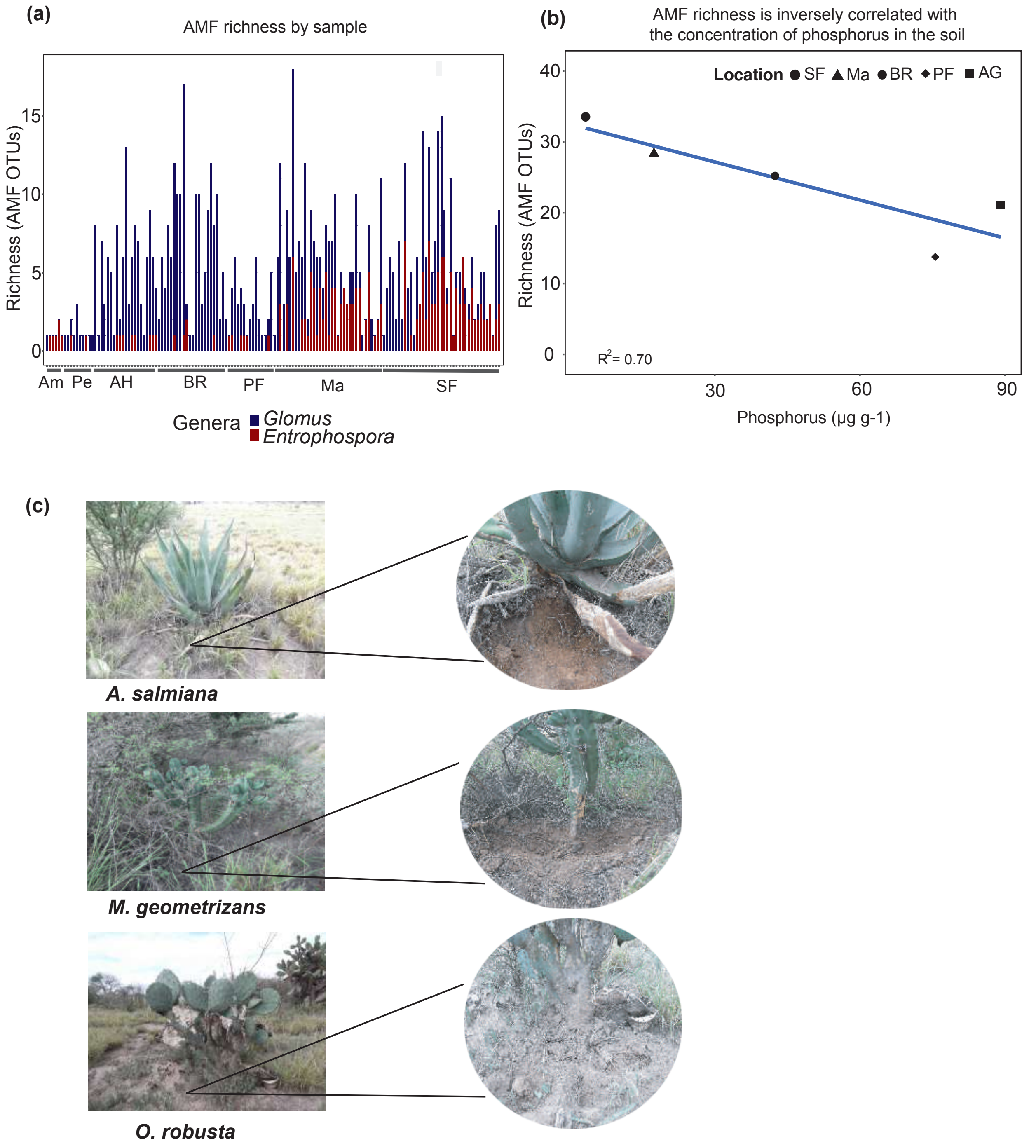

Figure S1. AMF richness and soil collected in San Felipe, Gto., México. (a) Total richness of the AMF OTUs found in fungal communities associated with native and wild agaves and cacti in samples from 2012; Kruskal-Wallis test/Dunn with adjusted p-value ≤ 0.05 was used. No significant differences were found. (b) Linear regression with the total richness of AMF OTUs and phosphorus concentrations of soil where native plants were sampled. Adjusted R-squared = 0.70 (c) Semi-arid soil and root zone soil of sympatric CAM plants in San Felipe, Gto., México.

that grow naturally at San Felipe (SF), Guanajuato were collected for experiments (Coleman-Derr *et al.*, 2016; Fonseca-Garcia *et al.*, 2016). Am=Amatitán, Pe=Pénjamo, AH=Agave Hill, BR= Boyd Ridge, PF=Pinyon Flats, Ma=Magueyal, SF=San Felipe.

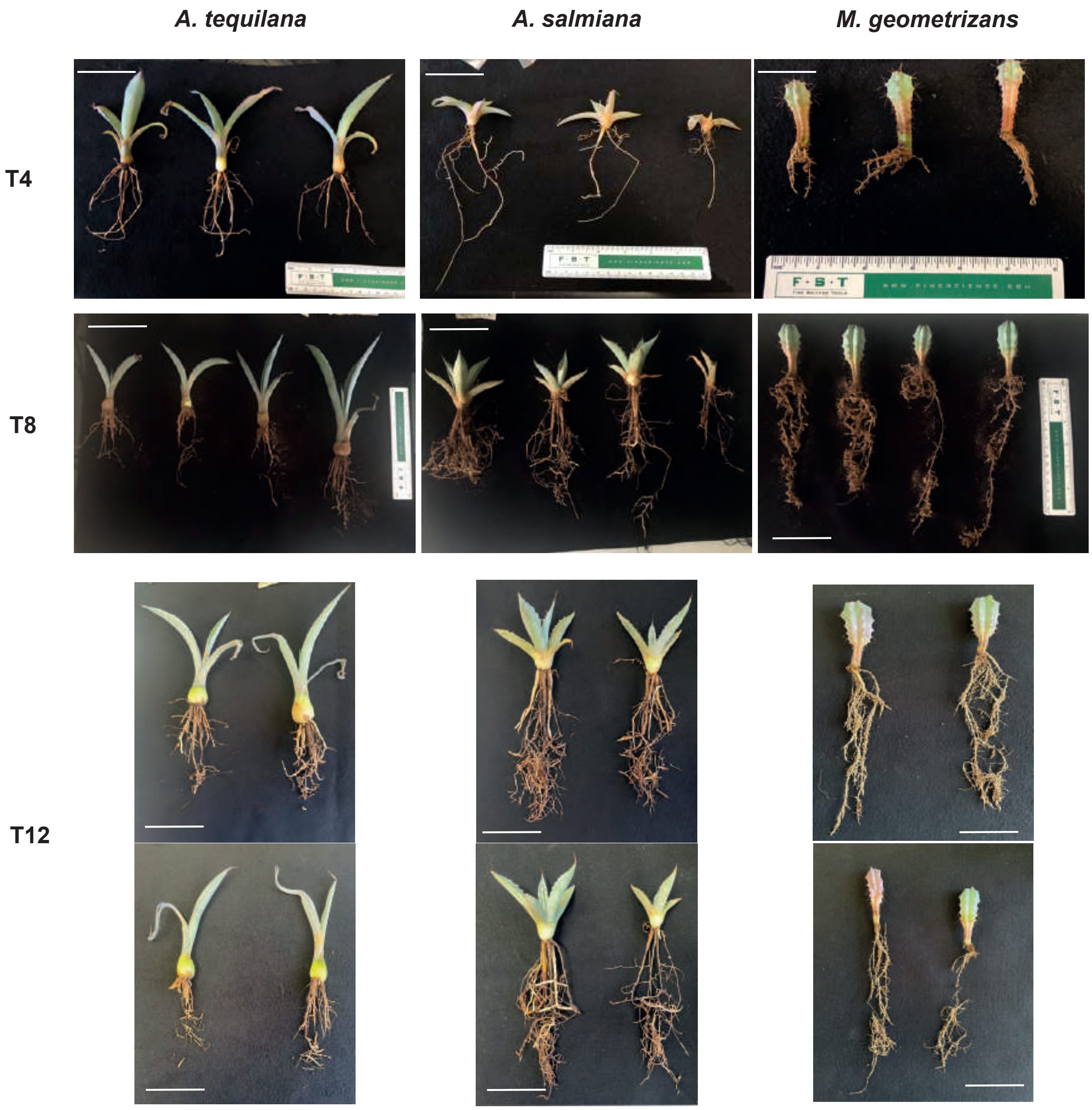

Figure S2. Pictures of CAM plants sampled after 4, 8, and 12 months of growing in SF soil. n=4 plants per sampling (scale bar=5 cm).

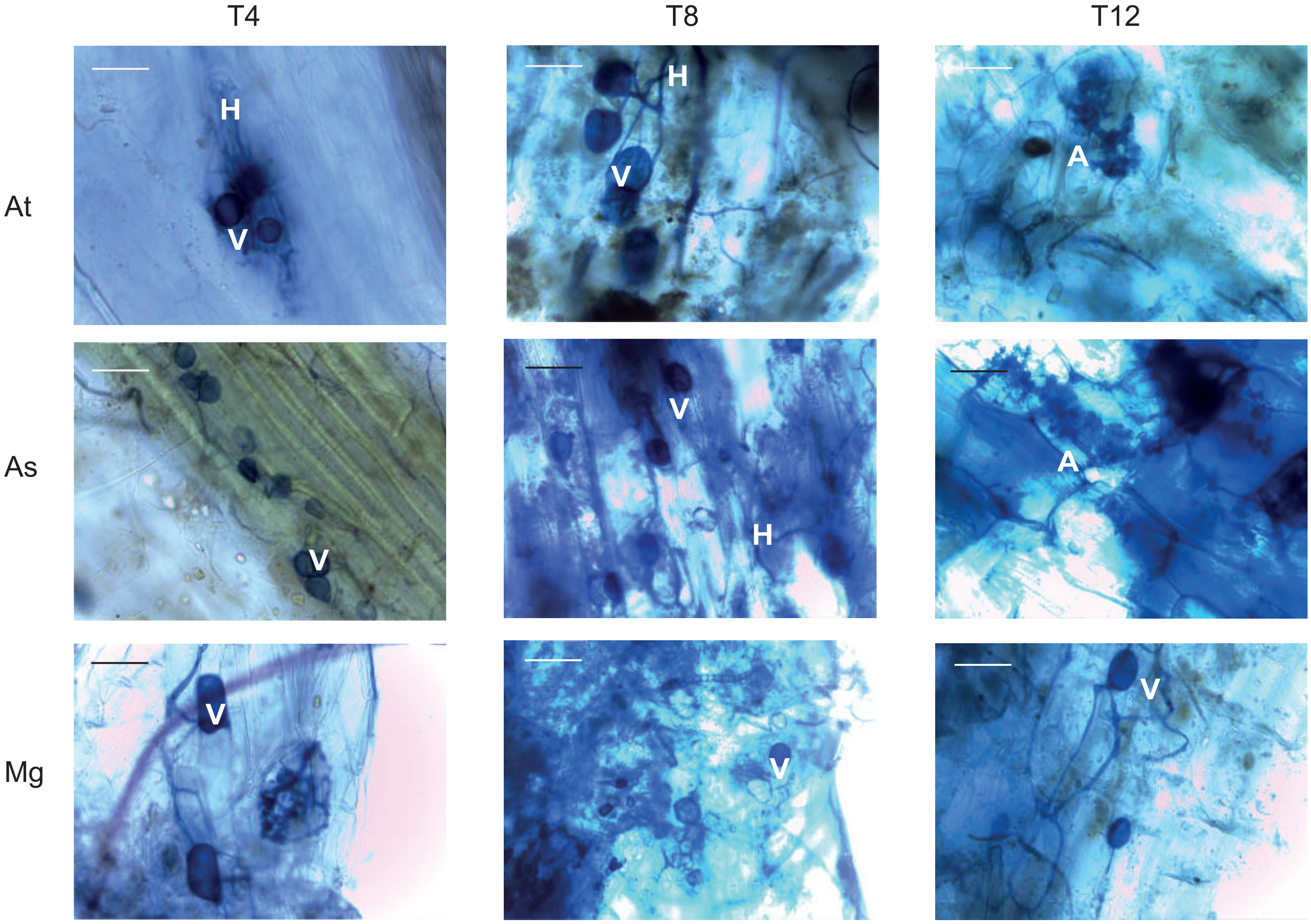

,

Figure S3. Micrographs of At, As, and Mg roots colonized by native AMF from San Felipe at T4, T8, and T12. In all cases: At= *A. tequilana*, As= *A. salmiana*, and Mg= *M. geometrizans*. V=vesicles, H=hyphae, A=arbuscules. (scale bar=50 μm).

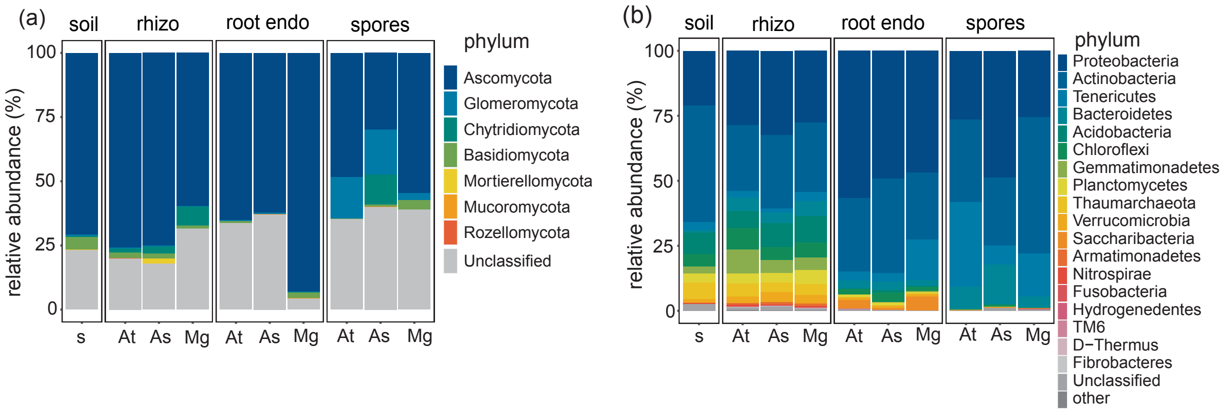

Figure S4. Identity and distribution of fungal and bacterial by plant compartment and species. Phylum-level relative abundance of fungal (a) and bacterial (b) communities across plant compartments at 12 months of growing in arid soil. At= *A. tequilana*, As= *A. salmiana,* and Mg= *M. geometrizans*, rhizo=rhizosphere, root endo = root endosphere

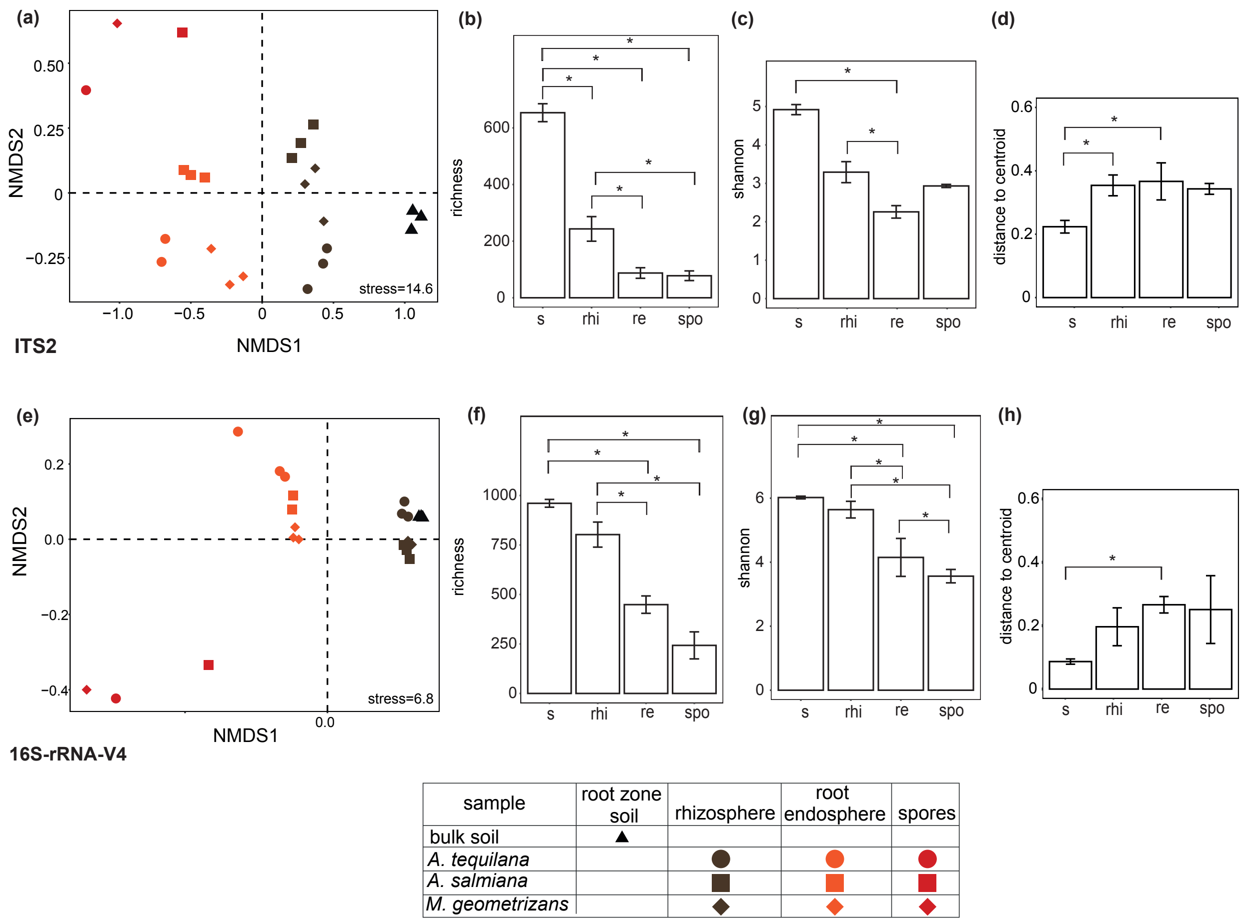

Figure S5. Nonmetric multidimensional scaling (NMDS) plots for Bray–Curtis distances and diversity indexes of the (a-d) fungal and (e-h) bacterial communities associated with *A. tequilana*, *A. salmiana*, and *M. geometrizans* species. Bars represent the mean ± sd of the observed OTUs richness, Shannon index, and distance to the centroid of the dissimilarities. Asterisks indicate significant changes between plant compartments; Kruskal-Wallis test/Dunn test: adjusted p-value ≤ 0.05 was used. At= *A. tequilana*, As= *A. salmiana,* and Mg= *M. geometrizans*, s= soil, rhi=rhizosphere, re = root endosphere, spo = spores.

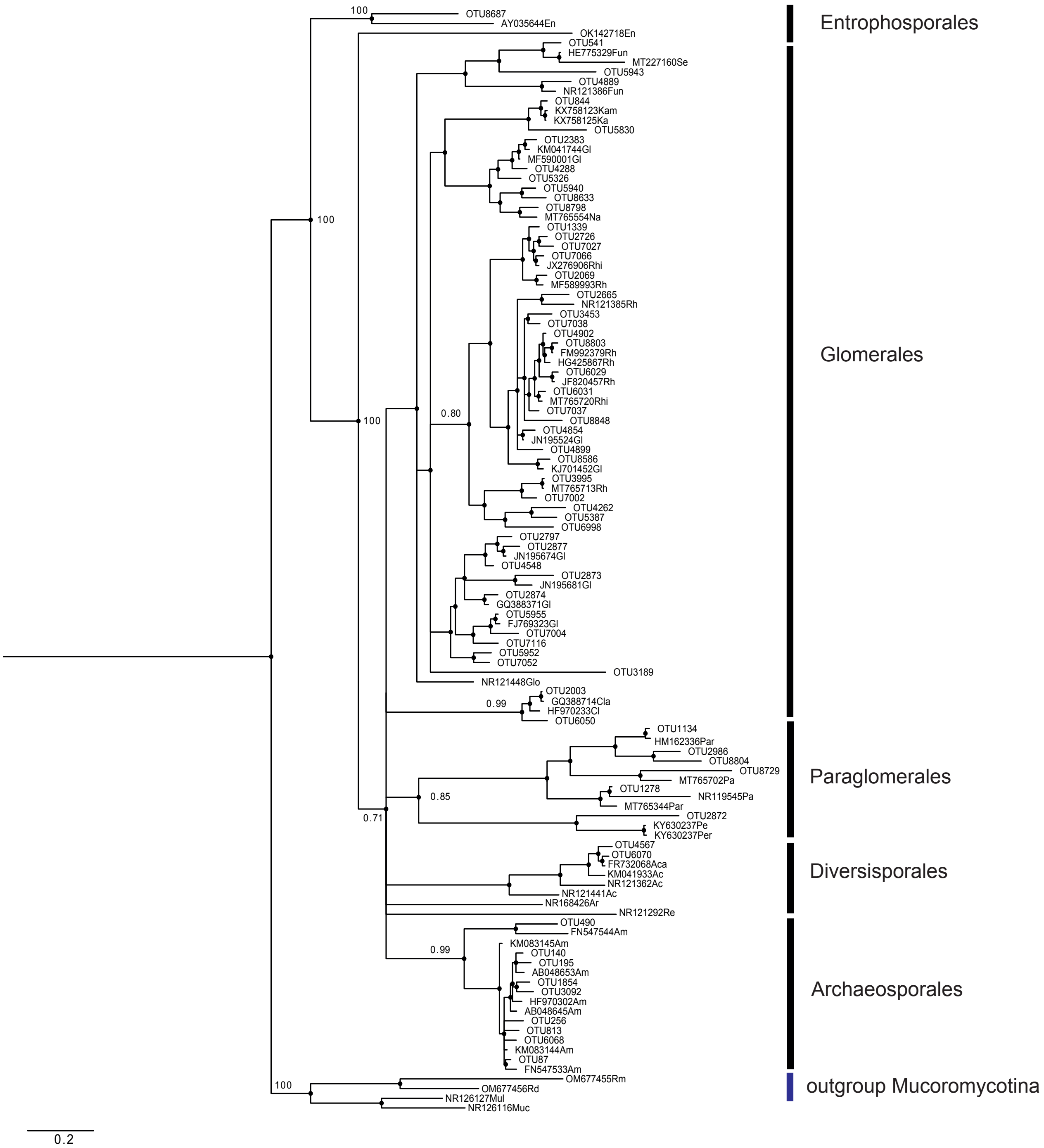

Figure S6. Phylogenetic analysis of ITS2 of AMF OTUs obtained in this study. Mr. Bayes analysis confirmed the taxonomic position of the AMF sequences within the Glomeromycotina sub-phylum. Mucoromycotina sequences were used as outgroup (Table S14).

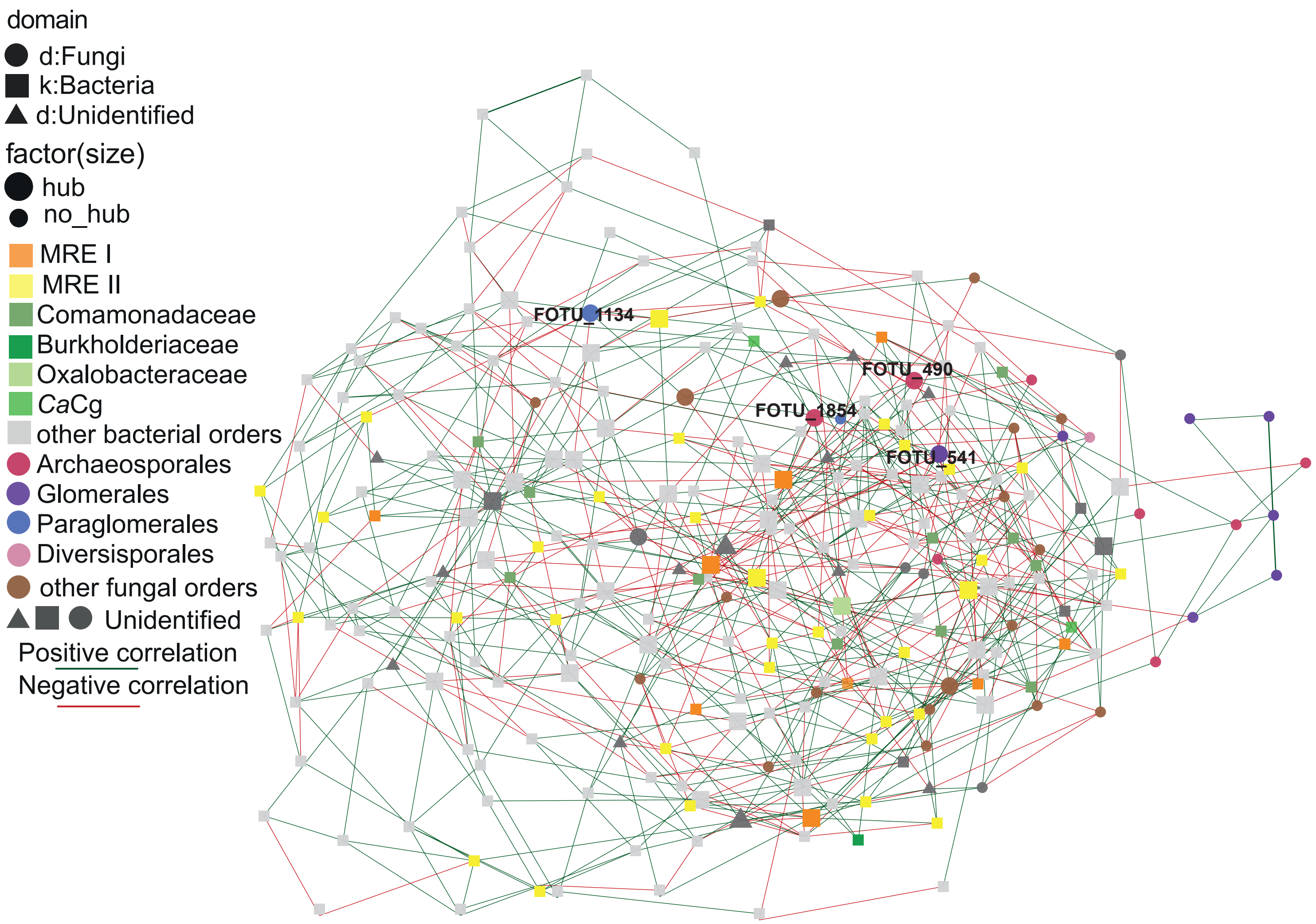

Figure S7. Co-occurrence network of spores. Fungal and bacterial OTUs from recovered spores. Vertices (OTUs) are colored based on the order, and edges (connections) based on their weight. Green and red lines were used for positive and negative correlations, respectively.

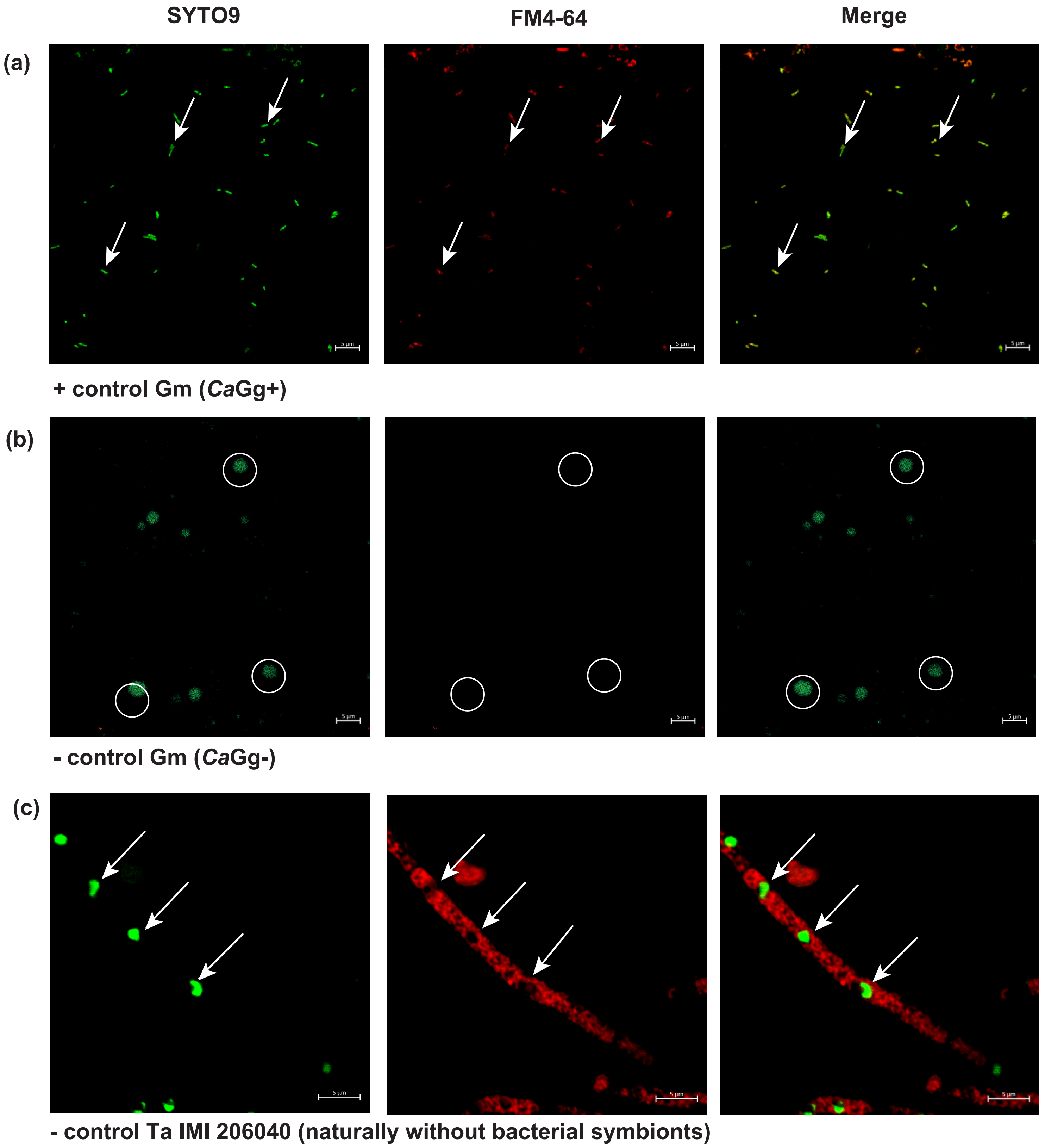

Figure S8. Multiphoton microscopy of control strains. (a) As positive control, the strain *Gigaspra margarita* BEG34+ was used. The presence of *Candidatus* Glomeribacter gigasporarum (*Ca*Gg), a rod-shaped bacterium, is shown and pointed out with arrows. (b) As one negative control, the strain *G. margarita* BEG34- was used. Green dots in the pictures showed the presence of fungal nuclei. Lack of red fluorescence (or signal) support the lack of bacteria (*Ca*Gg). (c) As the second negative control, the strain *Trichoderma atroviridae* IMI 206040 was used. Green dots in the pictures show the nuclei of fungi (stained by Syto9) and red dye represents the membrane of the hyphae. No bacterial cells live in this strain.

Table S1. PERMANOVA analysis of the AMF communities associated with agaves and cacti species considering all factors and their interactions (only factors are displayed; P ≤ 0.05) (Coleman-Derr *et al*., 2016; Fonseca-Garcia *et al*., 2016).

AMF OTUs			
factor	F	R^2	*p*
Season	0.702	0.00	0.753
Sample	2.349	0.04	**0.001**
Location	5.221	0.20	**0.001**
Species	2.533	0.03	**0.001**
Season:Sample	1.133	0.02	0.261
Season:Location	1.041	0.04	0.403
Sample:Location	1.429	0.13	**0.003**
Season:Species	1.108	0.01	0.320
Sample:Species	1.267	0.05	0.081
Location:Species	1.319	0.02	0.137
Season:Sample:Location	0.921	0.05	0.662
Season:Sample:Species	1.175	0.04	0.162
Season:Location:Species	0.9262	0.01	0.584
Sample:Location:Species	1.0759	0.04	0.345
Residual		0.29	
Total		1	

Table S2. Kruskal-Wallis/Dunn test of the average relative abundance of AMF total richness associated with agaves and cacti among the Locations investigated. No significant differences were found.

data: richness by Location
Kruskal-Wallis chi-squared = 5.8969, df = 4, p-value = 0.207

Comparison	Z	p. unadj	p .adj
Agave.Hill - Boyd.Ridge	-0.48	0.63	0.70
Agave.Hill - Magueyal	-0.82	0.41	0.59
Boyd.Ridge - Magueyal	-0.34	0.74	0.74
Agave.Hill - Pinyon.Flat	0.95	0.34	0.68
Boyd.Ridge - Pinyon.Flat	1.43	0.15	0.51
Magueyal - Pinyon.Flat	1.76	0.08	0.39
Agave.Hill - SanFelipe	-1.33	0.18	0.46
Boyd.Ridge - SanFelipe	-0.85	0.39	0.66
Magueyal - SanFelipe	-0.52	0.61	0.76
Pinyon.Flat - SanFelipe	-2.28	0.02	0.23

Table S3. Linear regression analysis among total richness and phosphorus concentration where native agaves and cacti are growing.

Residuals:				
SanFelipe	Magueyal	Boyd.Ridge	Pinyon.Flat	Agave.Hill
-2.726	-10.684	0.454	-15.098	28.054

Coefficients:				
	Estimate	Std. Error	t value	*P*
(Intercept)	149.402	33.895	4.408	0.0217
ric	-4.307	1.354	-3.182	0.05

Residual standard error: 19.47 on 3 degrees of freedom		
Multiple R-squared: 0.7714,	Adjusted R-squared: **0.70**	
F-statistic: 10.13 on 1 and 3 DF, p-value: 0.05001		

Table S4. Kruskal-Wallis/Dunn test of the root colonization by time and plant species.

Kruskal-Wallis rank sum test

data: root.colonization by time *A. tequilana*

Kruskal-Wallis chi-squared = 32.406, df = 2, p-value = 9.187e-08

Comparison	Z	P.unadj	P.adj
T4 - T8	-4.84038	0.0000013	**0.000004**
T4 - T12	5.224682	0.0000002	**0.000001**
T8 - T12	0.415094	0.6780731	1.000000

Kruskal-Wallis rank sum test

data: root.colonization by time *A. salmiana*

Kruskal-Wallis chi-squared = 33.431, df = 2, p-value = 5.502e-08

Comparison	Z	P.unadj	P.adj
T4 - T8	-4.518248	0.0000062	**0.0000187**
T4 - T12	5.542629	0.0000000	**0.0000001**
T8 - T12	1.106457	0.2685287	0.8055862

Kruskal-Wallis rank sum test

data: root.colonization by time *M. geometrizans*

Kruskal-Wallis chi-squared = 32.638, df = 2, p-value = 8.181e-08

Comparison	Z	P.unadj	P.adj
T4 - T8	-4.418073	0.00000996	**0.0000299**
T4 - T12	5.496958	0.00000004	**0.0000001**
T8 - T12	1.165329	0.24388580	0.7316575

Kruskal-Wallis rank sum test

data: root.colonization by plant.species T4

Kruskal-Wallis chi-squared = 26.762, df = 2, p-value = 1.544e-06

Comparison	Z	P.unadj	P.adj
Asal - Ateq	1.956956	0.050353	0.151058
Asal - Mgeo	5.125693	0.000000	**0.000001**
Ateq - Mgeo	3.168737	0.001531	**0.004593**

Kruskal-Wallis rank sum test

data: root.colonization by plant.species T8

Kruskal-Wallis chi-squared = 39.671, df = 2, p-value = 2.43e-09

Comparison	Z	P.unadj	P.adj
Asal - Ateq	-0.4714085	0.63734910	1
Asal - Mgeo	5.2036242	0.00000020	**0.00000059**
Ateq - Mgeo	5.6750326	0.00000001	**0.00000004**

Kruskal-Wallis rank sum test

data: root.colonization by plant.species T12

Kruskal-Wallis chi-squared = 39.506, df = 2, p-value = 2.638e-09

Comparison	Z	P.unadj	P.adj
Asal - Ateq	-0.1541809	0.8774671	1
Asal - Mgeo	5.3645882	0.0000001	**0.0000002**
Ateq - Mgeo	5.5187691	0.0000000	**0.0000001**

Table S5. Kruskal-Wallis/Dunn test of number of spores recovered from each plant species.

Anova test by time in *A. tequilana*
Residual standard error: 2.624669

	diff	lwr	upr	p adj
T4-T8	11	4.42459	17.575	**0.0052**
T4-T12	-17.667	-24.2421	-11.091	**0.0004**
T8-T12	-6.6667	-13.2421	-0.0913	**0.0475**

Anova test by time in *A. salmiana*
Residual standard error: 1.490712

	diff	lwr	upr	p adj
T4-T8	10.6667	6.9321	14.4013	**0.00030**
T4-T12	-19.667	-23.4013	-15.932	**0.00001**
T8-T12	-9.0000	-12.7346	-5.2654	**0.00077**

Anova test by Time *M. geometrizans*
Residual standard error: 2.160247

	diff	lwr	upr	p adj
T4-T8	7.3333	1.9214	12.7453	**0.0141**
T4-T12	-17.333	-22.7453	-11.921	**0.0002**
T8-T12	-10.000	-15.4119	-4.5881	**0.0031**

Anova test by plant species T4

Residual standard error: 1.247219

	diff	lwr	upr	p adj
Ateq-Asal	2.3333	-0.7912	5.4579	0.1332
Ateq-Mgeo	-4.0000	-7.1246	-0.8754	**0.0181**
Asal-Mgeo	-1.6667	-4.7912	1.4579	0.3024

Anova test by plant species T8

Residual standard error: 2.081666

	diff	lwr	upr	p adj
Ateq-Asal	2.667	-2.548	7.882	0.328
Ateq-Mgeo	-7.667	-12.882	-2.452	**0.010**
Asal-Mgeo	-5.000	-10.215	0.215	0.058

Anova test by plant species T12

Residual standard error: 2.808717

	diff	lwr	upr	p adj
Ateq-Asal	0.333	-6.703	7.370	0.988
Ateq-Mgeo	-4.333	-11.370	2.703	0.222
Asal-Mgeo	-4.000	-11.036	3.036	0.265

Table S6. Sequencing statistics from row data to the measurable and rarefied OTU tables.

AMPLICON	ITS2	16S-rRNA-V4
Quality trimming	TRIMMOMATIC default parameters	TRIMMOMATIC default parameters
Assemble	FLASH default parameters<br>-minoverlap 10<br>-max overlap 432	FLASH default parameters<br>-minoverlap 10<br>-max overlap 303
Filtering	VSEARCH<br>-stripright 18<br>-stripleft 39<br>-minlen 296<br>-maxlen 394<br>-expected error 1	VSEARCH<br>-stripright 24<br>-stripleft 23<br>-minlen 250<br>-maxlen 256<br>-expected error 1
Clustering	VSEARCH default parameters<br>similarity= 0.95	VSEARCH default parameters<br>similarity= 0.97
Chimera detection	VSEACH (uchime algorithm)<br>de novo	VSEACH (uchime algorithm)<br>de novo and reference base
Mapping	VSEARCH default parameters	VSEARCH default parameters
Classification	VSEARCH default parameters<br>-bootstrap 0.8<br><br>Bases<br>-Unite<br>-NCBI	VSEARCH default parameters<br>-bootstrap 0.8<br><br>Bases<br>-RDP
Total of input paired reads	2,291,463	3,824,642
Size range of reads	296-394	250 - 256 bp
Total of OTUs	23,049	33,220

Total of reads to map	2,262,791	3,779,209
Total of mapped reads	2,156,786	1,857,401
Total of Classified OTUs	2,750	19,553
Total OTUs after filtering	1533	1522

Table S7. List of the 61 AMF OTUs detected in this study.

domain	phylum	class	order	family	genus	otu.id
d:Fungi	p:Glomeromycota	c:Archaeosporomycetes	o:Archaeosporales	f:Ambisporaceae	g:Ambispora	OTU_87_Ambispora
d:Fungi	p:Glomeromycota	c:Archaeosporomycetes	o:Archaeosporales	f:Ambisporaceae	g:Ambispora	OTU_140_Ambispora
d:Fungi	p:Glomeromycota	c:Archaeosporomycetes	o:Archaeosporales	f:Ambisporaceae	g:Ambispora	OTU_195_Ambispora
d:Fungi	p:Glomeromycota	c:Archaeosporomycetes	o:Archaeosporales	f:Ambisporaceae	g:Ambispora	OTU_256_Ambispora
d:Fungi	p:Glomeromycota	c:Archaeosporomycetes	o:Archaeosporales	f:Ambisporaceae	g:Ambispora	OTU_490_Ambispora
d:Fungi	p:Glomeromycota	c:Glomeromycetes	o:Glomerales	f:Glomeraceae	g:Funneliformis	OTU_541_Funneliformis
d:Fungi	p:Glomeromycota	c:Archaeosporomycetes	o:Archaeosporales	f:Ambisporaceae	g:Ambispora	OTU_813_Ambispora
d:Fungi	p:Glomeromycota	c:Glomeromycetes	o:Glomerales	f:Glomeraceae	g:Kamienskia	OTU_844_Kamienskia
d:Fungi	p:Glomeromycota	c:Paraglomeromycetes	o:Paraglomerales	f:Paraglomeracea	g:Paraglomus	OTU_1134_Paraglomus
d:Fungi	p:Glomeromycota	c:Paraglomeromycetes	o:Paraglomerales	f:Paraglomeracea	g:Paraglomus	OTU_1278_Paraglomus
d:Fungi	p:Glomeromycota	c:Glomeromycetes	o:Glomerales	f:Glomeraceae	g:Rhizophagus	OTU_1339_Rhizophagus
d:Fungi	p:Glomeromycota	c:Archaeosporomycetes	o:Archaeosporales	f:Ambisporaceae	g:Ambispora	OTU_1854_Ambispora
d:Fungi	p:Glomeromycota	c:Glomeromycetes	o:Glomerales	f:Claroideoglomera	g:Claroideoglomus	OTU_2003_Claroideoglomus
d:Fungi	p:Glomeromycota	c:Glomeromycetes	o:Glomerales	f:Glomeraceae	g:Rhizophagus	OTU_2069_Rhizophagus
d:Fungi	p:Glomeromycota	c:Glomeromycetes	o:Glomerales	f:Glomeraceae	g:Glomus	OTU_2383_Glomus
d:Fungi	p:Glomeromycota	c:Glomeromycetes	o:Glomerales	f:Glomeraceae	g:Rhizophagus	OTU_2665_Rhizophagus
d:Fungi	p:Glomeromycota	c:Glomeromycetes	o:Glomerales	f:Glomeraceae	g:Rhizophagus	OTU_2726_Rhizophagus
d:Fungi	p:Glomeromycota	c:Glomeromycetes	o:Glomerales	f:Glomeraceae	g:Glomus	OTU_2797_Glomus
d:Fungi	p:Glomeromycota	c:Paraglomeromycetes	o:Paraglomerales	f:Pervetustaceae	g:Pervetustus	OTU_2872_Pervetustus
d:Fungi	p:Glomeromycota	c:Glomeromycetes	o:Glomerales	f:Glomeraceae	g:Glomus	OTU_2873_Glomus
d:Fungi	p:Glomeromycota	c:Glomeromycetes	o:Glomerales	f:Glomeraceae	g:Glomus	OTU_2874_Glomus
d:Fungi	p:Glomeromycota	c:Glomeromycetes	o:Glomerales	f:Glomeraceae	g:Glomus	OTU_2877_Glomus
d:Fungi	p:Glomeromycota	c:Paraglomeromycetes	o:Paraglomerales	f:Paraglomeracea	g:Paraglomus	OTU_2986_Paraglomus
d:Fungi	p:Glomeromycota	c:Archaeosporomycetes	o:Archaeosporales	f:Ambisporaceae	g:Ambispora	OTU_3092_Ambispora
d:Fungi	p:Glomeromycota	c:Glomeromycetes	o:Glomerales	f:Glomeraceae	g:Septoglomus	OTU_3189_Septoglomus
d:Fungi	p:Glomeromycota	c:Glomeromycetes	o:Glomerales	f:Glomeraceae	g:Glomus	OTU_3453_Glomus
d:Fungi	p:Glomeromycota	c:Glomeromycetes	o:Glomerales	f:Glomeraceae	g:Rhizoglomus	OTU_3995_Rhizoglomus
d:Fungi	p:Glomeromycota	c:Glomeromycetes	o:Glomerales	f:Glomeraceae	g:Glomus	OTU_4288_Glomus
d:Fungi	p:Glomeromycota	c:Glomeromycetes	o:Glomerales	f:Glomeraceae	g:Glomus	OTU_4548_Glomus
d:Fungi	p:Glomeromycota	c:Glomeromycetes	o:Diversisporales	f:Acaulosporacea	g:Acaulospora	OTU_4567_Acaulospora

d:Fungi	p:Glomeromycota	c:Glomeromycetes	o:Glomerales	f:Glomeraceae	g:Glomus	OTU_4854_Glomus
d:Fungi	p:Glomeromycota	c:Glomeromycetes	o:Glomerales	f:Glomeraceae	g:Funneliformis	OTU_4889_Funneliformis
d:Fungi	p:Glomeromycota	c:Glomeromycetes	o:Glomerales	f:Glomeraceae	g:Glomus	OTU_4899_Glomus
d:Fungi	p:Glomeromycota	c:Glomeromycetes	o:Glomerales	f:Glomeraceae	g:Rhizophagus	OTU_4902_Rhizophagus
d:Fungi	p:Glomeromycota	c:Glomeromycetes	o:Glomerales	f:Glomeraceae	g:Glomus	OTU_5326_Glomus
d:Fungi	p:Glomeromycota	c:Glomeromycetes	o:Glomerales	f:Glomeraceae	g:Kamienskia	OTU_5830_Kamienskia
d:Fungi	p:Glomeromycota	c:Glomeromycetes	o:Glomerales	f:Glomeraceae	g:Glomus	OTU_5940_Glomus
d:Fungi	p:Glomeromycota	c:Glomeromycetes	o:Glomerales	f:Glomeraceae	g:Glomus	OTU_5943_Glomus
d:Fungi	p:Glomeromycota	c:Glomeromycetes	o:Glomerales	f:Glomeraceae	g:Glomus	OTU_5952_Glomus
d:Fungi	p:Glomeromycota	c:Glomeromycetes	o:Glomerales	f:Glomeraceae	g:Glomus	OTU_5955_Glomus
d:Fungi	p:Glomeromycota	c:Glomeromycetes	o:Glomerales	f:Glomeraceae	g:Rhizophagus	OTU_6029_Rhizophagus
d:Fungi	p:Glomeromycota	c:Glomeromycetes	o:Glomerales	f:Glomeraceae	g:Rhizoglomus	OTU_6031_Rhizoglomus
d:Fungi	p:Glomeromycota	c:Glomeromycetes	o:Glomerales	f:Claroideoglomera	g:Claroideoglomus	OTU_6050_Claroideoglomus
d:Fungi	p:Glomeromycota	c:Archaeosporomycetes	o:Archaeosporales	f:Ambisporaceae	g:Ambispora	OTU_6068_Ambispora
d:Fungi	p:Glomeromycota	c:Glomeromycetes	o:Diversisporales	f:Acaulosporaceae	g:Acaulospora	OTU_6070_Acaulospora
d:Fungi	p:Glomeromycota	c:Glomeromycetes	o:Glomerales	f:Glomeraceae	g:Rhizoglomus	OTU_6998_Rhizoglomus
d:Fungi	p:Glomeromycota	c:Glomeromycetes	o:Glomerales	f:Glomeraceae	g:Glomus	OTU_7002_Glomus
d:Fungi	p:Glomeromycota	c:Glomeromycetes	o:Glomerales	f:Glomeraceae	g:Glomus	OTU_7004_Glomus
d:Fungi	p:Glomeromycota	c:Glomeromycetes	o:Glomerales	f:Glomeraceae	g:Rhizophagus	OTU_7027_Rhizophagus
d:Fungi	p:Glomeromycota	c:Glomeromycetes	o:Glomerales	f:Glomeraceae	g:Glomus	OTU_7037_Glomus
d:Fungi	p:Glomeromycota	c:Glomeromycetes	o:Glomerales	f:Glomeraceae	g:Glomus	OTU_7052_Glomus
d:Fungi	p:Glomeromycota	c:Glomeromycetes	o:Glomerales	f:Glomeraceae	g:Rhizophagus	OTU_7066_Rhizophagus
d:Fungi	p:Glomeromycota	c:Glomeromycetes	o:Glomerales	f:Glomeraceae	g:Glomus	OTU_7116_Glomus
d:Fungi	p:Glomeromycota	c:Glomeromycetes	o:Glomerales	f:Glomeraceae	g:Glomus	OTU_8586_Glomus
d:Fungi	p:Glomeromycota	c:Glomeromycetes	o:Glomerales	f:Glomeraceae	g:Glomus	OTU_8633_Glomus
d:Fungi	p:Glomeromycota	c:Glomeromycetes	o:Diversisporales	f:Diversisporales_	g:Entrophospora	OTU_8687_Entrophospora
d:Fungi	p:Glomeromycota	c:Paraglomeromycetes	o:Paraglomerales	f:Paraglomeracea	g:Paraglomus	OTU_8729_Paraglomus
d:Fungi	p:Glomeromycota	c:Glomeromycetes	o:Glomerales	f:Glomeraceae	g:Nanoglomus	OTU_8798_Nanoglomus
d:Fungi	p:Glomeromycota	c:Glomeromycetes	o:Glomerales	f:Glomeraceae	g:Rhizophagus	OTU_8803_Rhizophagus
d:Fungi	p:Glomeromycota	c:Paraglomeromycetes	o:Paraglomerales	f:Paraglomeracea	g:Paraglomus	OTU_8804_Paraglomus
d:Fungi	p:Glomeromycota	c:Glomeromycetes	o:Glomerales	f:Glomeraceae	g:Rhizophagus	OTU_8848_Rhizophagus

Table S8. List of the 550 spores-associated bacterial OTUs detected in this study.

domain	phylum	class	order	family	genus	otu.id
k:Bacteria	p:Actinobacteria	c:Acidimicrobiia	o:Acidimicrobiales	f:OM1_clade	NA	OTU_1245
k:Bacteria	p:Actinobacteria	c:Acidimicrobiia	o:Acidimicrobiales	NA	NA	OTU_1492
k:Bacteria	p:Actinobacteria	c:Acidimicrobiia	o:Acidimicrobiales	NA	NA	OTU_1833
k:Bacteria	p:Actinobacteria	c:Acidimicrobiia	o:Acidimicrobiales	NA	NA	OTU_375
k:Bacteria	p:Actinobacteria	c:Acidimicrobiia	o:Acidimicrobiales	NA	NA	OTU_379
k:Bacteria	p:Actinobacteria	c:Acidimicrobiia	o:Acidimicrobiales	f:Iamiaceae	g:Iamia	OTU_434
k:Bacteria	p:Actinobacteria	c:Acidimicrobiia	o:Acidimicrobiales	NA	NA	OTU_659
k:Bacteria	p:Actinobacteria	c:Acidimicrobiia	o:Acidimicrobiales	NA	NA	OTU_719
k:Bacteria	p:Actinobacteria	c:Acidimicrobiia	o:Acidimicrobiales	NA	NA	OTU_762
k:Bacteria	p:Actinobacteria	c:Acidimicrobiia	o:Acidimicrobiales	NA	NA	OTU_785
k:Bacteria	p:Actinobacteria	c:Acidimicrobiia	o:Acidimicrobiales	NA	NA	OTU_815
k:Bacteria	p:Actinobacteria	c:Acidimicrobiia	o:Acidimicrobiales	f:Acidimicrobiales_Incertae_	g:Ca_Microthrix	OTU_909
k:Bacteria	p:Actinobacteria	c:Actinobacteria	o:Actinomycetales	f:Actinomycetaceae	g:Actinomyces	OTU_565
k:Bacteria	p:Proteobacteria	c:Gammaproteobacteria	o:Aeromonadales	f:Aeromonadaceae	g:Aeromonas	OTU_88
k:Bacteria	p:Chloroflexi	c:Anaerolineae	o:Anaerolineales	f:Anaerolineaceae	NA	OTU_1593
k:Bacteria	p:Chloroflexi	c:Anaerolineae	o:Anaerolineales	f:Anaerolineaceae	NA	OTU_3274
k:Bacteria	p:Chloroflexi	c:Anaerolineae	o:Anaerolineales	f:Anaerolineaceae	NA	OTU_509
k:Bacteria	p:Chloroflexi	c:Anaerolineae	o:Anaerolineales	f:Anaerolineaceae	NA	OTU_935
k:Bacteria	p:Gemmatimonadetes	c:Gemmatimonadetes	o:AT425-EubC11_terre	NA	NA	OTU_169
k:Bacteria	p:Gemmatimonadetes	c:Gemmatimonadetes	o:AT425-EubC11_terre	NA	NA	OTU_1698
k:Bacteria	p:Gemmatimonadetes	c:Gemmatimonadetes	o:AT425-EubC11_terre	NA	NA	OTU_2395
k:Bacteria	p:Tenericutes	c:Bacilli	o:Bacillales	f:Alicyclobacillaceae	g:Tumebacillus	OTU_10172
k:Bacteria	p:Tenericutes	c:Bacilli	o:Bacillales	f:Paenibacillaceae	g:Paenibacillus	OTU_1048
k:Bacteria	p:Tenericutes	c:Bacilli	o:Bacillales	NA	NA	OTU_11264
k:Bacteria	p:Tenericutes	c:Bacilli	o:Bacillales	f:Alicyclobacillaceae	g:Tumebacillus	OTU_1175
k:Bacteria	p:Tenericutes	c:Bacilli	o:Bacillales	f:Planococcaceae	g:Chryseomicrobium	OTU_1383
k:Bacteria	p:Tenericutes	c:Bacilli	o:Bacillales	f:Bacillaceae	g:Bacillus	OTU_14
k:Bacteria	p:Tenericutes	c:Bacilli	o:Bacillales	f:Bacillaceae	g:Bacillus	OTU_1601
k:Bacteria	p:Tenericutes	c:Bacilli	o:Bacillales	f:Bacillaceae	g:Bacillus	OTU_163
k:Bacteria	p:Tenericutes	c:Bacilli	o:Bacillales	f:Planococcaceae	g:Rummeliibacillus	OTU_1663
k:Bacteria	p:Tenericutes	c:Bacilli	o:Bacillales	f:Paenibacillaceae	g:Paenibacillus	OTU_1738
k:Bacteria	p:Tenericutes	c:Bacilli	o:Bacillales	f:Thermoactinomycetaceae	g:Shimazuella	OTU_1807

k:Bacteria p:Tenericutes	c:Bacilli	o:Bacillales	f:Alicyclobacillaceae	g:Tumebacillus	OTU_18363
k:Bacteria p:Tenericutes	c:Bacilli	o:Bacillales	f:Paenibacillaceae	g:Paenibacillus	OTU_2221
k:Bacteria p:Tenericutes	c:Bacilli	o:Bacillales	f:Bacillaceae	g:Bacillus	OTU_2304
k:Bacteria p:Tenericutes	c:Bacilli	o:Bacillales	f:Paenibacillaceae	NA	OTU_233
k:Bacteria p:Tenericutes	c:Bacilli	o:Bacillales	f:Bacillaceae	NA	OTU_2764
k:Bacteria p:Tenericutes	c:Bacilli	o:Bacillales	f:Bacillaceae	g:Bacillus	OTU_2944
k:Bacteria p:Tenericutes	c:Bacilli	o:Bacillales	f:Family_XI	g:Gemella	OTU_298
k:Bacteria p:Tenericutes	c:Bacilli	o:Bacillales	f:Bacillaceae	g:Bacillus	OTU_31
k:Bacteria p:Tenericutes	c:Bacilli	o:Bacillales	f:Alicyclobacillaceae	g:Tumebacillus	OTU_311
k:Bacteria p:Tenericutes	c:Bacilli	o:Bacillales	f:Bacillaceae	g:Bacillus	OTU_3399
k:Bacteria p:Tenericutes	c:Bacilli	o:Bacillales	f:Bacillaceae	g:Bacillus	OTU_34
k:Bacteria p:Tenericutes	c:Bacilli	o:Bacillales	f:Bacillaceae	g:Bacillus	OTU_347
k:Bacteria p:Tenericutes	c:Bacilli	o:Bacillales	f:Alicyclobacillaceae	g:Tumebacillus	OTU_3524
k:Bacteria p:Tenericutes	c:Bacilli	o:Bacillales	f:Bacillaceae	g:Bacillus	OTU_401
k:Bacteria p:Tenericutes	c:Bacilli	o:Bacillales	f:Alicyclobacillaceae	g:Tumebacillus	OTU_414
k:Bacteria p:Tenericutes	c:Bacilli	o:Bacillales	f:Bacillaceae	g:Bacillus	OTU_4758
k:Bacteria p:Tenericutes	c:Bacilli	o:Bacillales	f:Planococcaceae	g:Lysinibacillus	OTU_527
k:Bacteria p:Tenericutes	c:Bacilli	o:Bacillales	f:Bacillaceae	g:Bacillus	OTU_553
k:Bacteria p:Tenericutes	c:Bacilli	o:Bacillales	f:Bacillaceae	NA	OTU_559
k:Bacteria p:Tenericutes	c:Bacilli	o:Bacillales	f:Bacillaceae	g:Bacillus	OTU_579
k:Bacteria p:Tenericutes	c:Bacilli	o:Bacillales	f:Family_XII	g:Exiguobacterium	OTU_657
k:Bacteria p:Tenericutes	c:Bacilli	o:Bacillales	f:Paenibacillaceae	g:Cohnella	OTU_779
k:Bacteria p:Tenericutes	c:Bacilli	o:Bacillales	f:Bacillaceae	g:Bacillus	OTU_80
k:Bacteria p:Tenericutes	c:Bacilli	o:Bacillales	f:Staphylococcaceae	g:Staphylococcus	OTU_87
k:Bacteria p:Tenericutes	c:Bacilli	o:Bacillales	f:Bacillaceae	g:Bacillus	OTU_880
k:Bacteria p:Tenericutes	c:Bacilli	o:Bacillales	f:Bacillaceae	g:Bacillus	OTU_927
k:Bacteria p:Tenericutes	c:Bacilli	o:Bacillales	f:Bacillaceae	g:Bacillus	OTU_929
k:Bacteria p:Tenericutes	c:Bacilli	o:Bacillales	f:Bacillaceae	g:Bacillus	OTU_964
k:Bacteria p:Bacteroidetes	c:Bacteroidia	o:Bacteroidales	f:Prevotellaceae	g:Alloprevotella	OTU_2220
k:Bacteria p:Bacteroidetes	c:Bacteroidia	o:Bacteroidales	f:Porphyromonadaceae	g:Porphyromonas	OTU_511
k:Bacteria p:Bacteroidetes	c:Bacteroidia	o:Bacteroidales	f:Prevotellaceae	g:Prevotella	OTU_664
k:Bacteria p:Bacteroidetes	c:Bacteroidia	o:Bacteroidales	f:Prevotellaceae	g:Alloprevotella	OTU_686
k:Bacteria p:Proteobacteria	c:Betaproteobacteria	o:Burkholderiales	f:Comamonadaceae	NA	OTU_10543
k:Bacteria p:Proteobacteria	c:Betaproteobacteria	o:Burkholderiales	f:Comamonadaceae	NA	OTU_1092
k:Bacteria p:Proteobacteria	c:Betaproteobacteria	o:Burkholderiales	f:Comamonadaceae	NA	OTU_116

k:Bacteria	p:Proteobacteria	c:Betaproteobacteria	o:Burkholderiales	f:Oxalobacteraceae	g:Massilia	OTU_15
k:Bacteria	p:Proteobacteria	c:Betaproteobacteria	o:Burkholderiales	f:Comamonadaceae	NA	OTU_201
k:Bacteria	p:Proteobacteria	c:Betaproteobacteria	o:Burkholderiales	f:Burkholderiaceae	g:Limnobacter	OTU_204
k:Bacteria	p:Proteobacteria	c:Betaproteobacteria	o:Burkholderiales	f:Comamonadaceae	g:Schlegelella	OTU_2313
k:Bacteria	p:Proteobacteria	c:Betaproteobacteria	o:Burkholderiales	f:Comamonadaceae	NA	OTU_24
k:Bacteria	p:Proteobacteria	c:Betaproteobacteria	o:Burkholderiales	f:Burkholderiaceae	g:Ca_Glomeribacter	OTU_2447
k:Bacteria	p:Proteobacteria	c:Betaproteobacteria	o:Burkholderiales	f:Comamonadaceae	NA	OTU_3451
k:Bacteria	p:Proteobacteria	c:Betaproteobacteria	o:Burkholderiales	f:Oxalobacteraceae	g:Noviherbaspirillum	OTU_36
k:Bacteria	p:Proteobacteria	c:Betaproteobacteria	o:Burkholderiales	f:Comamonadaceae	NA	OTU_3674
k:Bacteria	p:Proteobacteria	c:Betaproteobacteria	o:Burkholderiales	f:Comamonadaceae	NA	OTU_461
k:Bacteria	p:Proteobacteria	c:Betaproteobacteria	o:Burkholderiales	f:Oxalobacteraceae	g:Massilia	OTU_481
k:Bacteria	p:Proteobacteria	c:Betaproteobacteria	o:Burkholderiales	f:Burkholderiaceae	g:Ralstonia	OTU_516
k:Bacteria	p:Proteobacteria	c:Betaproteobacteria	o:Burkholderiales	f:Comamonadaceae	NA	OTU_544
k:Bacteria	p:Proteobacteria	c:Betaproteobacteria	o:Burkholderiales	f:Burkholderiaceae	g:Ca_Glomeribacter	OTU_568
k:Bacteria	p:Proteobacteria	c:Betaproteobacteria	o:Burkholderiales	f:Comamonadaceae	NA	OTU_6198
k:Bacteria	p:Proteobacteria	c:Betaproteobacteria	o:Burkholderiales	f:Comamonadaceae	NA	OTU_6345
k:Bacteria	p:Proteobacteria	c:Betaproteobacteria	o:Burkholderiales	f:Comamonadaceae	NA	OTU_7468
k:Bacteria	p:Proteobacteria	c:Betaproteobacteria	o:Burkholderiales	f:Burkholderiaceae	g:Burkholderia	OTU_79
k:Bacteria	p:Proteobacteria	c:Betaproteobacteria	o:Burkholderiales	f:Burkholderiaceae	g:Burkholderia	OTU_82
k:Bacteria	p:Proteobacteria	c:Betaproteobacteria	o:Burkholderiales	f:Comamonadaceae	NA	OTU_939
k:Bacteria	p:Proteobacteria	c:Epsilonproteobacteria	o:Campylobacterales	f:Campylobacteraceae	g:Campylobacter	OTU_869
k:Bacteria	p:Actinobacteria	c:Actinobacteria	o:Catenulisporales	f:Actinospicaceae	g:Actinospica	OTU_1057
k:Bacteria	p:Actinobacteria	c:Actinobacteria	o:Catenulisporales	f:Actinospicaceae	g:Actinospica	OTU_404
k:Bacteria	p:Proteobacteria	c:Alphaproteobacteria	o:Caulobacterales	f:Caulobacteraceae	g:Brevundimonas	OTU_134
k:Bacteria	p:Proteobacteria	c:Alphaproteobacteria	o:Caulobacterales	f:Caulobacteraceae	NA	OTU_182
k:Bacteria	p:Proteobacteria	c:Alphaproteobacteria	o:Caulobacterales	f:Caulobacteraceae	g:Phenylobacterium	OTU_197
k:Bacteria	p:Proteobacteria	c:Alphaproteobacteria	o:Caulobacterales	f:Caulobacteraceae	NA	OTU_2420
k:Bacteria	p:Proteobacteria	c:Alphaproteobacteria	o:Caulobacterales	f:Caulobacteraceae	g:Brevundimonas	OTU_3671
k:Bacteria	p:Proteobacteria	c:Alphaproteobacteria	o:Caulobacterales	f:Caulobacteraceae	g:Brevundimonas	OTU_90
k:Bacteria	p:Chloroflexi	c:Chloroflexia	o:Chloroflexales	f:Roseiflexaceae	g:Roseiflexus	OTU_1274
k:Bacteria	p:Proteobacteria	c:Gammaproteobacteria	o:Chromatiales	f:Ectothiorhodospiraceae	g:Acidiferrobacter	OTU_1584
k:Bacteria	p:Proteobacteria	c:Gammaproteobacteria	o:Chromatiales	f:Chromatiaceae	g:Rheinheimera	OTU_981
k:Bacteria	p:Verrucomicrobia	c:Spartobacteria	o:Chthoniobacterales	f:DA101_soil_group	NA	OTU_809
k:Bacteria	p:Tenericutes	c:Clostridia	o:Clostridiales	f:Family_XI	g:Anaerococcus	OTU_1528
k:Bacteria	p:Tenericutes	c:Clostridia	o:Clostridiales	f:Family_XI	g:Anaerococcus	OTU_2379

k:Bacteria	p:Tenericutes	c:Clostridia	o:Clostridiales	f:Family_XI	g:Peptoniphilus	OTU_419
k:Bacteria	p:Tenericutes	c:Clostridia	o:Clostridiales	f:Family_XI	g:Finegoldia	OTU_628
k:Bacteria	p:Tenericutes	c:Clostridia	o:Clostridiales	f:Lachnospiraceae	g:Lachnoanaerobaculu	OTU_960
k:Bacteria	p:Actinobacteria	c:Actinobacteria	o:Corynebacteriales	f:Nocardiaceae	g:Nocardia	OTU_1005
k:Bacteria	p:Actinobacteria	c:Actinobacteria	o:Corynebacteriales	f:Mycobacteriaceae	g:Mycobacterium	OTU_1097
k:Bacteria	p:Actinobacteria	c:Actinobacteria	o:Corynebacteriales	f:Corynebacteriaceae	g:Corynebacterium	OTU_1194
k:Bacteria	p:Actinobacteria	c:Actinobacteria	o:Corynebacteriales	f:Corynebacteriaceae	g:Corynebacterium_1	OTU_128
k:Bacteria	p:Actinobacteria	c:Actinobacteria	o:Corynebacteriales	f:Corynebacteriaceae	g:Corynebacterium	OTU_2556
k:Bacteria	p:Actinobacteria	c:Actinobacteria	o:Corynebacteriales	f:Corynebacteriaceae	g:Corynebacterium_1	OTU_268
k:Bacteria	p:Actinobacteria	c:Actinobacteria	o:Corynebacteriales	f:Mycobacteriaceae	g:Mycobacterium	OTU_278
k:Bacteria	p:Actinobacteria	c:Actinobacteria	o:Corynebacteriales	f:Mycobacteriaceae	g:Mycobacterium	OTU_279
k:Bacteria	p:Actinobacteria	c:Actinobacteria	o:Corynebacteriales	f:Dietziaceae	g:Dietzia	OTU_282
k:Bacteria	p:Actinobacteria	c:Actinobacteria	o:Corynebacteriales	f:Corynebacteriaceae	NA	OTU_309
k:Bacteria	p:Actinobacteria	c:Actinobacteria	o:Corynebacteriales	f:Nocardiaceae	g:Rhodococcus	OTU_371
k:Bacteria	p:Actinobacteria	c:Actinobacteria	o:Corynebacteriales	f:Corynebacteriaceae	g:Corynebacterium	OTU_412
k:Bacteria	p:Actinobacteria	c:Actinobacteria	o:Corynebacteriales	f:Mycobacteriaceae	g:Mycobacterium	OTU_485
k:Bacteria	p:Bacteroidetes	c:Cytophagia	o:Cytophagales	f:Cytophagaceae	NA	OTU_1009
k:Bacteria	p:Bacteroidetes	c:Cytophagia	o:Cytophagales	f:Cytophagaceae	NA	OTU_1108
k:Bacteria	p:Bacteroidetes	c:Cytophagia	o:Cytophagales	f:Cytophagaceae	NA	OTU_1234
k:Bacteria	p:Bacteroidetes	c:Cytophagia	o:Cytophagales	f:Cytophagaceae	NA	OTU_1567
k:Bacteria	p:Bacteroidetes	c:Cytophagia	o:Cytophagales	f:Cytophagaceae	NA	OTU_1926
k:Bacteria	p:Bacteroidetes	c:Cytophagia	o:Cytophagales	f:Cytophagaceae	g:Cytophaga	OTU_2642
k:Bacteria	p:Bacteroidetes	c:Cytophagia	o:Cytophagales	f:Cytophagaceae	NA	OTU_3445
k:Bacteria	p:Bacteroidetes	c:Cytophagia	o:Cytophagales	f:Cytophagaceae	NA	OTU_3801
k:Bacteria	p:Bacteroidetes	c:Cytophagia	o:Cytophagales	f:Cytophagaceae	g:Ohtaekwangia	OTU_405
k:Bacteria	p:Bacteroidetes	c:Cytophagia	o:Cytophagales	f:Cytophagaceae	g:Rhodocytophaga	OTU_518
k:Bacteria	p:Bacteroidetes	c:Cytophagia	o:Cytophagales	f:Cytophagaceae	NA	OTU_618
k:Bacteria	p:Bacteroidetes	c:Cytophagia	o:Cytophagales	f:Cytophagaceae	g:Ohtaekwangia	OTU_64
k:Bacteria	p:Bacteroidetes	c:Cytophagia	o:Cytophagales	f:Cytophagaceae	NA	OTU_715
k:Bacteria	p:Bacteroidetes	c:Cytophagia	o:Cytophagales	f:Cytophagaceae	g:Pontibacter	OTU_877
k:Bacteria	p:Bacteroidetes	c:Cytophagia	o:Cytophagales	f:Cytophagaceae	NA	OTU_898
k:Bacteria	p:Bacteroidetes	c:Cytophagia	o:Cytophagales	f:Flammeovirgaceae	g:Cesiribacter	OTU_967
k:Bacteria	p:Deinococcus-Thermus	c:Deinococci	o:Deinococcales	f:Trueperaceae	g:Truepera	OTU_665
k:Bacteria	p:Proteobacteria	c:Gammaproteobacteria	o:Enterobacteriales	f:Enterobacteriaceae	g:Pantoea	OTU_3
k:Bacteria	p:Proteobacteria	c:Gammaproteobacteria	o:Enterobacteriales	f:Enterobacteriaceae	NA	OTU_91

k:Bacteria p:Bacteroidetes	c:Flavobacteriia	o:Flavobacteriales	f:NS9_marine_group	NA	OTU_1036
k:Bacteria p:Bacteroidetes	c:Flavobacteriia	o:Flavobacteriales	f:Flavobacteriaceae	g:Chryseobacterium	OTU_1953
k:Bacteria p:Bacteroidetes	c:Flavobacteriia	o:Flavobacteriales	f:Flavobacteriaceae	g:Flavobacterium	OTU_38
k:Bacteria p:Bacteroidetes	c:Flavobacteriia	o:Flavobacteriales	f:Flavobacteriaceae	g:Flavobacterium	OTU_609
k:Bacteria p:Actinobacteria	c:Actinobacteria	o:Frankiales	f:Geodermatophilaceae	NA	OTU_1044
k:Bacteria p:Actinobacteria	c:Actinobacteria	o:Frankiales	f:Geodermatophilaceae	g:Geodermatophilus	OTU_1267
k:Bacteria p:Actinobacteria	c:Actinobacteria	o:Frankiales	f:Acidothermaceae	g:Acidothermus	OTU_170
k:Bacteria p:Actinobacteria	c:Actinobacteria	o:Frankiales	f:Nakamurellaceae	g:Nakamurella	OTU_2026
k:Bacteria p:Actinobacteria	c:Actinobacteria	o:Frankiales	f:Geodermatophilaceae	g:Modestobacter	OTU_28
k:Bacteria p:Actinobacteria	c:Actinobacteria	o:Frankiales	f:Acidothermaceae	g:Acidothermus	OTU_3408
k:Bacteria p:Actinobacteria	c:Actinobacteria	o:Frankiales	f:Acidothermaceae	g:Acidothermus	OTU_450
k:Bacteria p:Actinobacteria	c:Actinobacteria	o:Frankiales	f:Geodermatophilaceae	g:Geodermatophilus	OTU_5451
k:Bacteria p:Actinobacteria	c:Actinobacteria	o:Frankiales	f:Cryptosporangiaceae	g:Cryptosporangium	OTU_826
k:Bacteria p:Fusobacteria	c:Fusobacteriia	o:Fusobacteriales	f:Leptotrichiaceae	g:Leptotrichia	OTU_177
k:Bacteria p:Fusobacteria	c:Fusobacteriia	o:Fusobacteriales	f:Leptotrichiaceae	g:Leptotrichia	OTU_3373
k:Bacteria p:Fusobacteria	c:Fusobacteriia	o:Fusobacteriales	f:Fusobacteriaceae	g:Fusobacterium	OTU_410
k:Bacteria p:Fusobacteria	c:Fusobacteriia	o:Fusobacteriales	f:Leptotrichiaceae	g:Leptotrichia	OTU_467
k:Bacteria p:Actinobacteria	c:Thermoleophilia	o:Gaiellales	NA	NA	OTU_1404
k:Bacteria p:Actinobacteria	c:Thermoleophilia	o:Gaiellales	f:Gaiellaceae	g:Gaiella	OTU_2128
k:Bacteria p:Actinobacteria	c:Thermoleophilia	o:Gaiellales	f:Gaiellaceae	g:Gaiella	OTU_2422
k:Bacteria p:Actinobacteria	c:Thermoleophilia	o:Gaiellales	NA	NA	OTU_682
k:Bacteria p:Gemmatimonadetes	c:Gemmatimonadetes	o:Gemmatimonadales	f:Gemmatimonadaceae	NA	OTU_1150
k:Bacteria p:Gemmatimonadetes	c:Gemmatimonadetes	o:Gemmatimonadales	f:Gemmatimonadaceae	g:Gemmatimonas	OTU_750
k:Bacteria p:Proteobacteria	c:Deltaproteobacteria	o:GR-WP33-30	NA	NA	OTU_1966
k:Bacteria p:Proteobacteria	c:Deltaproteobacteria	o:GR-WP33-30	NA	NA	OTU_642
k:Bacteria p:Chloroflexi	c:Thermomicrobia	o:JG30-KF-CM45	NA	NA	OTU_1773
k:Bacteria p:Chloroflexi	c:Thermomicrobia	o:JG30-KF-CM45	NA	NA	OTU_262
k:Bacteria p:Chloroflexi	c:Thermomicrobia	o:JG30-KF-CM45	NA	NA	OTU_718
k:Bacteria p:Actinobacteria	c:Actinobacteria	o:Kineosporiales	f:Kineosporiaceae	NA	OTU_49
k:Bacteria p:Chloroflexi	c:Ktedonobacteria	o:Ktedonobacterales	NA	NA	OTU_1890
k:Bacteria p:Chloroflexi	c:Ktedonobacteria	o:Ktedonobacterales	f:Thermosporotrichaceae	g:Thermosporothrix	OTU_2082
k:Bacteria p:Chloroflexi	c:Ktedonobacteria	o:Ktedonobacterales	f:Ktedonobacteraceae	NA	OTU_225
k:Bacteria p:Chloroflexi	c:Ktedonobacteria	o:Ktedonobacterales	f:Ktedonobacteraceae	NA	OTU_473
k:Bacteria p:Tenericutes	c:Bacilli	o:Lactobacillales	NA	NA	OTU_21098
k:Bacteria p:Tenericutes	c:Bacilli	o:Lactobacillales	f:Streptococcaceae	g:Streptococcus	OTU_215

k:Bacteria	p:Tenericutes	c:Bacilli	o:Lactobacillales	f:Carnobacteriaceae	g:Granulicatella	OTU_328
k:Bacteria	p:Tenericutes	c:Bacilli	o:Lactobacillales	f:Streptococcaceae	g:Streptococcus	OTU_3897
k:Bacteria	p:Tenericutes	c:Bacilli	o:Lactobacillales	NA	NA	OTU_636
k:Bacteria	p:Actinobacteria	c:Actinobacteria	o:Micrococcales	f:Micrococcaceae	g:Rothia	OTU_1000
k:Bacteria	p:Actinobacteria	c:Actinobacteria	o:Micrococcales	f:Microbacteriaceae	NA	OTU_1157
k:Bacteria	p:Actinobacteria	c:Actinobacteria	o:Micrococcales	f:Micrococcaceae	g:Arthrobacter	OTU_13
k:Bacteria	p:Actinobacteria	c:Actinobacteria	o:Micrococcales	f:Microbacteriaceae	NA	OTU_222
k:Bacteria	p:Actinobacteria	c:Actinobacteria	o:Micrococcales	f:Intrasporangiaceae	NA	OTU_229
k:Bacteria	p:Actinobacteria	c:Actinobacteria	o:Micrococcales	f:Microbacteriaceae	NA	OTU_284
k:Bacteria	p:Actinobacteria	c:Actinobacteria	o:Micrococcales	f:Micrococcaceae	g:Kocuria	OTU_48
k:Bacteria	p:Actinobacteria	c:Actinobacteria	o:Micrococcales	f:Microbacteriaceae	g:Microbacterium	OTU_4880
k:Bacteria	p:Actinobacteria	c:Actinobacteria	o:Micrococcales	f:Micrococcaceae	g:Arthrobacter	OTU_496
k:Bacteria	p:Actinobacteria	c:Actinobacteria	o:Micrococcales	f:Micrococcaceae	g:Arthrobacter	OTU_523
k:Bacteria	p:Actinobacteria	c:Actinobacteria	o:Micrococcales	f:Micrococcaceae	g:Rothia	OTU_564
k:Bacteria	p:Actinobacteria	c:Actinobacteria	o:Micrococcales	f:Microbacteriaceae	NA	OTU_6
k:Bacteria	p:Actinobacteria	c:Actinobacteria	o:Micrococcales	f:Cellulomonadaceae	NA	OTU_68
k:Bacteria	p:Actinobacteria	c:Actinobacteria	o:Micrococcales	NA	NA	OTU_99
k:Bacteria	p:Actinobacteria	c:Actinobacteria	o:Micromonosporales	f:Micromonosporaceae	NA	OTU_1137
k:Bacteria	p:Actinobacteria	c:Actinobacteria	o:Micromonosporales	f:Micromonosporaceae	g:Dactylosporangium	OTU_1140
k:Bacteria	p:Actinobacteria	c:Actinobacteria	o:Micromonosporales	f:Micromonosporaceae	NA	OTU_1176
k:Bacteria	p:Actinobacteria	c:Actinobacteria	o:Micromonosporales	f:Micromonosporaceae	NA	OTU_1197
k:Bacteria	p:Actinobacteria	c:Actinobacteria	o:Micromonosporales	f:Micromonosporaceae	NA	OTU_1277
k:Bacteria	p:Actinobacteria	c:Actinobacteria	o:Micromonosporales	f:Micromonosporaceae	g:Planosporangium	OTU_1331
k:Bacteria	p:Actinobacteria	c:Actinobacteria	o:Micromonosporales	f:Micromonosporaceae	NA	OTU_1366
k:Bacteria	p:Actinobacteria	c:Actinobacteria	o:Micromonosporales	f:Micromonosporaceae	NA	OTU_1754
k:Bacteria	p:Actinobacteria	c:Actinobacteria	o:Micromonosporales	f:Micromonosporaceae	g:Actinoplanes	OTU_179
k:Bacteria	p:Actinobacteria	c:Actinobacteria	o:Micromonosporales	f:Micromonosporaceae	NA	OTU_1798
k:Bacteria	p:Actinobacteria	c:Actinobacteria	o:Micromonosporales	f:Micromonosporaceae	NA	OTU_199
k:Bacteria	p:Actinobacteria	c:Actinobacteria	o:Micromonosporales	f:Micromonosporaceae	NA	OTU_2159
k:Bacteria	p:Actinobacteria	c:Actinobacteria	o:Micromonosporales	f:Micromonosporaceae	NA	OTU_2242
k:Bacteria	p:Actinobacteria	c:Actinobacteria	o:Micromonosporales	f:Micromonosporaceae	g:Actinoplanes	OTU_247
k:Bacteria	p:Actinobacteria	c:Actinobacteria	o:Micromonosporales	f:Micromonosporaceae	NA	OTU_280
k:Bacteria	p:Actinobacteria	c:Actinobacteria	o:Micromonosporales	f:Micromonosporaceae	NA	OTU_312
k:Bacteria	p:Actinobacteria	c:Actinobacteria	o:Micromonosporales	f:Micromonosporaceae	NA	OTU_344
k:Bacteria	p:Actinobacteria	c:Actinobacteria	o:Micromonosporales	f:Micromonosporaceae	g:Dactylosporangium	OTU_365

k:Bacteria p:Actinobacteria	c:Actinobacteria	o:Micromonosporales	f:Micromonosporaceae	g:Actinoplanes	OTU_388
k:Bacteria p:Actinobacteria	c:Actinobacteria	o:Micromonosporales	f:Micromonosporaceae	NA	OTU_444
k:Bacteria p:Actinobacteria	c:Actinobacteria	o:Micromonosporales	f:Micromonosporaceae	NA	OTU_5
k:Bacteria p:Actinobacteria	c:Actinobacteria	o:Micromonosporales	f:Micromonosporaceae	NA	OTU_5069
k:Bacteria p:Actinobacteria	c:Actinobacteria	o:Micromonosporales	f:Micromonosporaceae	NA	OTU_5445
k:Bacteria p:Actinobacteria	c:Actinobacteria	o:Micromonosporales	f:Micromonosporaceae	NA	OTU_5725
k:Bacteria p:Actinobacteria	c:Actinobacteria	o:Micromonosporales	f:Micromonosporaceae	NA	OTU_66
k:Bacteria p:Actinobacteria	c:Actinobacteria	o:Micromonosporales	f:Micromonosporaceae	NA	OTU_7029
k:Bacteria p:Actinobacteria	c:Actinobacteria	o:Micromonosporales	f:Micromonosporaceae	NA	OTU_723
k:Bacteria p:Actinobacteria	c:Actinobacteria	o:Micromonosporales	f:Micromonosporaceae	g:Rugosimonospora	OTU_895
k:Bacteria p:Actinobacteria	c:Actinobacteria	o:Micromonosporales	f:Micromonosporaceae	g:Actinoplanes	OTU_8968
k:Bacteria p:Tenericutes	c:Mollicutes	o:Mycoplasmatales	f:Mycoplasmataceae	g:Ca_Moeniiplasma	OTU_1023
k:Bacteria p:Tenericutes	c:Mollicutes	o:Mycoplasmatales	f:Mycoplasmataceae	g:Ca_Moeniiplasma	OTU_108
k:Bacteria p:Tenericutes	c:Mollicutes	o:Mycoplasmatales	f:Mycoplasmataceae	g:Ca_Moeniiplasma	OTU_109
k:Bacteria p:Tenericutes	c:Mollicutes	o:Mycoplasmatales	f:Mycoplasmataceae	g:Ca_Moeniiplasma	OTU_112
k:Bacteria p:Tenericutes	c:Mollicutes	o:Mycoplasmatales	f:Mycoplasmataceae	g:Ca_Moeniiplasma	OTU_1184
k:Bacteria p:Tenericutes	c:Mollicutes	o:Mycoplasmatales	f:Mycoplasmataceae	g:Ca_Moeniiplasma	OTU_119
k:Bacteria p:Tenericutes	c:Mollicutes	o:Mycoplasmatales	f:Mycoplasmataceae	g:Ca_Moeniiplasma	OTU_11971
k:Bacteria p:Tenericutes	c:Mollicutes	o:Mycoplasmatales	f:Mycoplasmataceae	g:Ca_Moeniiplasma	OTU_1199
k:Bacteria p:Tenericutes	c:Mollicutes	o:Mycoplasmatales	f:Mycoplasmataceae	g:Ca_Moeniiplasma	OTU_1262
k:Bacteria p:Tenericutes	c:Mollicutes	o:Mycoplasmatales	f:Mycoplasmataceae	g:Ca_Moeniiplasma	OTU_1263
k:Bacteria p:Tenericutes	c:Mollicutes	o:Mycoplasmatales	f:Mycoplasmataceae	g:Ca_Moeniiplasma	OTU_14920
k:Bacteria p:Tenericutes	c:Mollicutes	o:Mycoplasmatales	f:Mycoplasmataceae	g:Ca_Moeniiplasma	OTU_1497
k:Bacteria p:Tenericutes	c:Mollicutes	o:Mycoplasmatales	f:Mycoplasmataceae	g:Ca_Moeniiplasma	OTU_150
k:Bacteria p:Tenericutes	c:Mollicutes	o:Mycoplasmatales	f:Mycoplasmataceae	g:Ca_Moeniiplasma	OTU_151
k:Bacteria p:Tenericutes	c:Mollicutes	o:Mycoplasmatales	f:Mycoplasmataceae	g:Ca_Moeniiplasma	OTU_1536
k:Bacteria p:Tenericutes	c:Mollicutes	o:Mycoplasmatales	f:Mycoplasmataceae	g:Ca_Moeniiplasma	OTU_1631
k:Bacteria p:Tenericutes	c:Mollicutes	o:Mycoplasmatales	f:Mycoplasmataceae	g:Ca_Moeniiplasma	OTU_1649
k:Bacteria p:Tenericutes	c:Mollicutes	o:Mycoplasmatales	f:Mycoplasmataceae	g:Ca_Moeniiplasma	OTU_1799
k:Bacteria p:Tenericutes	c:Mollicutes	o:Mycoplasmatales	f:Mycoplasmataceae	g:Ca_Moeniiplasma	OTU_18400
k:Bacteria p:Tenericutes	c:Mollicutes	o:Mycoplasmatales	f:Mycoplasmataceae	g:Ca_Moeniiplasma	OTU_1879
k:Bacteria p:Tenericutes	c:Mollicutes	o:Mycoplasmatales	f:Mycoplasmataceae	g:Ca_Moeniiplasma	OTU_2083
k:Bacteria p:Tenericutes	c:Mollicutes	o:Mycoplasmatales	f:Mycoplasmataceae	g:Ca_Moeniiplasma	OTU_21120
k:Bacteria p:Tenericutes	c:Mollicutes	o:Mycoplasmatales	f:Mycoplasmataceae	g:Ca_Moeniiplasma	OTU_2126
k:Bacteria p:Tenericutes	c:Mollicutes	o:Mycoplasmatales	f:Mycoplasmataceae	g:Ca_Moeniiplasma	OTU_2276

k:Bacteria	p:Tenericutes	c:Mollicutes	o:Mycoplasmatales	f:Mycoplasmataceae	g:Ca_Moeniiplasma	OTU_2322
k:Bacteria	p:Tenericutes	c:Mollicutes	o:Mycoplasmatales	f:Mycoplasmataceae	g:Ca_Moeniiplasma	OTU_244
k:Bacteria	p:Tenericutes	c:Mollicutes	o:Mycoplasmatales	f:Mycoplasmataceae	g:Ca_Moeniiplasma	OTU_2445
k:Bacteria	p:Tenericutes	c:Mollicutes	o:Mycoplasmatales	f:Mycoplasmataceae	g:Ca_Moeniiplasma	OTU_2446
k:Bacteria	p:Tenericutes	c:Mollicutes	o:Mycoplasmatales	f:Mycoplasmataceae	g:Ca_Moeniiplasma	OTU_2489
k:Bacteria	p:Tenericutes	c:Mollicutes	o:Mycoplasmatales	f:Mycoplasmataceae	g:Ca_Moeniiplasma	OTU_25136
k:Bacteria	p:Tenericutes	c:Mollicutes	o:Mycoplasmatales	f:Mycoplasmataceae	g:Ca_Moeniiplasma	OTU_257
k:Bacteria	p:Tenericutes	c:Mollicutes	o:Mycoplasmatales	f:Mycoplasmataceae	g:Ca_Moeniiplasma	OTU_27
k:Bacteria	p:Tenericutes	c:Mollicutes	o:Mycoplasmatales	f:Mycoplasmataceae	g:Ca_Moeniiplasma	OTU_2715
k:Bacteria	p:Tenericutes	c:Mollicutes	o:Mycoplasmatales	f:Mycoplasmataceae	g:Ca_Moeniiplasma	OTU_2826
k:Bacteria	p:Tenericutes	c:Mollicutes	o:Mycoplasmatales	f:Mycoplasmataceae	g:Ca_Moeniiplasma	OTU_295
k:Bacteria	p:Tenericutes	c:Mollicutes	o:Mycoplasmatales	f:Mycoplasmataceae	g:Ca_Moeniiplasma	OTU_3014
k:Bacteria	p:Tenericutes	c:Mollicutes	o:Mycoplasmatales	f:Mycoplasmataceae	g:Ca_Moeniiplasma	OTU_3062
k:Bacteria	p:Tenericutes	c:Mollicutes	o:Mycoplasmatales	f:Mycoplasmataceae	g:Ca_Moeniiplasma	OTU_3159
k:Bacteria	p:Tenericutes	c:Mollicutes	o:Mycoplasmatales	f:Mycoplasmataceae	g:Ca_Moeniiplasma	OTU_3160
k:Bacteria	p:Tenericutes	c:Mollicutes	o:Mycoplasmatales	f:Mycoplasmataceae	g:Ca_Moeniiplasma	OTU_3209
k:Bacteria	p:Tenericutes	c:Mollicutes	o:Mycoplasmatales	f:Mycoplasmataceae	g:Ca_Moeniiplasma	OTU_3239
k:Bacteria	p:Tenericutes	c:Mollicutes	o:Mycoplasmatales	f:Mycoplasmataceae	g:Ca_Moeniiplasma	OTU_350
k:Bacteria	p:Tenericutes	c:Mollicutes	o:Mycoplasmatales	f:Mycoplasmataceae	g:Ca_Moeniiplasma	OTU_354
k:Bacteria	p:Tenericutes	c:Mollicutes	o:Mycoplasmatales	f:Mycoplasmataceae	g:Ca_Moeniiplasma	OTU_3722
k:Bacteria	p:Tenericutes	c:Mollicutes	o:Mycoplasmatales	f:Mycoplasmataceae	g:Ca_Moeniiplasma	OTU_4062
k:Bacteria	p:Tenericutes	c:Mollicutes	o:Mycoplasmatales	f:Mycoplasmataceae	g:Ca_Moeniiplasma	OTU_4064
k:Bacteria	p:Tenericutes	c:Mollicutes	o:Mycoplasmatales	f:Mycoplasmataceae	g:Ca_Moeniiplasma	OTU_4065
k:Bacteria	p:Tenericutes	c:Mollicutes	o:Mycoplasmatales	f:Mycoplasmataceae	g:Ca_Moeniiplasma	OTU_42
k:Bacteria	p:Tenericutes	c:Mollicutes	o:Mycoplasmatales	f:Mycoplasmataceae	g:Ca_Moeniiplasma	OTU_52
k:Bacteria	p:Tenericutes	c:Mollicutes	o:Mycoplasmatales	f:Mycoplasmataceae	g:Ca_Moeniiplasma	OTU_526
k:Bacteria	p:Tenericutes	c:Mollicutes	o:Mycoplasmatales	f:Mycoplasmataceae	g:Ca_Moeniiplasma	OTU_533
k:Bacteria	p:Tenericutes	c:Mollicutes	o:Mycoplasmatales	f:Mycoplasmataceae	g:Ca_Moeniiplasma	OTU_5576
k:Bacteria	p:Tenericutes	c:Mollicutes	o:Mycoplasmatales	f:Mycoplasmataceae	g:Ca_Moeniiplasma	OTU_57
k:Bacteria	p:Tenericutes	c:Mollicutes	o:Mycoplasmatales	f:Mycoplasmataceae	g:Ca_Moeniiplasma	OTU_578
k:Bacteria	p:Tenericutes	c:Mollicutes	o:Mycoplasmatales	f:Mycoplasmataceae	g:Ca_Moeniiplasma	OTU_6038
k:Bacteria	p:Tenericutes	c:Mollicutes	o:Mycoplasmatales	f:Mycoplasmataceae	g:Ca_Moeniiplasma	OTU_6040
k:Bacteria	p:Tenericutes	c:Mollicutes	o:Mycoplasmatales	f:Mycoplasmataceae	g:Ca_Moeniiplasma	OTU_6506
k:Bacteria	p:Tenericutes	c:Mollicutes	o:Mycoplasmatales	f:Mycoplasmataceae	g:Ca_Moeniiplasma	OTU_6507
k:Bacteria	p:Tenericutes	c:Mollicutes	o:Mycoplasmatales	f:Mycoplasmataceae	g:Ca_Moeniiplasma	OTU_672

k:Bacteria p:Tenericutes	c:Mollicutes	o:Mycoplasmatales	f:Mycoplasmataceae	g:Ca_Moeniiplasma	OTU_685
k:Bacteria p:Tenericutes	c:Mollicutes	o:Mycoplasmatales	f:Mycoplasmataceae	g:Ca_Moeniiplasma	OTU_691
k:Bacteria p:Tenericutes	c:Mollicutes	o:Mycoplasmatales	f:Mycoplasmataceae	g:Ca_Moeniiplasma	OTU_7233
k:Bacteria p:Tenericutes	c:Mollicutes	o:Mycoplasmatales	f:Mycoplasmataceae	g:Ca_Moeniiplasma	OTU_8537
k:Bacteria p:Tenericutes	c:Mollicutes	o:Mycoplasmatales	f:Mycoplasmataceae	g:Ca_Moeniiplasma	OTU_876
k:Bacteria p:Tenericutes	c:Mollicutes	o:Mycoplasmatales	f:Mycoplasmataceae	g:Ca_Moeniiplasma	OTU_9242
k:Bacteria p:Tenericutes	c:Mollicutes	o:Mycoplasmatales	f:Mycoplasmataceae	g:Ca_Moeniiplasma	OTU_948
k:Bacteria p:Tenericutes	c:Mollicutes	o:Mycoplasmatales	f:Mycoplasmataceae	g:Ca_Moeniiplasma	OTU_989
k:Bacteria p:Proteobacteria	c:Deltaproteobacteria	o:Myxococcales	f:BIrii41	NA	OTU_1571
k:Bacteria p:Proteobacteria	c:Deltaproteobacteria	o:Myxococcales	f:Sandaracinaceae	g:Sandaracinus	OTU_1572
k:Bacteria p:Proteobacteria	c:Deltaproteobacteria	o:Myxococcales	NA	NA	OTU_2137
k:Bacteria p:Proteobacteria	c:Deltaproteobacteria	o:Myxococcales	f:Sandaracinaceae	NA	OTU_228
k:Bacteria p:Proteobacteria	c:Deltaproteobacteria	o:Myxococcales	f:mle1-27	NA	OTU_2409
k:Bacteria p:Proteobacteria	c:Deltaproteobacteria	o:Myxococcales	f:Haliangiaceae	g:Haliangium	OTU_2719
k:Bacteria p:Proteobacteria	c:Deltaproteobacteria	o:Myxococcales	f:Haliangiaceae	g:Haliangium	OTU_2931
k:Bacteria p:Proteobacteria	c:Deltaproteobacteria	o:Myxococcales	f:Haliangiaceae	g:Haliangium	OTU_373
k:Bacteria p:Proteobacteria	c:Deltaproteobacteria	o:Myxococcales	f:Polyangiaceae	g:Sorangium	OTU_6675
k:Bacteria p:Proteobacteria	c:Deltaproteobacteria	o:Myxococcales	f:Sandaracinaceae	NA	OTU_688
k:Bacteria p:Proteobacteria	c:Deltaproteobacteria	o:Myxococcales	NA	NA	OTU_798
k:Bacteria p:Proteobacteria	c:Deltaproteobacteria	o:Myxococcales	f:Haliangiaceae	g:Haliangium	OTU_801
k:Bacteria p:Proteobacteria	c:Betaproteobacteria	o:Neisseriales	f:Neisseriaceae	g:Vogesella	OTU_1241
k:Bacteria p:Proteobacteria	c:Betaproteobacteria	o:Neisseriales	f:Neisseriaceae	NA	OTU_1524
k:Bacteria p:Proteobacteria	c:Betaproteobacteria	o:Neisseriales	f:Neisseriaceae	g:Neisseria	OTU_195
k:Bacteria p:Proteobacteria	c:Betaproteobacteria	o:Neisseriales	f:Neisseriaceae	NA	OTU_2941
k:Bacteria p:Proteobacteria	c:Betaproteobacteria	o:Neisseriales	f:Neisseriaceae	g:Neisseria	OTU_416
k:Bacteria p:Proteobacteria	c:Betaproteobacteria	o:Neisseriales	f:Neisseriaceae	g:Kingella	OTU_765
k:Bacteria p:Actinobacteria	c:Nitriliruptoria	o:Nitriliruptorales	f:Nitriliruptoraceae	g:Nitriliruptor	OTU_1711
k:Bacteria p:Proteobacteria	c:Betaproteobacteria	o:Nitrosomonadales	f:Nitrosomonadaceae	NA	OTU_1105
k:Bacteria p:Proteobacteria	c:Betaproteobacteria	o:Nitrosomonadales	f:Nitrosomonadaceae	NA	OTU_1317
k:Bacteria p:Proteobacteria	c:Betaproteobacteria	o:Nitrosomonadales	f:Nitrosomonadaceae	NA	OTU_241
k:Bacteria p:Proteobacteria	c:Betaproteobacteria	o:Nitrosomonadales	f:Nitrosomonadaceae	NA	OTU_420
k:Bacteria p:Proteobacteria	c:Deltaproteobacteria	o:Oligoflexales	NA	NA	OTU_1222
k:Bacteria p:Proteobacteria	c:Deltaproteobacteria	o:Oligoflexales	NA	NA	OTU_442
k:Bacteria p:Verrucomicrobia	c:Opitutae	o:Opitutales	f:Opitutaceae	g:Opitutus	OTU_232
k:Bacteria p:Verrucomicrobia	c:Opitutae	o:Opitutales	f:Opitutaceae	g:Opitutus	OTU_534

k:Bacteria p:Proteobacteria	c:Gammaproteobacteria	o:Order_Incertae_Sedis	f:Family_Incertae_Sedis	g:Marinicella	OTU_480
k:Bacteria p:Proteobacteria	c:Gammaproteobacteria	o:Pasteurellales	f:Pasteurellaceae	g:Haemophilus	OTU_10174
k:Bacteria p:Proteobacteria	c:Gammaproteobacteria	o:Pasteurellales	f:Pasteurellaceae	g:Haemophilus	OTU_139
k:Bacteria p:Proteobacteria	c:Gammaproteobacteria	o:Pasteurellales	f:Pasteurellaceae	NA	OTU_429
k:Bacteria p:Proteobacteria	c:Gammaproteobacteria	o:Pasteurellales	f:Pasteurellaceae	g:Aggregatibacter	OTU_514
k:Bacteria p:Planctomycetes	c:Planctomycetacia	o:Planctomycetales	f:Planctomycetaceae	g:Pirellula	OTU_1256
k:Bacteria p:Planctomycetes	c:Planctomycetacia	o:Planctomycetales	f:Planctomycetaceae	NA	OTU_359
k:Bacteria p:Actinobacteria	c:Actinobacteria	o:Propionibacteriales	f:Propionibacteriaceae	g:Propionibacterium	OTU_130
k:Bacteria p:Actinobacteria	c:Actinobacteria	o:Propionibacteriales	f:Nocardioidaceae	g:Nocardioides	OTU_1314
k:Bacteria p:Actinobacteria	c:Actinobacteria	o:Propionibacteriales	f:Nocardioidaceae	g:Nocardioides	OTU_136
k:Bacteria p:Actinobacteria	c:Actinobacteria	o:Propionibacteriales	f:Nocardioidaceae	g:Nocardioides	OTU_183
k:Bacteria p:Actinobacteria	c:Actinobacteria	o:Propionibacteriales	f:Nocardioidaceae	g:Kribbella	OTU_235
k:Bacteria p:Actinobacteria	c:Actinobacteria	o:Propionibacteriales	f:Nocardioidaceae	g:Nocardioides	OTU_271
k:Bacteria p:Actinobacteria	c:Actinobacteria	o:Propionibacteriales	f:Propionibacteriaceae	g:Propionibacterium	OTU_2994
k:Bacteria p:Actinobacteria	c:Actinobacteria	o:Propionibacteriales	f:Propionibacteriaceae	NA	OTU_601
k:Bacteria p:Actinobacteria	c:Actinobacteria	o:Propionibacteriales	f:Nocardioidaceae	g:Nocardioides	OTU_862
k:Bacteria p:Proteobacteria	c:Gammaproteobacteria	o:Pseudomonadales	f:Moraxellaceae	g:Acinetobacter	OTU_1072
k:Bacteria p:Proteobacteria	c:Gammaproteobacteria	o:Pseudomonadales	f:Pseudomonadaceae	g:Pseudomonas	OTU_11
k:Bacteria p:Proteobacteria	c:Gammaproteobacteria	o:Pseudomonadales	f:Pseudomonadaceae	g:Pseudomonas	OTU_1112
k:Bacteria p:Proteobacteria	c:Gammaproteobacteria	o:Pseudomonadales	f:Pseudomonadaceae	g:Pseudomonas	OTU_1372
k:Bacteria p:Proteobacteria	c:Gammaproteobacteria	o:Pseudomonadales	f:Moraxellaceae	g:Moraxella	OTU_175
k:Bacteria p:Proteobacteria	c:Gammaproteobacteria	o:Pseudomonadales	f:Pseudomonadaceae	g:Pseudomonas	OTU_202
k:Bacteria p:Proteobacteria	c:Gammaproteobacteria	o:Pseudomonadales	f:Pseudomonadaceae	g:Pseudomonas	OTU_25191
k:Bacteria p:Proteobacteria	c:Gammaproteobacteria	o:Pseudomonadales	f:Pseudomonadaceae	g:Pseudomonas	OTU_30
k:Bacteria p:Proteobacteria	c:Gammaproteobacteria	o:Pseudomonadales	f:Pseudomonadaceae	g:Pseudomonas	OTU_3157
k:Bacteria p:Proteobacteria	c:Gammaproteobacteria	o:Pseudomonadales	f:Pseudomonadaceae	g:Pseudomonas	OTU_3717
k:Bacteria p:Proteobacteria	c:Gammaproteobacteria	o:Pseudomonadales	f:Pseudomonadaceae	g:Pseudomonas	OTU_581
k:Bacteria p:Proteobacteria	c:Gammaproteobacteria	o:Pseudomonadales	f:Pseudomonadaceae	g:Pseudomonas	OTU_6185
k:Bacteria p:Proteobacteria	c:Gammaproteobacteria	o:Pseudomonadales	f:Pseudomonadaceae	g:Pseudomonas	OTU_7227
k:Bacteria p:Proteobacteria	c:Gammaproteobacteria	o:Pseudomonadales	f:Pseudomonadaceae	g:Pseudomonas	OTU_806
k:Bacteria p:Proteobacteria	c:Gammaproteobacteria	o:Pseudomonadales	f:Pseudomonadaceae	g:Pseudomonas	OTU_8441
k:Bacteria p:Proteobacteria	c:Gammaproteobacteria	o:Pseudomonadales	f:Moraxellaceae	g:Acinetobacter	OTU_93
k:Bacteria p:Actinobacteria	c:Actinobacteria	o:Pseudonocardiales	f:Pseudonocardiaceae	g:Amycolatopsis	OTU_100
k:Bacteria p:Actinobacteria	c:Actinobacteria	o:Pseudonocardiales	f:Pseudonocardiaceae	g:Pseudonocardia	OTU_101
k:Bacteria p:Actinobacteria	c:Actinobacteria	o:Pseudonocardiales	f:Pseudonocardiaceae	g:Pseudonocardia	OTU_1043

k:Bacteria	p:Actinobacteria	c:Actinobacteria	o:Pseudonocardiales	f:Pseudonocardiaceae	NA	OTU_105
k:Bacteria	p:Actinobacteria	c:Actinobacteria	o:Pseudonocardiales	f:Pseudonocardiaceae	g:Amycolatopsis	OTU_10983
k:Bacteria	p:Actinobacteria	c:Actinobacteria	o:Pseudonocardiales	f:Pseudonocardiaceae	NA	OTU_1119
k:Bacteria	p:Actinobacteria	c:Actinobacteria	o:Pseudonocardiales	f:Pseudonocardiaceae	g:Actinophytocola	OTU_1225
k:Bacteria	p:Actinobacteria	c:Actinobacteria	o:Pseudonocardiales	f:Pseudonocardiaceae	g:Pseudonocardia	OTU_1569
k:Bacteria	p:Actinobacteria	c:Actinobacteria	o:Pseudonocardiales	f:Pseudonocardiaceae	NA	OTU_1779
k:Bacteria	p:Actinobacteria	c:Actinobacteria	o:Pseudonocardiales	f:Pseudonocardiaceae	g:Actinoalloteichus	OTU_2454
k:Bacteria	p:Actinobacteria	c:Actinobacteria	o:Pseudonocardiales	f:Pseudonocardiaceae	g:Pseudonocardia	OTU_2602
k:Bacteria	p:Actinobacteria	c:Actinobacteria	o:Pseudonocardiales	f:Pseudonocardiaceae	NA	OTU_2714
k:Bacteria	p:Actinobacteria	c:Actinobacteria	o:Pseudonocardiales	f:Pseudonocardiaceae	NA	OTU_2760
k:Bacteria	p:Actinobacteria	c:Actinobacteria	o:Pseudonocardiales	f:Pseudonocardiaceae	NA	OTU_32324
k:Bacteria	p:Actinobacteria	c:Actinobacteria	o:Pseudonocardiales	f:Pseudonocardiaceae	g:Pseudonocardia	OTU_3403
k:Bacteria	p:Actinobacteria	c:Actinobacteria	o:Pseudonocardiales	f:Pseudonocardiaceae	g:Pseudonocardia	OTU_3543
k:Bacteria	p:Actinobacteria	c:Actinobacteria	o:Pseudonocardiales	f:Pseudonocardiaceae	g:Pseudonocardia	OTU_40
k:Bacteria	p:Actinobacteria	c:Actinobacteria	o:Pseudonocardiales	f:Pseudonocardiaceae	g:Pseudonocardia	OTU_546
k:Bacteria	p:Actinobacteria	c:Actinobacteria	o:Pseudonocardiales	f:Pseudonocardiaceae	g:Saccharothrix	OTU_854
k:Bacteria	p:Actinobacteria	c:Actinobacteria	o:Pseudonocardiales	f:Pseudonocardiaceae	g:Lentzea	OTU_94
k:Bacteria	p:Actinobacteria	c:Actinobacteria	o:Pseudonocardiales	f:Pseudonocardiaceae	g:Saccharopolyspora	OTU_994
k:Bacteria	p:Proteobacteria	c:Alphaproteobacteria	o:Rhizobiales	f:Xanthobacteraceae	g:Variibacter	OTU_106
k:Bacteria	p:Proteobacteria	c:Alphaproteobacteria	o:Rhizobiales	f:Methylobacteriaceae	g:Microvirga	OTU_111
k:Bacteria	p:Proteobacteria	c:Alphaproteobacteria	o:Rhizobiales	NA	NA	OTU_1146
k:Bacteria	p:Proteobacteria	c:Alphaproteobacteria	o:Rhizobiales	NA	NA	OTU_1201
k:Bacteria	p:Proteobacteria	c:Alphaproteobacteria	o:Rhizobiales	f:Hyphomicrobiaceae	g:Rhodomicrobium	OTU_1239
k:Bacteria	p:Proteobacteria	c:Alphaproteobacteria	o:Rhizobiales	f:Methylobacteriaceae	NA	OTU_1243
k:Bacteria	p:Proteobacteria	c:Alphaproteobacteria	o:Rhizobiales	f:Rhizobiaceae	g:Rhizobium	OTU_1270
k:Bacteria	p:Proteobacteria	c:Alphaproteobacteria	o:Rhizobiales	f:Hyphomicrobiaceae	g:Devosia	OTU_1289
k:Bacteria	p:Proteobacteria	c:Alphaproteobacteria	o:Rhizobiales	f:Hyphomicrobiaceae	NA	OTU_129
k:Bacteria	p:Proteobacteria	c:Alphaproteobacteria	o:Rhizobiales	NA	NA	OTU_1373
k:Bacteria	p:Proteobacteria	c:Alphaproteobacteria	o:Rhizobiales	NA	NA	OTU_1450
k:Bacteria	p:Proteobacteria	c:Alphaproteobacteria	o:Rhizobiales	NA	NA	OTU_1466
k:Bacteria	p:Proteobacteria	c:Alphaproteobacteria	o:Rhizobiales	f:Bradyrhizobiaceae	g:Salinarimonas	OTU_16377
k:Bacteria	p:Proteobacteria	c:Alphaproteobacteria	o:Rhizobiales	f:Rhizobiaceae	NA	OTU_1662
k:Bacteria	p:Proteobacteria	c:Alphaproteobacteria	o:Rhizobiales	f:JG34-KF-361	NA	OTU_1746
k:Bacteria	p:Proteobacteria	c:Alphaproteobacteria	o:Rhizobiales	f:Hyphomicrobiaceae	g:Rhodomicrobium	OTU_1795
k:Bacteria	p:Proteobacteria	c:Alphaproteobacteria	o:Rhizobiales	f:Rhizobiaceae	g:Rhizobium	OTU_19

k:Bacteria	p:Proteobacteria	c:Alphaproteobacteria	o:Rhizobiales	f:Bradyrhizobiaceae	g:Bosea	OTU_200
k:Bacteria	p:Proteobacteria	c:Alphaproteobacteria	o:Rhizobiales	NA	NA	OTU_2305
k:Bacteria	p:Proteobacteria	c:Alphaproteobacteria	o:Rhizobiales	f:Rhizobiaceae	g:Rhizobium	OTU_23661
k:Bacteria	p:Proteobacteria	c:Alphaproteobacteria	o:Rhizobiales	f:Xanthobacteraceae	g:Pseudolabrys	OTU_2484
k:Bacteria	p:Proteobacteria	c:Alphaproteobacteria	o:Rhizobiales	f:Bradyrhizobiaceae	g:Nitrobacter	OTU_2529
k:Bacteria	p:Proteobacteria	c:Alphaproteobacteria	o:Rhizobiales	NA	NA	OTU_255
k:Bacteria	p:Proteobacteria	c:Alphaproteobacteria	o:Rhizobiales	f:Rhizobiaceae	g:Ensifer	OTU_259
k:Bacteria	p:Proteobacteria	c:Alphaproteobacteria	o:Rhizobiales	f:Rhizobiales_Incertae_Sec	g:Bauldia	OTU_289
k:Bacteria	p:Proteobacteria	c:Alphaproteobacteria	o:Rhizobiales	f:Phyllobacteriaceae	g:Nitratireductor	OTU_296
k:Bacteria	p:Proteobacteria	c:Alphaproteobacteria	o:Rhizobiales	f:Methylobacteriaceae	NA	OTU_300
k:Bacteria	p:Proteobacteria	c:Alphaproteobacteria	o:Rhizobiales	f:Rhizobiaceae	g:Rhizobium	OTU_3027
k:Bacteria	p:Proteobacteria	c:Alphaproteobacteria	o:Rhizobiales	f:Rhizobiaceae	g:Rhizobium	OTU_3129
k:Bacteria	p:Proteobacteria	c:Alphaproteobacteria	o:Rhizobiales	f:Hyphomicrobiaceae	g:Rhodomicrobium	OTU_315
k:Bacteria	p:Proteobacteria	c:Alphaproteobacteria	o:Rhizobiales	NA	NA	OTU_317
k:Bacteria	p:Proteobacteria	c:Alphaproteobacteria	o:Rhizobiales	f:Rhizobiaceae	g:Rhizobium	OTU_32722
k:Bacteria	p:Proteobacteria	c:Alphaproteobacteria	o:Rhizobiales	f:Hyphomicrobiaceae	g:Rhodomicrobium	OTU_3561
k:Bacteria	p:Proteobacteria	c:Alphaproteobacteria	o:Rhizobiales	NA	NA	OTU_433
k:Bacteria	p:Proteobacteria	c:Alphaproteobacteria	o:Rhizobiales	f:Bradyrhizobiaceae	g:Bradyrhizobium	OTU_45
k:Bacteria	p:Proteobacteria	c:Alphaproteobacteria	o:Rhizobiales	f:Bradyrhizobiaceae	g:Bradyrhizobium	OTU_4671
k:Bacteria	p:Proteobacteria	c:Alphaproteobacteria	o:Rhizobiales	f:Phyllobacteriaceae	g:Phyllobacterium	OTU_468
k:Bacteria	p:Proteobacteria	c:Alphaproteobacteria	o:Rhizobiales	f:Rhizobiaceae	NA	OTU_4807
k:Bacteria	p:Proteobacteria	c:Alphaproteobacteria	o:Rhizobiales	f:Hyphomicrobiaceae	g:Pedomicrobium	OTU_482
k:Bacteria	p:Proteobacteria	c:Alphaproteobacteria	o:Rhizobiales	f:Rhizobiaceae	g:Shinella	OTU_501
k:Bacteria	p:Proteobacteria	c:Alphaproteobacteria	o:Rhizobiales	NA	NA	OTU_510
k:Bacteria	p:Proteobacteria	c:Alphaproteobacteria	o:Rhizobiales	f:Hyphomicrobiaceae	g:Pedomicrobium	OTU_574
k:Bacteria	p:Proteobacteria	c:Alphaproteobacteria	o:Rhizobiales	f:Rhizobiaceae	NA	OTU_5767
k:Bacteria	p:Proteobacteria	c:Alphaproteobacteria	o:Rhizobiales	f:Rhizobiaceae	g:Rhizobium	OTU_58
k:Bacteria	p:Proteobacteria	c:Alphaproteobacteria	o:Rhizobiales	f:Hyphomicrobiaceae	g:Hyphomicrobium	OTU_6020
k:Bacteria	p:Proteobacteria	c:Alphaproteobacteria	o:Rhizobiales	f:Methylobacteriaceae	NA	OTU_648
k:Bacteria	p:Proteobacteria	c:Alphaproteobacteria	o:Rhizobiales	NA	NA	OTU_731
k:Bacteria	p:Proteobacteria	c:Alphaproteobacteria	o:Rhizobiales	f:Hyphomicrobiaceae	g:Devosia	OTU_75
k:Bacteria	p:Proteobacteria	c:Alphaproteobacteria	o:Rhizobiales	f:Phyllobacteriaceae	g:Aquamicrobium	OTU_797
k:Bacteria	p:Proteobacteria	c:Alphaproteobacteria	o:Rhizobiales	f:Hyphomicrobiaceae	g:Pedomicrobium	OTU_820
k:Bacteria	p:Proteobacteria	c:Alphaproteobacteria	o:Rhizobiales	f:Rhizobiales_Incertae_Sec	g:Agaricicola	OTU_875
k:Bacteria	p:Proteobacteria	c:Alphaproteobacteria	o:Rhizobiales	f:Hyphomicrobiaceae	NA	OTU_8878

k:Bacteria	p:Proteobacteria	c:Alphaproteobacteria	o:Rhodobacterales	f:Rhodobacteraceae	g:Paracocccus	OTU_123
k:Bacteria	p:Proteobacteria	c:Alphaproteobacteria	o:Rhodobacterales	f:Rhodobacteraceae	g:Rubellimicrobium	OTU_286
k:Bacteria	p:Proteobacteria	c:Alphaproteobacteria	o:Rhodobacterales	f:Rhodobacteraceae	g:Rubellimicrobium	OTU_403
k:Bacteria	p:Proteobacteria	c:Alphaproteobacteria	o:Rhodobacterales	f:Rhodobacteraceae	g:Paracocccus	OTU_70
k:Bacteria	p:Proteobacteria	c:Betaproteobacteria	o:Rhodocyclales	f:Rhodocyclaceae	NA	OTU_1215
k:Bacteria	p:Proteobacteria	c:Alphaproteobacteria	o:Rhodospirillales	NA	NA	OTU_1516
k:Bacteria	p:Proteobacteria	c:Alphaproteobacteria	o:Rhodospirillales	f:Rhodospirillaceae	g:Azospirillum	OTU_1947
k:Bacteria	p:Proteobacteria	c:Alphaproteobacteria	o:Rhodospirillales	f:Rhodospirillaceae	g:Skermanella	OTU_290
k:Bacteria	p:Proteobacteria	c:Alphaproteobacteria	o:Rhodospirillales	f:Rhodospirillales_Incertae_	g:Reyranella	OTU_3357
k:Bacteria	p:Proteobacteria	c:Alphaproteobacteria	o:Rhodospirillales	f:Acetobacteraceae	NA	OTU_369
k:Bacteria	p:Proteobacteria	c:Alphaproteobacteria	o:Rhodospirillales	f:Rhodospirillaceae	g:Inquilinus	OTU_406
k:Bacteria	p:Proteobacteria	c:Alphaproteobacteria	o:Rhodospirillales	f:I-10	NA	OTU_457
k:Bacteria	p:Proteobacteria	c:Alphaproteobacteria	o:Rhodospirillales	f:Rhodospirillaceae	g:Azospirillum	OTU_50
k:Bacteria	p:Proteobacteria	c:Alphaproteobacteria	o:Rhodospirillales	f:Rhodospirillaceae	g:Ferrovibrio	OTU_536
k:Bacteria	p:Proteobacteria	c:Alphaproteobacteria	o:Rhodospirillales	f:Rhodospirillaceae	NA	OTU_730
k:Bacteria	p:Gemmatimonadetes	c:Gemmatimonadetes	o:S0134_terrestrial_gro	NA	NA	OTU_1400
k:Bacteria	p:Tenericutes	c:Negativicutes	o:Selenomonadales	f:Veillonellaceae	g:Veillonella	OTU_1456
k:Bacteria	p:Tenericutes	c:Negativicutes	o:Selenomonadales	f:Veillonellaceae	g:Veillonella	OTU_668
k:Bacteria	p:Actinobacteria	c:Actinobacteria	o:SIFF498-N9D4	NA	NA	OTU_594
k:Bacteria	p:Actinobacteria	c:Thermoleophilia	o:Solirubrobacterales	NA	NA	OTU_117
k:Bacteria	p:Actinobacteria	c:Thermoleophilia	o:Solirubrobacterales	f:Patulibacteraceae	g:Patulibacter	OTU_120
k:Bacteria	p:Actinobacteria	c:Thermoleophilia	o:Solirubrobacterales	f:480-2	NA	OTU_1452
k:Bacteria	p:Actinobacteria	c:Thermoleophilia	o:Solirubrobacterales	f:Solirubrobacteraceae	g:Solirubrobacter	OTU_156
k:Bacteria	p:Actinobacteria	c:Thermoleophilia	o:Solirubrobacterales	f:480-2	NA	OTU_1757
k:Bacteria	p:Actinobacteria	c:Thermoleophilia	o:Solirubrobacterales	f:480-2	NA	OTU_1874
k:Bacteria	p:Actinobacteria	c:Thermoleophilia	o:Solirubrobacterales	f:YNPFFP1	NA	OTU_212
k:Bacteria	p:Actinobacteria	c:Thermoleophilia	o:Solirubrobacterales	f:Solirubrobacteraceae	g:Solirubrobacter	OTU_2527
k:Bacteria	p:Actinobacteria	c:Thermoleophilia	o:Solirubrobacterales	f:480-2	NA	OTU_254
k:Bacteria	p:Actinobacteria	c:Thermoleophilia	o:Solirubrobacterales	f:480-2	NA	OTU_361
k:Bacteria	p:Actinobacteria	c:Thermoleophilia	o:Solirubrobacterales	f:480-2	NA	OTU_398
k:Bacteria	p:Actinobacteria	c:Thermoleophilia	o:Solirubrobacterales	f:Solirubrobacteraceae	g:Solirubrobacter	OTU_4909
k:Bacteria	p:Actinobacteria	c:Thermoleophilia	o:Solirubrobacterales	f:0319-6M6	NA	OTU_503
k:Bacteria	p:Actinobacteria	c:Thermoleophilia	o:Solirubrobacterales	f:Solirubrobacteraceae	g:Solirubrobacter	OTU_55
k:Bacteria	p:Actinobacteria	c:Thermoleophilia	o:Solirubrobacterales	f:Elev-16S-1332	NA	OTU_561
k:Bacteria	p:Actinobacteria	c:Thermoleophilia	o:Solirubrobacterales	f:Patulibacteraceae	g:Patulibacter	OTU_706

k:Bacteria	p:Actinobacteria	c:Thermoleophilia	o:Solirubrobacterales	NA	NA	OTU_711
k:Bacteria	p:Bacteroidetes	c:Sphingobacteriia	o:Sphingobacteriales	f:Chitinophagaceae	NA	OTU_1089
k:Bacteria	p:Bacteroidetes	c:Sphingobacteriia	o:Sphingobacteriales	f:Chitinophagaceae	g:Chitinophaga	OTU_4333
k:Bacteria	p:Bacteroidetes	c:Sphingobacteriia	o:Sphingobacteriales	f:Chitinophagaceae	g:Flavitalea	OTU_542
k:Bacteria	p:Proteobacteria	c:Alphaproteobacteria	o:Sphingomonadales	f:Sphingomonadaceae	g:Sphingomonas	OTU_1118
k:Bacteria	p:Proteobacteria	c:Alphaproteobacteria	o:Sphingomonadales	f:Ellin6055	NA	OTU_167
k:Bacteria	p:Proteobacteria	c:Alphaproteobacteria	o:Sphingomonadales	f:Sphingomonadaceae	g:Sphingomonas	OTU_18
k:Bacteria	p:Proteobacteria	c:Alphaproteobacteria	o:Sphingomonadales	f:Sphingomonadaceae	g:Sphingomonas	OTU_3490
k:Bacteria	p:Proteobacteria	c:Alphaproteobacteria	o:Sphingomonadales	f:Erythrobacteraceae	g:Altererythrobacter	OTU_488
k:Bacteria	p:Proteobacteria	c:Alphaproteobacteria	o:Sphingomonadales	f:Sphingomonadaceae	g:Sphingobium	OTU_757
k:Bacteria	p:Proteobacteria	c:Alphaproteobacteria	o:Sphingomonadales	f:Sphingomonadaceae	g:Sphingomonas	OTU_89
k:Bacteria	p:Actinobacteria	c:Actinobacteria	o:Streptomycetales	f:Streptomycetaceae	g:Streptomyces	OTU_1385
k:Bacteria	p:Actinobacteria	c:Actinobacteria	o:Streptomycetales	f:Streptomycetaceae	g:Streptomyces	OTU_2302
k:Bacteria	p:Actinobacteria	c:Actinobacteria	o:Streptomycetales	f:Streptomycetaceae	g:Streptomyces	OTU_2375
k:Bacteria	p:Actinobacteria	c:Actinobacteria	o:Streptomycetales	f:Streptomycetaceae	NA	OTU_32
k:Bacteria	p:Actinobacteria	c:Actinobacteria	o:Streptomycetales	f:Streptomycetaceae	NA	OTU_3223
k:Bacteria	p:Actinobacteria	c:Actinobacteria	o:Streptomycetales	f:Streptomycetaceae	g:Streptomyces	OTU_35
k:Bacteria	p:Actinobacteria	c:Actinobacteria	o:Streptomycetales	f:Streptomycetaceae	NA	OTU_610
k:Bacteria	p:Actinobacteria	c:Actinobacteria	o:Streptomycetales	f:Streptomycetaceae	g:Streptomyces	OTU_7668
k:Bacteria	p:Actinobacteria	c:Actinobacteria	o:Streptosporangiales	f:Streptosporangiaceae	g:Microbispora	OTU_1090
k:Bacteria	p:Actinobacteria	c:Actinobacteria	o:Streptosporangiales	f:Thermomonosporaceae	g:Actinomadura	OTU_1353
k:Bacteria	p:Actinobacteria	c:Actinobacteria	o:Streptosporangiales	f:Thermomonosporaceae	g:Actinoallomurus	OTU_13752
k:Bacteria	p:Actinobacteria	c:Actinobacteria	o:Streptosporangiales	f:Thermomonosporaceae	g:Actinoallomurus	OTU_1937
k:Bacteria	p:Actinobacteria	c:Actinobacteria	o:Streptosporangiales	f:Thermomonosporaceae	g:Actinomadura	OTU_310
k:Bacteria	p:Actinobacteria	c:Actinobacteria	o:Streptosporangiales	f:Thermomonosporaceae	g:Actinoallomurus	OTU_32259
k:Bacteria	p:Actinobacteria	c:Actinobacteria	o:Streptosporangiales	f:Streptosporangiaceae	g:Nonomuraea	OTU_353
k:Bacteria	p:Actinobacteria	c:Actinobacteria	o:Streptosporangiales	f:Thermomonosporaceae	g:Actinomadura	OTU_611
k:Bacteria	p:Acidobacteria	c:Acidobacteria	o:Subgroup_3	f:Unknown_Family	g:Bryobacter	OTU_418
k:Bacteria	p:Acidobacteria	c:Acidobacteria	o:Subgroup_3	f:Unknown_Family	g:Ca_Solibacter	OTU_69
k:Bacteria	p:Acidobacteria	c:Acidobacteria	o:Subgroup_4	f:Unknown_Family	g:Blastocatella	OTU_294
k:Bacteria	p:Acidobacteria	c:Acidobacteria	o:Subgroup_4	f:Unknown_Family	g:Blastocatella	OTU_348
k:Bacteria	p:Acidobacteria	c:Acidobacteria	o:Subgroup_4	f:Unknown_Family	g:Blastocatella	OTU_823
k:Bacteria	p:Acidobacteria	c:Acidobacteria	o:Subgroup_6	NA	NA	OTU_1735
k:Bacteria	p:Acidobacteria	c:Acidobacteria	o:Subgroup_6	NA	NA	OTU_2205
k:Bacteria	p:Acidobacteria	c:Acidobacteria	o:Subgroup_6	NA	NA	OTU_248

k:Bacteria	p:Acidobacteria	c:Acidobacteria	o:Subgroup_6	NA	NA	OTU_621
k:Bacteria	p:Acidobacteria	c:Acidobacteria	o:Subgroup_6	NA	NA	OTU_735
k:Bacteria	p:Acidobacteria	c:Acidobacteria	o:Subgroup_6	NA	NA	OTU_906
k:Bacteria	p:Acidobacteria	c:Acidobacteria	o:Subgroup_6	NA	NA	OTU_997
k:Bacteria	p:Acidobacteria	c:Holophagae	o:Subgroup_7	NA	NA	OTU_804
k:Bacteria	p:Cyanobacteria	c:Cyanobacteria	o:SubsectionIII	f:FamilyI	g:Phormidium	OTU_126
k:Bacteria	p:Proteobacteria	c:Betaproteobacteria	o:TRA3-20	NA	NA	OTU_781
k:Bacteria	p:Planctomycetes	c:Phycisphaerae	o:WD2101_soil_group	NA	NA	OTU_1019
k:Bacteria	p:Proteobacteria	c:Gammaproteobacteria	o:Xanthomonadales	f:Xanthomonadaceae	g:Lysobacter	OTU_1064
k:Bacteria	p:Proteobacteria	c:Gammaproteobacteria	o:Xanthomonadales	f:Xanthomonadales_Incerta	g:Acidibacter	OTU_1081
k:Bacteria	p:Proteobacteria	c:Gammaproteobacteria	o:Xanthomonadales	f:Xanthomonadales_Incerta	g:Steroidobacter	OTU_1102
k:Bacteria	p:Proteobacteria	c:Gammaproteobacteria	o:Xanthomonadales	f:Xanthomonadaceae	g:Dyella	OTU_1224
k:Bacteria	p:Proteobacteria	c:Gammaproteobacteria	o:Xanthomonadales	f:Xanthomonadaceae	NA	OTU_1431
k:Bacteria	p:Proteobacteria	c:Gammaproteobacteria	o:Xanthomonadales	f:Xanthomonadaceae	g:Thermomonas	OTU_1804
k:Bacteria	p:Proteobacteria	c:Gammaproteobacteria	o:Xanthomonadales	f:Xanthomonadales_Incerta	g:Steroidobacter	OTU_223
k:Bacteria	p:Proteobacteria	c:Gammaproteobacteria	o:Xanthomonadales	f:Xanthomonadaceae	g:Stenotrophomonas	OTU_308
k:Bacteria	p:Proteobacteria	c:Gammaproteobacteria	o:Xanthomonadales	f:Xanthomonadaceae	NA	OTU_349
k:Bacteria	p:Proteobacteria	c:Gammaproteobacteria	o:Xanthomonadales	f:Xanthomonadaceae	g:Lysobacter	OTU_431
k:Bacteria	p:Proteobacteria	c:Gammaproteobacteria	o:Xanthomonadales	f:Xanthomonadales_Incerta	g:Steroidobacter	OTU_5163
k:Bacteria	p:Proteobacteria	c:Gammaproteobacteria	o:Xanthomonadales	f:Xanthomonadaceae	g:Lysobacter	OTU_525
k:Bacteria	p:Proteobacteria	c:Gammaproteobacteria	o:Xanthomonadales	f:Xanthomonadaceae	g:Arenimonas	OTU_528
k:Bacteria	p:Proteobacteria	c:Gammaproteobacteria	o:Xanthomonadales	f:Xanthomonadaceae	g:Stenotrophomonas	OTU_571
k:Bacteria	p:Proteobacteria	c:Gammaproteobacteria	o:Xanthomonadales	f:Xanthomonadaceae	g:Thermomonas	OTU_616
k:Bacteria	p:Proteobacteria	c:Gammaproteobacteria	o:Xanthomonadales	f:Xanthomonadales_Incerta	g:Steroidobacter	OTU_934
k:Bacteria	NA	NA	NA	NA	NA	OTU_10820
k:Bacteria	p:Actinobacteria	c:Actinobacteria	NA	NA	NA	OTU_1103
k:Archaea	p:Thaumarchaeota	c:Soil_Crenarchaeotic_(	NA	NA	NA	OTU_115
k:Bacteria	p:Actinobacteria	c:Actinobacteria	NA	NA	NA	OTU_1162
k:Bacteria	NA	NA	NA	NA	NA	OTU_122
k:Bacteria	p:Actinobacteria	c:Actinobacteria	NA	NA	NA	OTU_1255
k:Bacteria	p:Proteobacteria	NA	NA	NA	NA	OTU_1311
k:Bacteria	p:Actinobacteria	c:Actinobacteria	NA	NA	NA	OTU_1409
k:Bacteria	NA	NA	NA	NA	NA	OTU_1440
k:Bacteria	p:Bacteroidetes	c:Bacteroidetes_VC2.1_	NA	NA	NA	OTU_1705
k:Bacteria	p:Tenericutes	c:Bacilli	NA	NA	NA	OTU_1760

k:Bacteria	p:Saccharibacteria	NA	NA	NA	NA	OTU_1803
k:Bacteria	p:Chloroflexi	c:S085	NA	NA	NA	OTU_1818
k:Bacteria	p:Actinobacteria	c:Actinobacteria	NA	NA	NA	OTU_189
k:Bacteria	p:Actinobacteria	c:Actinobacteria	NA	NA	NA	OTU_2001
k:Bacteria	p:Chloroflexi	c:Gitt-GS-136	NA	NA	NA	OTU_216
k:Bacteria	p:Proteobacteria	c:Alphaproteobacteria	NA	NA	NA	OTU_231
k:Bacteria	p:Actinobacteria	c:Actinobacteria	NA	NA	NA	OTU_252
k:Bacteria	p:Proteobacteria	NA	NA	NA	NA	OTU_267
k:Bacteria	p:Actinobacteria	c:Actinobacteria	NA	NA	NA	OTU_272
k:Bacteria	p:Saccharibacteria	NA	NA	NA	NA	OTU_426
k:Bacteria	p:Actinobacteria	c:Actinobacteria	NA	NA	NA	OTU_4694
k:Bacteria	p:Chloroflexi	NA	NA	NA	NA	OTU_483
k:Bacteria	p:Chloroflexi	c:TK10	NA	NA	NA	OTU_507
k:Bacteria	p:Actinobacteria	c:Actinobacteria	NA	NA	NA	OTU_508
k:Bacteria	p:Proteobacteria	c:Alphaproteobacteria	NA	NA	NA	OTU_541
k:Bacteria	p:Chloroflexi	c:Gitt-GS-136	NA	NA	NA	OTU_547
k:Bacteria	p:Armatimonadetes	NA	NA	NA	NA	OTU_573
k:Bacteria	p:Chloroflexi	c:KD4-96	NA	NA	NA	OTU_619
k:Bacteria	p:Actinobacteria	c:Actinobacteria	NA	NA	NA	OTU_677
NA	NA	NA	NA	NA	NA	OTU_705
k:Bacteria	p:Proteobacteria	c:Betaproteobacteria	NA	NA	NA	OTU_728
k:Bacteria	p:Actinobacteria	c:Actinobacteria	NA	NA	NA	OTU_78
NA	NA	NA	NA	NA	NA	OTU_846
NA	NA	NA	NA	NA	NA	OTU_859
k:Bacteria	p:Verrucomicrobia	c:OPB35_soil_group	NA	NA	NA	OTU_873
k:Bacteria	p:Chloroflexi	c:JG37-AG-4	NA	NA	NA	OTU_950
k:Bacteria	p:Armatimonadetes	NA	NA	NA	NA	OTU_9668
k:Bacteria	NA	NA	NA	NA	NA	OTU_992

Table S9. Kruskal-Wallis test/Dunn test of the spores-associated bacterial OTUs enrichment in spores compared with soil, rhizosphere, and root endosphere.

Kruskal-Wallis rank sum test

**16SrRNA-V4**	rhiz vs spo	re vs spo	s vs spo
o:Acidimicrobiales	0.055	**0.038**	0.118
o:Acidobacteriales	**0.032**	0.517	0.084
o:Actinomycetales	**0.002**	**0.005**	**0.011**
o:Aeromonadales	**0.006**	**0.007**	**0.008**
o:Anaerolineales	**0.025**	0.634	0.826
o:Bacillales	0.747	0.167	0.621
o:Bacteroidales	**0.0001**	**0.0002**	**0.001**
o:Burkholderiales	0.090	0.143	0.071
o:Caldilineales	**0.039**	0.054	0.725
o:Campylobacterales	1.000	1.000	1.000
o:Catenulisporales	0.762	0.814	0.144
o:Caulobacterales	0.936	0.656	0.733
o:Cellvibrionales	0.485	0.576	0.764
o:Chlamydiales	0.397	0.623	0.516
o:Chlorobiales	0.050	1.000	1.000
o:Chloroflexales	0.033	0.136	**0.005**
o:Chromatiales	0.787	0.024	0.125
o:Chthoniobacterales	0.020	0.713	**0.008**
o:Clostridiales	**0.003**	**0.001**	**0.003**
o:Corynebacteriales	1.000	1.000	1.000
o:Cytophagales	0.114	0.936	0.200
o:Deinococcales	0.081	1.000	0.948
o:Desulfobacterales	0.254	0.609	**0.014**
o:Desulfurellales	0.554	1.000	**0.006**
o:Enterobacteriales	0.100	0.632	0.094
o:Euzebyales	**0.006**	0.344	0.237
o:Fibrobacterales	0.025	1.000	1.000
o:Flavobacteriales	0.114	**0.023**	**0.004**
o:Frankiales	**0.027**	0.733	**0.008**
o:Fusobacteriales	**0.001**	**0.001**	**0.001**
o:Gaiellales	**0.015**	0.631	**0.004**
o:Gemmatimonadales	**0.003**	0.253	**0.014**
o:Kallotenuales	**0.012**	0.330	**0.017**
o:Kineosporiales	**0.025**	0.753	**0.007**
o:Ktedonobacterales	0.083	0.257	0.201
o:Lactobacillales	**0.006**	**0.007**	**0.008**
o:Legionellales	0.100	1.000	1.000
o:Methylophilales	0.636	1.000	0.496
o:Micrococcales	0.909	0.092	0.904
o:Micromonosporales	**0.010**	0.055	0.346

o:Mycoplasmatales	**0.011**	**0.014**	**0.044**
o:Myxococcales	0.970	0.398	0.494
o:Neisseriales	**0.0001**	**0.0002**	**0.001**
o:Nitriliruptorales	0.603	0.580	0.566
o:Nitrosomonadales	**0.031**	0.958	0.094
o:Nitrospirales	**0.011**	0.630	**0.015**
o:Oceanospirillales	**0.010**	0.260	1.000
o:Opitutales	**0.013**	**0.027**	0.571
o:Order_Incertae_Sed	**0.024**	1.000	1.000
o:Pasteurellales	**0.004**	0.002	0.064
o:Phycisphaerales	0.189	0.523	0.541
o:Planctomycetales	**0.003**	0.253	**0.016**
o:Propionibacteriales	0.074	0.902	0.738
o:Pseudomonadales	0.320	0.383	0.076
o:Pseudonocardiales	0.277	0.254	0.377
o:Rhizobiales	0.080	**0.003**	0.309
o:Rhodobacterales	0.615	0.666	0.619
o:Rhodocyclales	0.464	0.385	0.766
o:Rhodospirillales	0.152	0.174	0.246
o:Rickettsiales	0.009	0.746	0.065
o:Rubrobacterales	**0.016**	0.631	**0.004**
o:Selenomonadales	**0.001**	**0.001**	**0.001**
o:Solirubrobacterales	**0.007**	0.229	**0.003**
o:Sphaerobacterales	**0.010**	1.000	0.139
o:Sphingobacteriales	**0.006**	0.114	0.224
o:Sphingomonadales	0.012	0.080	0.250
o:Streptomycetales	0.099	0.492	0.572
o:Streptosporangiales	0.794	0.808	0.625
o:Subgroup_10	0.362	1.000	**0.008**
o:Subgroup_3	**0.002**	0.301	0.055
o:Subgroup_4	**0.006**	0.253	**0.002**
o:SubsectionI	1.000	1.000	0.111
o:SubsectionIII	0.194	1.000	**0.016**
o:Unknown_Order	**0.026**	0.666	**0.033**
o:Verrucomicrobiales	0.147	**0.002**	0.539
o:Xanthomonadales	1.000	1.000	1.000

Table S10. PERMANOVA analyses of the microbial communities associated with *A. tequilana*, *A. salmiana*, and *M. geometrizans* growing in the same arid soil for 12 months. All factors and their interactions were considered, but only those being significant $P \leq 0.05$) are displayed.

AMF and AMF spore-associated bacteria, respectively. NDMS shown in Figure 2.

Factor				Factor			
**AMF**	R^2	F	*P*	**AMF spore-associated bacteria**	R^2	F	*P*
plant.compartment	0.376	7.49	**0.001**	plant.compartment	0.576	14.31	**0.001**
plant.species	0.340	10.17	**0.001**	plant.species	0.132	4.917	**0.001**
plant.compartment:plant.species	0.067	1.00	0.461	plant.compartment:plant.species	0.132	2.458	**0.004**
Residual	0.217			Residual	0.161		
Total	1.000			Total	1		

Global fungal and bacterial communities, respectively. NDMS shown in Figure S5.

Factor				Factor			
**All fungi (ITS2)**	R^2	F	*P*	**16S rRNA-V4**	R^2	F	*P*
plant.compartment	0.534	11.41	**0.001**	plant.compartment	0.657	21.72	**0.001**
plant.species	0.141	4.53	**0.001**	plant.species	0.123	6.10	**0.001**
plant.compartment:plant.species	0.121	1.95	**0.004**	plant.compartment:plant.species	0.099	2.46	**0.005**
Residual	0.203			Residual	0.121		
Total	1.000			Total	1		

Table S11. Kruskal-Wallis/Dunn test of richness, Shannon index and distance to centroid by plant compartment and plant species of agaves and cacti (AMF dataset as shown in Fig. 2a-d). Black numbers showed a significant differences.

Kruskal-Wallis rank sum test
data: richness by plant.compartment
Kruskal-Wallis chi-squared = 13.064, df = 3, p-value = 0.0045

Comparison	Z	p.unadj	p.adj
rhizosphere - root endosphere	-0.1433	0.8861	0.8861
rhizosphere - soil	-2.2608	0.0238	0.0713
rhizosphere - spores	2.1625	0.0306	**0.0459**
root endosphere - soil	-2.1234	0.0337	**0.0405**
root endosphere - spores	2.2323	0.0256	0.0512
soil - spores	3.6116	0.0003	**0.0018**

Kruskal-Wallis rank sum test
data: shannon by plant.compartment
Kruskal-Wallis chi-squared = 9.9303, df = 3, p-value = 0.01917

Comparison	Z	p.unadj	p.adj
rhizosphere - root endosphere	-0.620	0.536	0.536
rhizosphere - soil	-1.843	0.065	0.098
rhizosphere - spores	1.917	0.055	0.111
root endosphere - soil	-1.370	0.171	0.205
root endosphere - spores	2.332	0.020	0.059
soil - spores	3.070	0.002	0.013

Kruskal-Wallis rank sum test
data: distance_to_centroid by plant.compartment
Kruskal-Wallis chi-squared = 7.1138, df = 3, p-value = 0.06836

Comparison	Z	p.unadj	p.adj
rhizosphere - root endosphere	0.965	0.335	0.401
rhizosphere - soil	1.818	0.069	0.207
rhizosphere - spores	2.334	0.020	0.117
root endosphere - soil	1.098	0.272	0.408
root endosphere - spores	1.606	0.108	0.216
soil - spores	0.421	0.673	0.673

Kruskal-Wallis rank sum test
data: richness by plant.species
Kruskal-Wallis chi-squared = 14.061, df = 3, p-value = 0.002824

Comparison	Z	p.unadj	p.adj
Agave.tequilana-Agave.salmiana	1.2304	0.2185	0.2185
Agave.tequilana-Myrtillocactus.geometrizans	1.2304	0.2185	0.2622
Agave.tequilana- soil	-2.3979	0.0165	**0.0330**
Agave.salmiana-Myrtillocactus.geometrizans	2.5614	0.0104	**0.0313**
Agave.salmiana -soil	-1.4651	0.1429	0.2143
Myrtillocactus.geometrizans- soil	-3.4491	0.0006	**0.0034**

Kruskal-Wallis rank sum test
data: shannon by plant.species
Kruskal-Wallis chi-squared = 11.769, df = 3, p-value = 0.008219

Comparison	Z	p.unadj	p.adj
Agave.tequilana-Agave.salmiana	0.91494	0.36022	0.36022
Agave.tequilana-Myrtillocactus.geometrizans	1.65951	0.09701	0.19403
Agave.tequilana- soil	-1.63336	0.10239	0.15359
Agave.salmiana-Myrtillocactus.geometrizans	2.67958	0.00737	**0.02211**
Agave.salmiana -soil	-0.93605	0.34925	0.41910
Myrtillocactus.geometrizans- soil	-3.01164	0.00260	**0.01559**

Kruskal-Wallis rank sum test
data: distance_to_centroid by plant.specie
Kruskal-Wallis chi-squared = 8.0647, df = 3, p-value = 0.04469

Comparison	Z	p.unadj	p.adj
Agave.tequilana-Agave.salmiana	-0.7383	0.4604	0.5524
Agave.tequilana-Myrtillocactus.geometrizans	-1.6469	0.0996	0.1992
Agave.tequilana- soil	0.8688	0.3850	0.5774
Agave.salmiana-Myrtillocactus.geometrizans	-2.4825	0.0130	0.0783
Agave.salmiana -soil	0.2951	0.7679	0.7679
Myrtillocactus.geometrizans- soil	2.2180	0.0266	0.0797

Kruskal-Wallis/Dunn test of richness, Shannon index and distance to centroid by plant compartment and plant species of agaves and cacti (spores-associated bacteria dataset as shown in Fig. 2e-h).
Black numbers showed a significant differences.

Kruskal-Wallis rank sum test
data: richness by plant.compartment
Kruskal-Wallis chi-squared = 17.145, df = 3, p-value = 0.0006598

Comparison	Z	p.unadj	p.adj
rhizosphere - root endosphere	3.0415	0.0024	**0.0071**
rhizosphere - soil	-1.2795	0.2007	0.2409
rhizosphere - spores	1.7534	0.0795	0.1193
root endosphere - soil	-3.5258	0.0004	**0.0025**
root endosphere - spores	-0.4929	0.6221	0.6221
soil - spores	2.5148	0.0119	**0.0238**

Kruskal-Wallis rank sum test
data: shannon by plant.compartment
Kruskal-Wallis chi-squared = 15.773, df = 3, p-value = 0.001262

Comparison	Z	p.unadj	p.adj
rhizosphere - root endosphere	2.8490	0.0044	**0.0132**
rhizosphere - soil	-0.9952	0.3196	0.3836
rhizosphere - spores	2.2652	0.0235	**0.0352**
root endosphere - soil	-3.0993	0.0019	**0.0116**
root endosphere - spores	0.1611	0.8720	0.8720
soil - spores	2.7034	0.0069	**0.0137**

Kruskal-Wallis rank sum test
data: distance_to_centroid by plant.compartment
Kruskal-Wallis chi-squared = 10.563, df = 3, p-value = 0.01434

Comparison	Z	p.unadj	p.adj
rhizosphere - root endosphere	-1.7710	0.0766	0.1531
rhizosphere - soil	1.5259	0.1270	0.1524
rhizosphere - spores	-1.5828	0.1135	0.1702
root endosphere - soil	2.8339	0.0046	0.0276
root endosphere - spores	-0.2749	0.7834	0.7834
soil - spores	-2.5776	0.0099	0.0298

Kruskal-Wallis rank sum test
data: richness by plant.species
Kruskal-Wallis chi-squared = 9.3823, df = 3, p-value = 0.02462

Comparison	Z	p.unadj	p.adj
Agave.tequilana-Agave.salmiana	0.903	0.367	0.440
Agave.tequilana-Myrtillocactus.geometrizans	0.527	0.598	0.598
Agave.tequilana- soil	-2.550	0.011	**0.032**
Agave.salmiana-Myrtillocactus.geometrizans	1.378	0.168	0.252
Agave.salmiana -soil	-1.779	0.075	0.151
Myrtillocactus.geometrizans- soil	-2.904	0.004	**0.022**

Kruskal-Wallis rank sum test
data: shannon by plant.species
Kruskal-Wallis chi-squared = 8.2326, df = 3, p-value = 0.04144

Comparison	Z	p.unadj	p.adj
Agave.tequilana-Agave.salmiana	0.455	0.649	0.649
Agave.tequilana-Myrtillocactus.geometrizans	1.068	0.286	0.343
Agave.tequilana- soil	-2.040	0.041	0.124
Agave.salmiana-Myrtillocactus.geometrizans	1.467	0.142	0.214
Agave.salmiana -soil	-1.633	0.102	0.205
Myrtillocactus.geometrizans- soil	-2.831	0.005	0.028

Kruskal-Wallis rank sum test
data: distance_to_centroid by plant.specie
Kruskal-Wallis chi-squared = 6.7527, df = 3, p-value = 0.08021

Comparison	Z	p.unadj	p.adj
Agave.tequilana-Agave.salmiana	0.1054	0.9160	0.9160
Agave.tequilana-Myrtillocactus.geometrizans	0.4482	0.6540	0.7848
Agave.tequilana- soil	2.3698	0.0178	0.0534
Agave.salmiana-Myrtillocactus.geometrizans	0.5335	0.5937	0.8906
Agave.salmiana -soil	2.3956	0.0166	0.0995
Myrtillocactus.geometrizans- soil	1.9601	0.0500	0.1000

Table S12. List of the MRE and BRE OTUs found in this study.

domain	phylum	class	order	family	genus	otu.id
k:Bacteria	p:Proteobacteria	c:Betaproteobacteria	o:Burkholderiales	f:Comamonadaceae	NA	OTU_10543
k:Bacteria	p:Proteobacteria	c:Betaproteobacteria	o:Burkholderiales	f:Comamonadaceae	NA	OTU_1092
k:Bacteria	p:Proteobacteria	c:Betaproteobacteria	o:Burkholderiales	f:Comamonadaceae	NA	OTU_116
k:Bacteria	p:Proteobacteria	c:Betaproteobacteria	o:Burkholderiales	f:Oxalobacteraceae	g:Massilia	OTU_15
k:Bacteria	p:Proteobacteria	c:Betaproteobacteria	o:Burkholderiales	f:Comamonadaceae	NA	OTU_201
k:Bacteria	p:Proteobacteria	c:Betaproteobacteria	o:Burkholderiales	f:Burkholderiaceae	g:Limnobacter	OTU_204
k:Bacteria	p:Proteobacteria	c:Betaproteobacteria	o:Burkholderiales	f:Comamonadaceae	g:Schlegelella	OTU_2313
k:Bacteria	p:Proteobacteria	c:Betaproteobacteria	o:Burkholderiales	f:Comamonadaceae	NA	OTU_24
k:Bacteria	p:Proteobacteria	c:Betaproteobacteria	o:Burkholderiales	f:Burkholderiaceae	g:Candidatus_Glomeribacter	OTU_2447
k:Bacteria	p:Proteobacteria	c:Betaproteobacteria	o:Burkholderiales	f:Comamonadaceae	NA	OTU_3451
k:Bacteria	p:Proteobacteria	c:Betaproteobacteria	o:Burkholderiales	f:Oxalobacteraceae	g:Noviherbaspirillum	OTU_36
k:Bacteria	p:Proteobacteria	c:Betaproteobacteria	o:Burkholderiales	f:Comamonadaceae	NA	OTU_3674
k:Bacteria	p:Proteobacteria	c:Betaproteobacteria	o:Burkholderiales	f:Comamonadaceae	NA	OTU_461
k:Bacteria	p:Proteobacteria	c:Betaproteobacteria	o:Burkholderiales	f:Oxalobacteraceae	g:Massilia	OTU_481
k:Bacteria	p:Proteobacteria	c:Betaproteobacteria	o:Burkholderiales	f:Burkholderiaceae	g:Ralstonia	OTU_516
k:Bacteria	p:Proteobacteria	c:Betaproteobacteria	o:Burkholderiales	f:Comamonadaceae	NA	OTU_544
k:Bacteria	p:Proteobacteria	c:Betaproteobacteria	o:Burkholderiales	f:Burkholderiaceae	g:Candidatus_Glomeribacter	OTU_568
k:Bacteria	p:Proteobacteria	c:Betaproteobacteria	o:Burkholderiales	f:Comamonadaceae	NA	OTU_6198
k:Bacteria	p:Proteobacteria	c:Betaproteobacteria	o:Burkholderiales	f:Comamonadaceae	NA	OTU_6345
k:Bacteria	p:Proteobacteria	c:Betaproteobacteria	o:Burkholderiales	f:Comamonadaceae	NA	OTU_7468
k:Bacteria	p:Proteobacteria	c:Betaproteobacteria	o:Burkholderiales	f:Burkholderiaceae	g:Burkholderia	OTU_79
k:Bacteria	p:Proteobacteria	c:Betaproteobacteria	o:Burkholderiales	f:Burkholderiaceae	g:Burkholderia	OTU_82
k:Bacteria	p:Proteobacteria	c:Betaproteobacteria	o:Burkholderiales	f:Comamonadaceae	NA	OTU_939
k:Bacteria	p:Tenericutes	c:Mollicutes	o:Mycoplasmatales	f:Mycoplasmataceae	g:Candidatus_Moeniiplasma	OTU_1023
k:Bacteria	p:Tenericutes	c:Mollicutes	o:Mycoplasmatales	f:Mycoplasmataceae	g:Candidatus_Moeniiplasma	OTU_108
k:Bacteria	p:Tenericutes	c:Mollicutes	o:Mycoplasmatales	f:Mycoplasmataceae	g:Candidatus_Moeniiplasma	OTU_109
k:Bacteria	p:Tenericutes	c:Mollicutes	o:Mycoplasmatales	f:Mycoplasmataceae	g:Candidatus_Moeniiplasma	OTU_112
k:Bacteria	p:Tenericutes	c:Mollicutes	o:Mycoplasmatales	f:Mycoplasmataceae	g:Candidatus_Moeniiplasma	OTU_1184
k:Bacteria	p:Tenericutes	c:Mollicutes	o:Mycoplasmatales	f:Mycoplasmataceae	g:Candidatus_Moeniiplasma	OTU_119
k:Bacteria	p:Tenericutes	c:Mollicutes	o:Mycoplasmatales	f:Mycoplasmataceae	g:Candidatus_Moeniiplasma	OTU_11971

k:Bacteria	p:Tenericutes	c:Mollicutes	o:Mycoplasmatales	f:Mycoplasmataceae	g:Candidatus_Moeniiplasma	OTU_1199
k:Bacteria	p:Tenericutes	c:Mollicutes	o:Mycoplasmatales	f:Mycoplasmataceae	g:Candidatus_Moeniiplasma	OTU_1262
k:Bacteria	p:Tenericutes	c:Mollicutes	o:Mycoplasmatales	f:Mycoplasmataceae	g:Candidatus_Moeniiplasma	OTU_1263
k:Bacteria	p:Tenericutes	c:Mollicutes	o:Mycoplasmatales	f:Mycoplasmataceae	g:Candidatus_Moeniiplasma	OTU_14920
k:Bacteria	p:Tenericutes	c:Mollicutes	o:Mycoplasmatales	f:Mycoplasmataceae	g:Candidatus_Moeniiplasma	OTU_1497
k:Bacteria	p:Tenericutes	c:Mollicutes	o:Mycoplasmatales	f:Mycoplasmataceae	g:Candidatus_Moeniiplasma	OTU_150
k:Bacteria	p:Tenericutes	c:Mollicutes	o:Mycoplasmatales	f:Mycoplasmataceae	g:Candidatus_Moeniiplasma	OTU_151
k:Bacteria	p:Tenericutes	c:Mollicutes	o:Mycoplasmatales	f:Mycoplasmataceae	g:Candidatus_Moeniiplasma	OTU_1536
k:Bacteria	p:Tenericutes	c:Mollicutes	o:Mycoplasmatales	f:Mycoplasmataceae	g:Candidatus_Moeniiplasma	OTU_1631
k:Bacteria	p:Tenericutes	c:Mollicutes	o:Mycoplasmatales	f:Mycoplasmataceae	g:Candidatus_Moeniiplasma	OTU_1649
k:Bacteria	p:Tenericutes	c:Mollicutes	o:Mycoplasmatales	f:Mycoplasmataceae	g:Candidatus_Moeniiplasma	OTU_1799
k:Bacteria	p:Tenericutes	c:Mollicutes	o:Mycoplasmatales	f:Mycoplasmataceae	g:Candidatus_Moeniiplasma	OTU_18400
k:Bacteria	p:Tenericutes	c:Mollicutes	o:Mycoplasmatales	f:Mycoplasmataceae	g:Candidatus_Moeniiplasma	OTU_1879
k:Bacteria	p:Tenericutes	c:Mollicutes	o:Mycoplasmatales	f:Mycoplasmataceae	g:Candidatus_Moeniiplasma	OTU_2083
k:Bacteria	p:Tenericutes	c:Mollicutes	o:Mycoplasmatales	f:Mycoplasmataceae	g:Candidatus_Moeniiplasma	OTU_21120
k:Bacteria	p:Tenericutes	c:Mollicutes	o:Mycoplasmatales	f:Mycoplasmataceae	g:Candidatus_Moeniiplasma	OTU_2126
k:Bacteria	p:Tenericutes	c:Mollicutes	o:Mycoplasmatales	f:Mycoplasmataceae	g:Candidatus_Moeniiplasma	OTU_2276
k:Bacteria	p:Tenericutes	c:Mollicutes	o:Mycoplasmatales	f:Mycoplasmataceae	g:Candidatus_Moeniiplasma	OTU_2322
k:Bacteria	p:Tenericutes	c:Mollicutes	o:Mycoplasmatales	f:Mycoplasmataceae	g:Candidatus_Moeniiplasma	OTU_244
k:Bacteria	p:Tenericutes	c:Mollicutes	o:Mycoplasmatales	f:Mycoplasmataceae	g:Candidatus_Moeniiplasma	OTU_2445
k:Bacteria	p:Tenericutes	c:Mollicutes	o:Mycoplasmatales	f:Mycoplasmataceae	g:Candidatus_Moeniiplasma	OTU_2446
k:Bacteria	p:Tenericutes	c:Mollicutes	o:Mycoplasmatales	f:Mycoplasmataceae	g:Candidatus_Moeniiplasma	OTU_2489
k:Bacteria	p:Tenericutes	c:Mollicutes	o:Mycoplasmatales	f:Mycoplasmataceae	g:Candidatus_Moeniiplasma	OTU_25136
k:Bacteria	p:Tenericutes	c:Mollicutes	o:Mycoplasmatales	f:Mycoplasmataceae	g:Candidatus_Moeniiplasma	OTU_257
k:Bacteria	p:Tenericutes	c:Mollicutes	o:Mycoplasmatales	f:Mycoplasmataceae	g:Candidatus_Moeniiplasma	OTU_27
k:Bacteria	p:Tenericutes	c:Mollicutes	o:Mycoplasmatales	f:Mycoplasmataceae	g:Candidatus_Moeniiplasma	OTU_2715
k:Bacteria	p:Tenericutes	c:Mollicutes	o:Mycoplasmatales	f:Mycoplasmataceae	g:Candidatus_Moeniiplasma	OTU_2826
k:Bacteria	p:Tenericutes	c:Mollicutes	o:Mycoplasmatales	f:Mycoplasmataceae	g:Candidatus_Moeniiplasma	OTU_295
k:Bacteria	p:Tenericutes	c:Mollicutes	o:Mycoplasmatales	f:Mycoplasmataceae	g:Candidatus_Moeniiplasma	OTU_3014
k:Bacteria	p:Tenericutes	c:Mollicutes	o:Mycoplasmatales	f:Mycoplasmataceae	g:Candidatus_Moeniiplasma	OTU_3062
k:Bacteria	p:Tenericutes	c:Mollicutes	o:Mycoplasmatales	f:Mycoplasmataceae	g:Candidatus_Moeniiplasma	OTU_3159
k:Bacteria	p:Tenericutes	c:Mollicutes	o:Mycoplasmatales	f:Mycoplasmataceae	g:Candidatus_Moeniiplasma	OTU_3160
k:Bacteria	p:Tenericutes	c:Mollicutes	o:Mycoplasmatales	f:Mycoplasmataceae	g:Candidatus_Moeniiplasma	OTU_3209

k:Bacteria	p:Tenericutes	c:Mollicutes	o:Mycoplasmatales	f:Mycoplasmataceae	g:Candidatus_Moeniiplasma	OTU_3239
k:Bacteria	p:Tenericutes	c:Mollicutes	o:Mycoplasmatales	f:Mycoplasmataceae	g:Candidatus_Moeniiplasma	OTU_350
k:Bacteria	p:Tenericutes	c:Mollicutes	o:Mycoplasmatales	f:Mycoplasmataceae	g:Candidatus_Moeniiplasma	OTU_354
k:Bacteria	p:Tenericutes	c:Mollicutes	o:Mycoplasmatales	f:Mycoplasmataceae	g:Candidatus_Moeniiplasma	OTU_3722
k:Bacteria	p:Tenericutes	c:Mollicutes	o:Mycoplasmatales	f:Mycoplasmataceae	g:Candidatus_Moeniiplasma	OTU_4062
k:Bacteria	p:Tenericutes	c:Mollicutes	o:Mycoplasmatales	f:Mycoplasmataceae	g:Candidatus_Moeniiplasma	OTU_4064
k:Bacteria	p:Tenericutes	c:Mollicutes	o:Mycoplasmatales	f:Mycoplasmataceae	g:Candidatus_Moeniiplasma	OTU_4065
k:Bacteria	p:Tenericutes	c:Mollicutes	o:Mycoplasmatales	f:Mycoplasmataceae	g:Candidatus_Moeniiplasma	OTU_42
k:Bacteria	p:Tenericutes	c:Mollicutes	o:Mycoplasmatales	f:Mycoplasmataceae	g:Candidatus_Moeniiplasma	OTU_52
k:Bacteria	p:Tenericutes	c:Mollicutes	o:Mycoplasmatales	f:Mycoplasmataceae	g:Candidatus_Moeniiplasma	OTU_526
k:Bacteria	p:Tenericutes	c:Mollicutes	o:Mycoplasmatales	f:Mycoplasmataceae	g:Candidatus_Moeniiplasma	OTU_533
k:Bacteria	p:Tenericutes	c:Mollicutes	o:Mycoplasmatales	f:Mycoplasmataceae	g:Candidatus_Moeniiplasma	OTU_5576
k:Bacteria	p:Tenericutes	c:Mollicutes	o:Mycoplasmatales	f:Mycoplasmataceae	g:Candidatus_Moeniiplasma	OTU_57
k:Bacteria	p:Tenericutes	c:Mollicutes	o:Mycoplasmatales	f:Mycoplasmataceae	g:Candidatus_Moeniiplasma	OTU_578
k:Bacteria	p:Tenericutes	c:Mollicutes	o:Mycoplasmatales	f:Mycoplasmataceae	g:Candidatus_Moeniiplasma	OTU_6038
k:Bacteria	p:Tenericutes	c:Mollicutes	o:Mycoplasmatales	f:Mycoplasmataceae	g:Candidatus_Moeniiplasma	OTU_6040
k:Bacteria	p:Tenericutes	c:Mollicutes	o:Mycoplasmatales	f:Mycoplasmataceae	g:Candidatus_Moeniiplasma	OTU_6506
k:Bacteria	p:Tenericutes	c:Mollicutes	o:Mycoplasmatales	f:Mycoplasmataceae	g:Candidatus_Moeniiplasma	OTU_6507
k:Bacteria	p:Tenericutes	c:Mollicutes	o:Mycoplasmatales	f:Mycoplasmataceae	g:Candidatus_Moeniiplasma	OTU_672
k:Bacteria	p:Tenericutes	c:Mollicutes	o:Mycoplasmatales	f:Mycoplasmataceae	g:Candidatus_Moeniiplasma	OTU_685
k:Bacteria	p:Tenericutes	c:Mollicutes	o:Mycoplasmatales	f:Mycoplasmataceae	g:Candidatus_Moeniiplasma	OTU_691
k:Bacteria	p:Tenericutes	c:Mollicutes	o:Mycoplasmatales	f:Mycoplasmataceae	g:Candidatus_Moeniiplasma	OTU_7233
k:Bacteria	p:Tenericutes	c:Mollicutes	o:Mycoplasmatales	f:Mycoplasmataceae	g:Candidatus_Moeniiplasma	OTU_8537
k:Bacteria	p:Tenericutes	c:Mollicutes	o:Mycoplasmatales	f:Mycoplasmataceae	g:Candidatus_Moeniiplasma	OTU_876
k:Bacteria	p:Tenericutes	c:Mollicutes	o:Mycoplasmatales	f:Mycoplasmataceae	g:Candidatus_Moeniiplasma	OTU_9242
k:Bacteria	p:Tenericutes	c:Mollicutes	o:Mycoplasmatales	f:Mycoplasmataceae	g:Candidatus_Moeniiplasma	OTU_948
k:Bacteria	p:Tenericutes	c:Mollicutes	o:Mycoplasmatales	f:Mycoplasmataceae	g:Candidatus_Moeniiplasma	OTU_989

Table S13. List of the 16S rRNA gene seqences used for phylogenetic analysis included in Figure 3.

Strain ID	Accesion	Fungal Endosymbion	Bacteria order/genus	Origin	References
MRECEUSA	KP763319	*Claroideoglomus etunicatum*	Mycoplasmatales	USA	Toomer *et al.* , 2015
MREGMCAM	KF378701	*Gigaspora margarita*	Mycoplasmatales	Cameroon	Desiro *et al.* , 2014
MREFMUK	FJ984707	*Funneliformis mosseae*	Mycoplasmatales	UK	Naumann *et al.* , 2010
MREFMBOL	FJ984704	*Funneliformis mosseae*	Mycoplasmatales	Bolivia	Naumann *et al.* , 2010
MREA2RI	MW161361	*Rhizophagus irregularis*	Mycoplasmatales	Unknown	Savary *et al.* , 2021
MRELPA8RSP	MW161347	*Rhizophagus sp.*	Mycoplasmatales	Unknown	Savary *et al.* , 2021
MREGVUSA	FJ984723	*Glomus versiforme*	Mycoplasmatales	USA	Naumann *et al.* , 2010
MREGCFIN	FJ984671	*Glomus claroideum*	Mycoplasmatales	Finland	Naumann *et al.* , 2010
MREAPBRA	FJ984639	*Ambispora appendicula*	Mycoplasmatales	Brasil	Naumann *et al.* , 2010
MREAPBRA1	FJ984640	*Ambispora appendicula*	Mycoplasmatales	Brasil	Naumann *et al.* , 2010
MREAFFIN	FJ984643	*Ambispora fennica*	Mycoplasmatales	Finland	Naumann *et al.* , 2010
MRECECL372	KP763334	*Claroideoglomus etunicatum*	Mycoplasmatales	Colombia	Toomer *et al.* , 2015
MRECECL750	*KP763398*	*Cetraspora pellucida*	Mycoplasmatales	Colombia	Toomer *et al.* , 2015
MREDHBR	*KP763368*	*Dentiscutata heterogama*	Mycoplasmatales	Brazil	Toomer *et al.* , 2015
MRECEUSA1	KP763356	*Claroideoglomus etunicatum*	Mycoplasmatales	USA	Toomer *et al.* , 2015
MRECEMX1	KP763359	*Claroideoglomus etunicatum*	Mycoplasmatales	México	Toomer *et al.* , 2015
MRECEMX2	KP763360	*Claroideoglomus etunicatum*	Mycoplasmatales	México	Toomer *et al.* , 2015
MRECEMX3	KP763361	*Claroideoglomus etunicatum*	Mycoplasmatales	México	Toomer *et al.* , 2015
MRECEMX4	KP763362	*Claroideoglomus etunicatum*	Mycoplasmatales	México	Toomer *et al.* , 2015
MRECEMX5	KP763363	*Claroideoglomus etunicatum*	Mycoplasmatales	México	Toomer *et al.* , 2015
MRECEMX6	KP763364	*Claroideoglomus etunicatum*	Mycoplasmatales	México	Toomer *et al.* , 2015
MRECEMX7	KP763365	*Claroideoglomus etunicatum*	Mycoplasmatales	México	Toomer *et al.* , 2015
MRECEMX8	KP763366	*Claroideoglomus etunicatum*	Mycoplasmatales	México	Toomer *et al.* , 2015
MRECEAU401	KP763269	*Claroideoglomus etunicatum*	Mycoplasmatales	Australia	Toomer *et al.* , 2015
MREGCPOL	MT031995	*Glomus claroideum*	Mycoplasmatales	Poland	Okrasinska *et al.* , 2021
MREGCPOL1	MT031997	*Glomus claroideum*	Mycoplasmatales	Poland	Okrasinska *et al.* , 2021
MREDSPPOL	MT032000	*Diversispora* sp.	Mycoplasmatales	Poland	Okrasinska *et al.* , 2021
MRELEUSA	MG052971	*Linnemannia elongata*	Mycoplasmatales	USA	Desiro *et al.* , 2018
MRELEUSA1	MG052974	*Linnemannia elongata*	Mycoplasmatales	USA	Desiro *et al.* , 2018
MREMORAUS	MG052969	Mortierellomycotina fungi	Mycoplasmatales	Australia	Desiro *et al.* , 2018

MREMORAKE	MG052968	Mortierellomycotina fungi	Mycoplasmatales	Kenya	Desiro *et al.* , 2018
MRELTUSA	MG052967	*Lobosporangium transversale*	Mycoplasmatales	USA	Desiro *et al.* , 2018
MREENIT	KM594010	*Endogone* sp.	Mycoplasmatales	Italy	Desiro *et al.* , 2015
MREENMX	KM594009	*Endogone* sp.	Mycoplasmatales	México	Desiro *et al.* , 2015
MREENMX1	KM594011	*Endogone* sp.	Mycoplasmatales	México	Desiro *et al.* , 2015
MREELMX	KM594012	*Endogone lactiflua*	Mycoplasmatales	México	Desiro *et al.* , 2015
MREELUSA	KM594007	*Endogone lactiflua*	Mycoplasmatales	USA	Desiro *et al.* , 2015
BREGMCAM	KF378652	*Gigaspora margarita*	Burkholderiales	Cameroon	Desiro *et al.* , 2014
BREBEG34	X89727	*Gigaspora margarita*	Burkholderiales	New Zeland	Bianciotto *et al.* , 1996
BRESPITA	AJ251635	*Scutellospora persica*	Burkholderiales	Italy	Bianciotto *et al.* , 2000
BREGMUSA	AJ251633	*Gigaspora margarita*	Burkholderiales	USA	Bianciotto *et al.* , 2000
BREMEUSA	MN692880	Mortierellomycotina fungi	Burkholderiales	USA	Desiro *et al.,* 2018
BREAB1JP	NR_149240	*Linnemannia elongata*	Burkholderiales	Japan	Ohshima *et al.* , 2016
BREAM40	MT002703	*Linnemannia* sp.	Burkholderiales	Canada	Okrasinska *et al.* , 2021
BREAM401	MW055768	*Linnemannia* sp.	Burkholderiales	Canada	Okrasinska *et al.* , 2021
BREBM48	MT002704	*Linnemannia* sp.	Burkholderiales	Canada	Okrasinska *et al.* , 2021
BREBMGC110	MT002706	*Linnemannia gamsi*	Burkholderiales	Poland	Okrasinska *et al.* , 2021
BREBM4812	MW055773	*Linnemannia* sp.	Burkholderiales	Canada	Okrasinska *et al.* , 2021
BREAMVJP	MF383419	*M. verticillata*	Burkholderiales	Japan	Takashima *et al.* , 2018
BREAMBJP	MF383418	*M. basiparvispora*	Burkholderiales	Japan	Takashima *et al.* , 2018
BREBMSPJP	MF383425	*Linnemannia* sp.	Burkholderiales	Japan	Takashima *et al.* , 2018
BREBLEJP	AB558493	*Linnemannia elongata*	Burkholderiales	Japan	Takashima *et al.* , 2018
BRECLEJP	MF383450	*Linnemannia elongata*	Burkholderiales	Japan	Takashima *et al.* , 2018
BRECLEJP1	MH760810	*Linnemannia elongata*	Burkholderiales	Japan	Takashima *et al.* , 2018
BRECMVJP	MF383455	*M. verticillata*	Burkholderiales	Japan	Takashima *et al.* , 2018
MRHIJPB1	NR_042393	*Rhizopus microsporus*	Burkholderiales	Japan	Partida-Martinez *et al.* , 2007
MENDMOB1	NR_042584	*Rhizopus microsporus*	Burkholderiales	Mozambique	Partida-Martinez *et al.* , 2007
MSPUKB4	AJ938144	*Rhizopus microsporus*	Burkholderiales	Ukraine	Partida-Martinez *et al.* , 2005
MSPGEOB7	FN186054	*Rhizopus microsporus*	Burkholderiales	Gerogia	Lackner *et al.* , 2009
MSPUSB6	FN186053	*Rhizopus microsporus*	Burkholderiales	USA	Lackner *et al.* , 2009
MSPMX475	OM634668	*Rhizopus delemar* HP475	Burkholderiales	México	Cabrera-Rangel *et al.* , 2022
MSPMX499	OM634667	*Rhizopus microsporus* HP499	Burkholderiales	México	Cabrera-Rangel *et al.* , 2022
BTHAI	NR_027587	NA	*Burkholderia thailandensis*	Thailand	Brett *et al.* , 1998

BPUSA	NR_043553	NA	*Burkholderia pseudomallei*	USA	Glass *et al.* , 2006;
RSPOR	NR_044040	NA	Ralstonia solanacearum	Portugal	NCBI: PRJNA33175
RMUK	NR_025385	NA	Ralstonia mannitolilytica	UK	De Baere *et al.* , 2001;
CINS	NR_178243	NA	*Caballeronia insecticola*	Japan	Kikuchi *et al.* , 2011
PFUN	NR_025058	*Phanerochaete chrysosporium*	*Paraburkholderia fungorum*	NA	Coenye *et al.* , 2001
PCALEUK	NR_025057	NA	*Paraburkholderia caledonica*	UK	Coenye *et al.* , 2001
BRELEPOL	MT002698	*Linnemannia elongata*	Burkholderiales	Poland	Okrasinska *et al.* , 2021
BRELEPOL1	MT002697	*Linnemannia elongata*	Burkholderiales	Poland	Okrasinska *et al.* , 2021
BRELEPOL2	MT002701	*Linnemannia elongata*	Burkholderiales	Poland	Okrasinska *et al.* , 2021
CAMNEC	CP076444	*M. verticillata*	*Ca* . M. necroximicus	NA	Blanchard *et al.* , 1993
CAMSP	CP102085	*M. verticillata*	*Ca.* M. sp.	NA	NCBI
MYCHOM	MYC16SRRNB	NA	*Mycoplasma hominis*	NA	Buttner *et al.* , 2022
MYCPNE	AB680604	NA	Mycoplasma pneumoniae	NA	Buttner *et al.* , 2022

Table S14. Molecular identification of the AMF OTUs obtained in this study and the list of the ITS2 gene sequences used for phylogenetic analysis included in Figure S7.

OTU	Coverage	E-value	Similarity	Best NCBI hit	Accesion
OTU_87_Ambispora	99%	2.00E-173	98.33%	*Ambispora appendicula*	FN547533
OTU_140_Ambispora	99%	8.00E-165	97.21%	Uncultured Ambispora	HF970302
OTU_195_Ambispora	100%	3.00E-158	95.77%	*Ambispora leptoticha*	AB048645
OTU_256_Ambispora	98%	6.00E-160	95.81%	Ambispora appendicula	FN547532
OTU_490_Ambispora	99%	5.00E-123	90.00%	Ambispora fennica	FN547544
OTU_541_Funneliformis	100%	3.00E-171	96.29%	Uncultured Funneliformis	HE775329
OTU_813_Ambispora	100%	1.00E-165	96.66%	Ambispora appendicula	KM083144
OTU_844_Kamienskia	99%	4.00E-165	98.52%	Kamienskia divaricata	KX758123
OTU_1134_Paraglomus	98%	8.00E-165	97.47%	Uncultured Glomeromycota	HM162336
OTU_1278_Paraglomus	100%	0.00E+00	97.20%	Paraglomus sp.	MT765344
OTU_1339_Rhizophagus	100%	3.00E-151	95.93%	Uncultured Glomeromycota	JX276906
OTU_1854_Ambispora	99%	4.00E-154	95.18%	Uncultured Ambispora	HF970302
OTU_2003_Claroideoglomus	99%	0.00E+00	99.18%	Claroideoglomus claroideum	GQ388714
OTU_2069_Rhizophagus	100%	2.00E-158	97.39%	Uncultured Rhizophagus	MF589993
OTU_2383_Glomus	100%	6.00E-160	97.64%	Uncultured Glomus	KM041744
OTU_2665_Rhizophagus	100%	4.00E-162	96.92%	Rhizophagus proliferus	NR_121371
OTU_2726_Rhizophagus	100%	8.00E-152	95.92%	Uncultured Glomeromycota	JX276906
OTU_2797_Glomus	100%	2.00E-152	96.21%	Uncultured Glomus	JN195674
OTU_2872_Pervetustus	100%	3.00E-100	86.00%	Pervetustus simplex	KY630237
OTU_2873_Glomus	100%	6.00E-142	95.00%	Uncultured Glomus	JN195681
OTU_2874_Glomus	100%	5.00E-168	98.53%	Uncultured Glomus	GQ388371
OTU_2877_Glomus	100%	5.00E-168	99.11%	Uncultured Glomus	JN195671
OTU_2986_Paraglomus	100%	1.00E-64	87.61%	Uncultured Glomeromycota	HM162336
OTU_3092_Ambispora	100%	8.00E-152	94.41%	Ambispora leptoticha	AB048653
OTU_3189_Septoglomus	65%	2.00E-32	76.79%	Septoglomus sp.	MT227160

OTU_3453_Glomus	98%	2.00E-171	99.12%	Uncultured Glomus	JN195518
OTU_3995_Rhizoglomus	100%	9.00E-177	99.44%	Rhizoglomus sp.	MT765713
OTU_4262_Glomus	98%	4.00E-114	90%	Uncultured Glomus	JN195554
OTU_4288_Glomus	100%	6.00E-160	97.39%	Uncultured Glomus	MF590001
OTU_4548_Glomus	99%	6.00E-147	95.31%	Uncultured Glomus	GQ388435
OTU_4567_Acaulospora	100%	6.00E-179	98.64%	Uncultured Acaulospora	KM041933
OTU_4854_Glomus	100%	4.00E-168	97.5	Uncultured Glomus	JN195524
OTU_4889_Funneliformis	98%	2.00E-179	98.39%	Funneliformis mosseae	NR_121386
OTU_4899_Glomus	100%	1.00E-179	98.36%	Uncultured Glomus	JN194537
OTU_4902_Rhizophagus	99%	7.00E-172	98.10%	Uncultured Rhizophagus	HG425867
OTU_5326_Glomus	100%	2.00E-154	95.70%	Uncultures Glomus	MF589996
OTU_5387_Glomus	100%	1.00E-124	90.38%	Uncultured Glomus	JN195554
OTU_5830_Kamienskia	100%	6.00E-128	91.55%	Kamienskia divaricata	KX758125
OTU_5940_Glomus	100%	2.00E-152	94.96%	Uncultured Glomus	JN195708
OTU_5943_Glomus	100%	4.00E-174	96.82%	Uncultured Glomus	JN195736
OTU_5952_Glomus	100%	7.00E-157	96.25%	Uncultured Glomeromycota	HM162342
OTU_5955_Glomus	100%	3.00E-154	98.25%	Uncultured Glomus	FJ769323
OTU_6029_Rhizophagus	100%	2.00E-166	98.02%	Rhizophagus irregularis	JF820457
OTU_6031_Rhizoglomus	99%	3.00E-164	97.46%	Rhizoglomus sp.	MT765720
OTU_6050_Claroideoglomus	100%	4.00E-168	96.76%	Uncultured Claroideoglomus	HF970233
OTU_6068_Ambispora	100%	1.00E-155	95.00%	Ambispora appendicula	KM083145
OTU_6070_Acaulospora	100%	1.00E-159	94.69%	Uncultured Acaulospora	FR732068
OTU_6998_Rhizoglomus	100%	1.00E-167	99.41%	Rhizoglomus sp.	MT765664
OTU_7002_Glomus	99%	5.00E-161	96.87%	Uncultured Glomus	JN195697
OTU_7004_Glomus	100%	5.00E-155	96.33%	Uncultured Glomus	HQ917527
OTU_7027_Rhizophagus	100%	1.00E-162	98.04%	Uncultured Glomeraceae	MT227150
OTU_7037_Glomus	100%	4.00E-168	98.04%	Uncultured Glomus	JN194818
OTU_7038_Glomus	100%	4.00E-175	99.44%	Uncultured Glomus	JN195307
OTU_7052_Glomus	99%	7.00E-159	97.38%	Uncultured Glomeromycota	HM162332

OTU_7066_Rhizophagus	100%	1.00E-159	97.08%	Uncultured Glomeromycota	JX276906
OTU_7116_Glomus	100%	4.00E-143	94.46%	Uncultured Glomus	AY236238
OTU_8586_Glomus	100%	1.00E-161	96.42%	Uncultured Glomus	KJ701452
OTU_8633_Glomus	98%	2.00E-147	95%	Uncultured Glomus	JN195707
OTU_8687_Entrophospora	100%	9.00E-69	78.75%	Entrophospora sp.	AY035644
OTU_8729_Paraglomus	98%	0%	99.23%	Paraglomus sp.	MT765702
OTU_8798_Nanoglomus	100%	5.00E-142	92.76%	Nanoglomus sp.	MT765554
OTU_8803_Rhizophagus	100%	1.00E-175	98.36%	Rhizophagus irregularis	FM992379
OTU_8804_Paraglomus	99%	1.00E-114	88.49%	Uncultured Glomeromycota	HM162336
OTU_8848_Rhizophagus	100%	7.00E-134	92.51%	Rhizophagus irregularis	MN388908

(continued) List of the ITS2 gene secuences used for phylogenetic analysis in Figure S7.

Strain ID	Name	Accesion	Origin	Reference
ACENARG	*Acaulospora entreriana E-W5476*	NR_121441	Argentina	Kruger *et al.* , 2012
ACCAUK	*Acaulospora cavernata BEG 33*	NR_121362	UK	Kruger *et al.* , 2012
ARECU	*Archaeospora ecuadoriana* E W5337	NR_168426	Ecuador	Schussler & Walker, 2019
ENSPSWI	*Entrophospora* sp.	AY035644	Switzerland	Jansa *et al.* , 2002
FUNMOSUK	*Funneliformis mosseae BEG 12*	NR_121386	UK	Stockinger *et al.* , 2010
GLOMACUK	*Glomus macrocarpum* E W5581	NR_121448	UK	Kruger *et al.* , 2012
PAOCUSA	*Paraglomus occultum* INVAM IA702	NR_119545	USA	Kruger *et al.* , 2012
REMEGDL	*Redeckera megalocarpum* LIP CL	NR_121292	Guadeloupe	Redecker *et al.* , 2007
RHIPROGDL	*Rhizophagus proliferus* MUCL 41827	NR_121385	Guadeloupe	Kruger *et al.* , 2012
RHIMIMX	*Rhizopus microsporus* HP499	OM677455	México	Cabrera-Rangel *et al.* , 2022
RHDEMX	*Rhizopus delemar* HP475	OM677456	México	Cabrera-Rangel *et al.* , 2022
MUCLUS	*Mucor lusitanicus* CBS108.17	NR_126127	NA	Li *et al.* , 2011
MUCCIR	*Mucor circinelloides* CBS 195.68	NR_126116	NA	Schwarz *et al.* , 2006

Table S15. Network metrics used to generate the co-ocurrence network from the spores.

network	density	diameter	modularity	clustering	av.short.phat	vertex
spores	0.02	8.00	0.52	0.11	3.81	259

edges	p.e.edges	p.p.edges	e.e.edges	pos.edges	neg.edges	total.edges
718	164	493	61	453	265	718

Table S16. Identified nodes and hubs in the co-occurrence network from the spores. Hubs were determined by the values of degree >6 and/or betweenness >750.

d	b/c	domain	phylum	class	order	family	genus	otu.id
7	739	k:Bacteria	p:Tenericutes	c:Bacilli	o:Bacillales	f:Bacillaceae	g:Bacillus	POTU_31
11	1192	k:Bacteria	p:Proteobacteria	c:Betaproteobacteria	o:Burkholderiales	f:Oxalobacteraceae	g:Noviherbaspirillum	POTU_36
8	1165	k:Bacteria	p:Tenericutes	c:Mollicutes	o:Mycoplasmatales	f:Mycoplasmataceae	g:Ca_Moeniiplasma	POTU_57
8	621	k:Bacteria	p:Proteobacteria	c:Alphaproteobacteria	o:Rhizobiales	f:Rhizobiaceae	g:Rhizobium	POTU_58
7	693	k:Bacteria	p:Actinobacteria	c:Actinobacteria	o:Micrococcales	f:Cellulomonadaceae	g:Unidentified	POTU_68
9	787	k:Bacteria	p:Proteobacteria	c:Alphaproteobacteria	o:Caulobacterales	f:Caulobacteraceae	g:Brevundimonas	POTU_90
8	943	k:Bacteria	p:Actinobacteria	c:Actinobacteria	o:Pseudonocardiales	f:Pseudonocardiaceae	g:Pseudonocardia	POTU_101
8	711	k:Bacteria	p:Actinobacteria	c:Actinobacteria	o:Pseudonocardiales	f:Pseudonocardiaceae	g:Unidentified	POTU_105
6	768	k:Bacteria	p:Proteobacteria	c:Alphaproteobacteria	o:Caulobacterales	f:Caulobacteraceae	g:Brevundimonas	POTU_134
7	609	k:Bacteria	p:Gemmatimonadetes	c:Gemmatimonadetes	o:AT425-EubC11	f:Unidentified	g:Unidentified	POTU_169
8	697	k:Bacteria	p:Proteobacteria	c:Gammaproteobacteria	o:Pseudomonadales	f:Pseudomonadaceae	g:Pseudomonas	POTU_202
9	716	k:Bacteria	p:Proteobacteria	c:Deltaproteobacteria	o:Myxococcales	f:Sandaracinaceae	g:Unidentified	POTU_228
6	645	k:Bacteria	p:Actinobacteria	c:Actinobacteria	o:Corynebacteriales	f:Corynebacteriaceae	g:Corynebacterium	POTU_268
7	1336	k:Bacteria	p:Actinobacteria	c:Actinobacteria	o:Micromonosporales	f:Micromonosporaceae	g:Actinoplanes	POTU_388
8	587	k:Bacteria	p:Proteobacteria	c:Alphaproteobacteria	o:Rhodobacterales	f:Rhodobacteraceae	g:Rubellimicrobium	POTU_403
7	676	k:Bacteria	p:Proteobacteria	c:Alphaproteobacteria	o:Rhizobiales	f:Rhizobiaceae	g:Shinella	POTU_501
7	795	k:Bacteria	p:Bacteroidetes	c:Bacteroidia	o:Bacteroidales	f:Porphyromonadaceae	g:Porphyromonas	POTU_511
7	739	k:Bacteria	p:Tenericutes	c:Mollicutes	o:Mycoplasmatales	f:Mycoplasmataceae	g:Ca_Moeniiplasma	POTU_526
9	613	k:Bacteria	p:Tenericutes	c:Mollicutes	o:Mycoplasmatales	f:Mycoplasmataceae	g:Ca_Moeniiplasma	POTU_533
7	576	k:Bacteria	p:Actinobacteria	c:Actinobacteria	o:Micrococcales	f:Micrococcaceae	g:Rothia	POTU_564
10	1092	k:Bacteria	p:Actinobacteria	c:Actinobacteria	o:Actinomycetales	f:Actinomycetaceae	g:Actinomyces	POTU_565
7	741	k:Bacteria	p:Proteobacteria	c:Betaproteobacteria	o:Neisseriales	f:Neisseriaceae	g:Kingella	POTU_765
7	577	k:Bacteria	p:Tenericutes	c:Bacilli	o:Bacillales	f:Bacillaceae	g:Bacillus	POTU_929
8	1073	k:Bacteria	p:Actinobacteria	c:Actinobacteria	o:Unidentified	f:Unidentified	g:Unidentified	POTU_1103
8	726	k:Bacteria	p:Proteobacteria	c:Gammaproteobacteria	o:Pseudomonadales	f:Pseudomonadaceae	g:Pseudomonas	POTU_1112
8	838	k:Bacteria	p:Actinobacteria	c:Actinobacteria	o:Pseudonocardiales	f:Pseudonocardiaceae	g:Unidentified	POTU_1119
9	571	k:Bacteria	p:Actinobacteria	c:Actinobacteria	o:Corynebacteriales	f:Corynebacteriaceae	g:Corynebacterium	POTU_1194
7	640	k:Bacteria	p:Proteobacteria	c:Unidentified	o:Unidentified	f:Unidentified	g:Unidentified	POTU_1311
8	666	k:Bacteria	p:Tenericutes	c:Negativicutes	o:Selenomonadales	f:Veillonellaceae	g:Veillonella	POTU_1456
6	604	k:Bacteria	p:Tenericutes	c:Bacilli	o:Bacillales	f:Planococcaceae	g:Rummeliibacillus	POTU_1663

8	924	k:Bacteria	p:Tenericutes	c:Mollicutes	o:Mycoplasmatales	f:Mycoplasmataceae	g:Ca_Moeniiplasma	POTU_2322
8	736	k:Bacteria	p:Proteobacteria	c:Betaproteobacteria	o:Neisseriales	f:Neisseriaceae	g:Unidentified	POTU_2941
11	824	k:Bacteria	p:Tenericutes	c:Mollicutes	o:Mycoplasmatales	f:Mycoplasmataceae	g:Ca_Moeniiplasma	POTU_3239
8	665	k:Bacteria	p:Tenericutes	c:Bacilli	o:Lactobacillales	f:Streptococcaceae	g:Streptococcus	POTU_3897
7	731	k:Bacteria	p:Tenericutes	c:Mollicutes	o:Mycoplasmatales	f:Mycoplasmataceae	g:Ca_Moeniiplasma	POTU_9242
8	905	k:Bacteria	p:Proteobacteria	c:Gammaproteobacteria	o:Pseudomonadales	f:Pseudomonadaceae	g:Pseudomonas	POTU_25191
6	631	d:Fungi	p:Ascomycota	c:Sordariomycetes	o:Hypocreales	f:Nectriaceae	g:Unidentified	FOTU_12
7	616	d:Fungi	p:Ascomycota	c:Eurotiomycetes	o:Eurotiales	f:Aspergillaceae	g:Penicillium	FOTU_16
6	592	d:Fungi	p:Ascomycota	c:Sordariomycetes	o:Sordariales	f:Chaetomiaceae	g:Unidentified	FOTU_41
6	588	d:Unidentif	p:Unidentified	c:Unidentified	o:Unidentified	f:Unidentified	g:Unidentified	FOTU_139
6	597	d:Unidentif	p:Unidentified	c:Unidentified	o:Unidentified	f:Unidentified	g:Unidentified	FOTU_220
6	570	d:Fungi	p:Glomeromycota	c:Archaeosporomycetes	o:Archaeosporales	f:Ambisporaceae	g:Ambispora	FOTU_490
7	721	d:Fungi	p:Glomeromycota	c:Glomeromycetes	o:Glomerales	f:Glomeraceae	g:Funneliformis	FOTU_541
7	709	d:Fungi	p:Ascomycota	c:Unidentified	o:Unidentified	f:Unidentified	g:Unidentified	FOTU_1015
8	691	d:Fungi	p:Glomeromycota	c:Paraglomeromycetes	o:Paraglomerales	f:Paraglomeraceae	g:Paraglomus	FOTU_1134
8	596	d:Fungi	p:Glomeromycota	c:Archaeosporomycetes	o:Archaeosporales	f:Ambisporaceae	g:Ambispora	FOTU_1854

d=degree;b/c=betweeness/centrality

(continued). All nodes determined in the Co-ocurrence network analyses.

domain	phylum	class	order	family	genus	otu.id
k:Bacteria	p:Actinobacteria	c:Acidimicrobiia	o:Acidimicrobiales	f:Unidentified	g:Unidentified	POTU_379
k:Bacteria	p:Actinobacteria	c:Actinobacteria	o:Actinomycetales	f:Actinomycetaceae	g:Actinomyces	POTU_565
k:Bacteria	p:Proteobacteria	c:Gammaproteobacteria	o:Aeromonadales	f:Aeromonadaceae	g:Aeromonas	POTU_88
d:Fungi	p:Glomeromycota	c:Archaeosporomycetes	o:Archaeosporales	f:Ambisporaceae	g:Ambispora	FOTU_87
d:Fungi	p:Glomeromycota	c:Archaeosporomycetes	o:Archaeosporales	f:Ambisporaceae	g:Ambispora	FOTU_140
d:Fungi	p:Glomeromycota	c:Archaeosporomycetes	o:Archaeosporales	f:Ambisporaceae	g:Ambispora	FOTU_195
d:Fungi	p:Glomeromycota	c:Archaeosporomycetes	o:Archaeosporales	f:Ambisporaceae	g:Ambispora	FOTU_256
d:Fungi	p:Glomeromycota	c:Archaeosporomycetes	o:Archaeosporales	f:Ambisporaceae	g:Ambispora	FOTU_490
d:Fungi	p:Glomeromycota	c:Archaeosporomycetes	o:Archaeosporales	f:Ambisporaceae	g:Ambispora	FOTU_813
d:Fungi	p:Glomeromycota	c:Archaeosporomycetes	o:Archaeosporales	f:Ambisporaceae	g:Ambispora	FOTU_1854
d:Fungi	p:Glomeromycota	c:Archaeosporomycetes	o:Archaeosporales	f:Ambisporaceae	g:Ambispora	FOTU_3092
k:Bacteria	p:Gemmatimonade	c:Gemmatimonadetes	o:AT425-EubC11	f:Unidentified	g:Unidentified	POTU_169
k:Bacteria	p:Tenericutes	c:Bacilli	o:Bacillales	f:Bacillaceae	g:Bacillus	POTU_14
k:Bacteria	p:Tenericutes	c:Bacilli	o:Bacillales	f:Bacillaceae	g:Bacillus	POTU_31
k:Bacteria	p:Tenericutes	c:Bacilli	o:Bacillales	f:Bacillaceae	g:Bacillus	POTU_34
k:Bacteria	p:Tenericutes	c:Bacilli	o:Bacillales	f:Bacillaceae	g:Bacillus	POTU_80
k:Bacteria	p:Tenericutes	c:Bacilli	o:Bacillales	f:Staphylococcaceae	g:Staphylococcus	POTU_87
k:Bacteria	p:Tenericutes	c:Bacilli	o:Bacillales	f:Bacillaceae	g:Bacillus	POTU_163
k:Bacteria	p:Tenericutes	c:Bacilli	o:Bacillales	f:Family_XI	g:Gemella	POTU_298
k:Bacteria	p:Tenericutes	c:Bacilli	o:Bacillales	f:Alicyclobacillaceae	g:Tumebacillus	POTU_311
k:Bacteria	p:Tenericutes	c:Bacilli	o:Bacillales	f:Bacillaceae	g:Bacillus	POTU_347
k:Bacteria	p:Tenericutes	c:Bacilli	o:Bacillales	f:Bacillaceae	g:Bacillus	POTU_401
k:Bacteria	p:Tenericutes	c:Bacilli	o:Bacillales	f:Planococcaceae	g:Lysinibacillus	POTU_527
k:Bacteria	p:Tenericutes	c:Bacilli	o:Bacillales	f:Bacillaceae	g:Unidentified	POTU_559
k:Bacteria	p:Tenericutes	c:Bacilli	o:Bacillales	f:Bacillaceae	g:Bacillus	POTU_579
k:Bacteria	p:Tenericutes	c:Bacilli	o:Bacillales	f:Family_XII	g:Exiguobacterium	POTU_657
k:Bacteria	p:Tenericutes	c:Bacilli	o:Bacillales	f:Bacillaceae	g:Bacillus	POTU_927
k:Bacteria	p:Tenericutes	c:Bacilli	o:Bacillales	f:Bacillaceae	g:Bacillus	POTU_929
k:Bacteria	p:Tenericutes	c:Bacilli	o:Bacillales	f:Planococcaceae	g:Chryseomicrobium	POTU_1383
k:Bacteria	p:Tenericutes	c:Bacilli	o:Bacillales	f:Planococcaceae	g:Rummeliibacillus	POTU_1663

k:Bacteria	p:Tenericutes	c:Bacilli	o:Bacillales	f:Paenibacillaceae	g:Paenibacillus	POTU_2221
k:Bacteria	p:Tenericutes	c:Bacilli	o:Bacillales	f:Bacillaceae	g:Bacillus	POTU_2304
k:Bacteria	p:Tenericutes	c:Bacilli	o:Bacillales	f:Bacillaceae	g:Unidentified	POTU_2764
k:Bacteria	p:Bacteroidetes	c:Bacteroidia	o:Bacteroidales	f:Porphyromonadacea	g:Porphyromonas	POTU_511
k:Bacteria	p:Bacteroidetes	c:Bacteroidia	o:Bacteroidales	f:Prevotellaceae	g:Prevotella	POTU_664
k:Bacteria	p:Bacteroidetes	c:Bacteroidia	o:Bacteroidales	f:Prevotellaceae	g:Alloprevotella	POTU_686
k:Bacteria	p:Bacteroidetes	c:Bacteroidia	o:Bacteroidales	f:Prevotellaceae	g:Alloprevotella	POTU_2220
d:Fungi	p:Ascomycota	c:Dothideomycetes	o:Botryosphaeriales	f:Botryosphaeriaceae	g:Macrophomina	FOTU_24
d:Fungi	p:Ascomycota	c:Sordariomycetes	o:Branch06	f:Unidentified	g:Unidentified	FOTU_394
k:Bacteria	p:Proteobacteria	c:Betaproteobacteria	o:Burkholderiales	f:Comamonadaceae	g:Unidentified	POTU_24
k:Bacteria	p:Proteobacteria	c:Betaproteobacteria	o:Burkholderiales	f:Oxalobacteraceae	g:Noviherbaspirillum	POTU_36
k:Bacteria	p:Proteobacteria	c:Betaproteobacteria	o:Burkholderiales	f:Comamonadaceae	g:Unidentified	POTU_116
k:Bacteria	p:Proteobacteria	c:Betaproteobacteria	o:Burkholderiales	f:Comamonadaceae	g:Unidentified	POTU_201
k:Bacteria	p:Proteobacteria	c:Betaproteobacteria	o:Burkholderiales	f:Comamonadaceae	g:Unidentified	POTU_461
k:Bacteria	p:Proteobacteria	c:Betaproteobacteria	o:Burkholderiales	f:Burkholderiaceae	g:Ralstonia	POTU_516
k:Bacteria	p:Proteobacteria	c:Betaproteobacteria	o:Burkholderiales	f:Comamonadaceae	g:Unidentified	POTU_544
k:Bacteria	p:Proteobacteria	c:Betaproteobacteria	o:Burkholderiales	f:Burkholderiaceae	g:Ca_Glomeribacter	POTU_568
k:Bacteria	p:Proteobacteria	c:Betaproteobacteria	o:Burkholderiales	f:Comamonadaceae	g:Unidentified	POTU_939
k:Bacteria	p:Proteobacteria	c:Betaproteobacteria	o:Burkholderiales	f:Comamonadaceae	g:Schlegelella	POTU_2313
k:Bacteria	p:Proteobacteria	c:Betaproteobacteria	o:Burkholderiales	f:Burkholderiaceae	g:Ca_Glomeribacter	POTU_2447
k:Bacteria	p:Proteobacteria	c:Betaproteobacteria	o:Burkholderiales	f:Comamonadaceae	g:Unidentified	POTU_3451
k:Bacteria	p:Proteobacteria	c:Betaproteobacteria	o:Burkholderiales	f:Comamonadaceae	g:Unidentified	POTU_6198
k:Bacteria	p:Proteobacteria	c:Betaproteobacteria	o:Burkholderiales	f:Comamonadaceae	g:Unidentified	POTU_6345
k:Bacteria	p:Proteobacteria	c:Betaproteobacteria	o:Burkholderiales	f:Comamonadaceae	g:Unidentified	POTU_7468
k:Bacteria	p:Proteobacteria	c:Epsilonproteobacteria	o:Campylobacterales	f:Campylobacteracea	g:Campylobacter	POTU_869
k:Bacteria	p:Proteobacteria	c:Alphaproteobacteria	o:Caulobacterales	f:Caulobacteraceae	g:Brevundimonas	POTU_90
k:Bacteria	p:Proteobacteria	c:Alphaproteobacteria	o:Caulobacterales	f:Caulobacteraceae	g:Brevundimonas	POTU_134
k:Bacteria	p:Proteobacteria	c:Gammaproteobacteria	o:Chromatiales	f:Chromatiaceae	g:Rheinheimera	POTU_981
k:Bacteria	p:Tenericutes	c:Clostridia	o:Clostridiales	f:Family_XI	g:Peptoniphilus	POTU_419
k:Bacteria	p:Tenericutes	c:Clostridia	o:Clostridiales	f:Family_XI	g:Finegoldia	POTU_628
k:Bacteria	p:Tenericutes	c:Clostridia	o:Clostridiales	f:Lachnospiraceae	g:Lachnoanaerobaculum	POTU_960
k:Bacteria	p:Tenericutes	c:Clostridia	o:Clostridiales	f:Family_XI	g:Anaerococcus	POTU_1528
k:Bacteria	p:Tenericutes	c:Clostridia	o:Clostridiales	f:Family_XI	g:Anaerococcus	POTU_2379

k:Bacteria	p:Actinobacteria	c:Actinobacteria	o:Corynebacteriales	f:Corynebacteriaceae	g:Corynebacterium_1	POTU_128
k:Bacteria	p:Actinobacteria	c:Actinobacteria	o:Corynebacteriales	f:Corynebacteriaceae	g:Corynebacterium_1	POTU_268
k:Bacteria	p:Actinobacteria	c:Actinobacteria	o:Corynebacteriales	f:Corynebacteriaceae	g:Unidentified	POTU_309
k:Bacteria	p:Actinobacteria	c:Actinobacteria	o:Corynebacteriales	f:Nocardiaceae	g:Rhodococcus	POTU_371
k:Bacteria	p:Actinobacteria	c:Actinobacteria	o:Corynebacteriales	f:Corynebacteriaceae	g:Corynebacterium	POTU_412
k:Bacteria	p:Actinobacteria	c:Actinobacteria	o:Corynebacteriales	f:Corynebacteriaceae	g:Corynebacterium	POTU_1194
d:Fungi	p:Basidiomycota	c:Tremellomycetes	o:Cystofilobasidiales	f:Mrakiaceae	g:Tausonia	FOTU_1789
k:Bacteria	p:Bacteroidetes	c:Cytophagia	o:Cytophagales	f:Cytophagaceae	g:Ohtaekwangia	POTU_64
k:Bacteria	p:Bacteroidetes	c:Cytophagia	o:Cytophagales	f:Cytophagaceae	g:Rhodocytophaga	POTU_518
k:Bacteria	p:Bacteroidetes	c:Cytophagia	o:Cytophagales	f:Cytophagaceae	g:Unidentified	POTU_618
k:Bacteria	p:Bacteroidetes	c:Cytophagia	o:Cytophagales	f:Cytophagaceae	g:Unidentified	POTU_1009
d:Fungi	p:Glomeromycota	c:Glomeromycetes	o:Diversisporales	f:Acaulosporaceae	g:Acaulospora	FOTU_6070
k:Bacteria	p:Proteobacteria	c:Gammaproteobacteria	o:Enterobacteriales	f:Enterobacteriaceae	g:Pantoea	POTU_3
k:Bacteria	p:Proteobacteria	c:Gammaproteobacteria	o:Enterobacteriales	f:Enterobacteriaceae	g:Unidentified	POTU_91
d:Fungi	p:Ascomycota	c:Eurotiomycetes	o:Eurotiales	f:Aspergillaceae	g:Penicillium	FOTU_16
k:Bacteria	p:Bacteroidetes	c:Flavobacteriia	o:Flavobacteriales	f:Flavobacteriaceae	g:Flavobacterium	POTU_38
k:Bacteria	p:Bacteroidetes	c:Flavobacteriia	o:Flavobacteriales	f:Flavobacteriaceae	g:Flavobacterium	POTU_609
k:Bacteria	p:Actinobacteria	c:Actinobacteria	o:Frankiales	f:Nakamurellaceae	g:Nakamurella	POTU_2026
k:Bacteria	p:Fusobacteria	c:Fusobacteriia	o:Fusobacteriales	f:Leptotrichiaceae	g:Leptotrichia	POTU_177
k:Bacteria	p:Fusobacteria	c:Fusobacteriia	o:Fusobacteriales	f:Fusobacteriaceae	g:Fusobacterium	POTU_410
k:Bacteria	p:Fusobacteria	c:Fusobacteriia	o:Fusobacteriales	f:Leptotrichiaceae	g:Leptotrichia	POTU_467
k:Bacteria	p:Fusobacteria	c:Fusobacteriia	o:Fusobacteriales	f:Leptotrichiaceae	g:Leptotrichia	POTU_3373
d:Fungi	p:Glomeromycota	c:Glomeromycetes	o:Glomerales	f:Glomeraceae	g:Funneliformis	FOTU_541
d:Fungi	p:Glomeromycota	c:Glomeromycetes	o:Glomerales	f:Glomeraceae	g:Glomus	FOTU_4854
d:Fungi	p:Glomeromycota	c:Glomeromycetes	o:Glomerales	f:Glomeraceae	g:Glomus	FOTU_5940
d:Fungi	p:Glomeromycota	c:Glomeromycetes	o:Glomerales	f:Glomeraceae	g:Rhizophagus	FOTU_6029
d:Fungi	p:Glomeromycota	c:Glomeromycetes	o:Glomerales	f:Glomeraceae	g:Glomus	FOTU_7004
d:Fungi	p:Glomeromycota	c:Glomeromycetes	o:Glomerales	f:Glomeraceae	g:Glomus	FOTU_8586
d:Fungi	p:Glomeromycota	c:Glomeromycetes	o:Glomerales	f:Glomeraceae	g:Rhizophagus	FOTU_8803
d:Fungi	p:Ascomycota	c:Leotiomycetes	o:Helotiales	f:Helotiales_fam_Incei	g:Leohumicola	FOTU_533
d:Fungi	p:Ascomycota	c:Sordariomycetes	o:Hypocreales	f:Nectriaceae	g:Fusarium	FOTU_6
d:Fungi	p:Ascomycota	c:Sordariomycetes	o:Hypocreales	f:Nectriaceae	g:Unidentified	FOTU_12
d:Fungi	p:Ascomycota	c:Sordariomycetes	o:Hypocreales	f:Cordycipitaceae	g:Beauveria	FOTU_3644

k:Bacteria	p:Tenericutes	c:Bacilli	o:Lactobacillales	f:Streptococcaceae	g:Streptococcus	POTU_215
k:Bacteria	p:Tenericutes	c:Bacilli	o:Lactobacillales	f:Carnobacteriaceae	g:Granulicatella	POTU_328
k:Bacteria	p:Tenericutes	c:Bacilli	o:Lactobacillales	f:Unidentified	g:Unidentified	POTU_636
k:Bacteria	p:Tenericutes	c:Bacilli	o:Lactobacillales	f:Streptococcaceae	g:Streptococcus	POTU_3897
k:Bacteria	p:Tenericutes	c:Bacilli	o:Lactobacillales	f:Unidentified	g:Unidentified	POTU_21098
k:Bacteria	p:Actinobacteria	c:Actinobacteria	o:Micrococcales	f:Cellulomonadaceae	g:Unidentified	POTU_68
k:Bacteria	p:Actinobacteria	c:Actinobacteria	o:Micrococcales	f:Micrococcaceae	g:Rothia	POTU_564
k:Bacteria	p:Actinobacteria	c:Actinobacteria	o:Micrococcales	f:Micrococcaceae	g:Rothia	POTU_1000
k:Bacteria	p:Actinobacteria	c:Actinobacteria	o:Micrococcales	f:Microbacteriaceae	g:Unidentified	POTU_1157
k:Bacteria	p:Actinobacteria	c:Actinobacteria	o:Micromonosporales	f:Micromonosporacea	g:Unidentified	POTU_5
k:Bacteria	p:Actinobacteria	c:Actinobacteria	o:Micromonosporales	f:Micromonosporacea	g:Unidentified	POTU_66
k:Bacteria	p:Actinobacteria	c:Actinobacteria	o:Micromonosporales	f:Micromonosporacea	g:Unidentified	POTU_199
k:Bacteria	p:Actinobacteria	c:Actinobacteria	o:Micromonosporales	f:Micromonosporacea	g:Actinoplanes	POTU_247
k:Bacteria	p:Actinobacteria	c:Actinobacteria	o:Micromonosporales	f:Micromonosporacea	g:Unidentified	POTU_312
k:Bacteria	p:Actinobacteria	c:Actinobacteria	o:Micromonosporales	f:Micromonosporacea	g:Actinoplanes	POTU_388
k:Bacteria	p:Actinobacteria	c:Actinobacteria	o:Micromonosporales	f:Micromonosporacea	g:Unidentified	POTU_1277
d:Fungi	p:Mucoromycota	c:Mucoromycetes	o:Mucorales	f:Rhizopodaceae	g:Rhizopus	FOTU_96
k:Bacteria	p:Tenericutes	c:Mollicutes	o:Mycoplasmatales	f:Mycoplasmataceae	g:Ca_Moeniiplasma	POTU_27
k:Bacteria	p:Tenericutes	c:Mollicutes	o:Mycoplasmatales	f:Mycoplasmataceae	g:Ca_Moeniiplasma	POTU_42
k:Bacteria	p:Tenericutes	c:Mollicutes	o:Mycoplasmatales	f:Mycoplasmataceae	g:Ca_Moeniiplasma	POTU_52
k:Bacteria	p:Tenericutes	c:Mollicutes	o:Mycoplasmatales	f:Mycoplasmataceae	g:Ca_Moeniiplasma	POTU_57
k:Bacteria	p:Tenericutes	c:Mollicutes	o:Mycoplasmatales	f:Mycoplasmataceae	g:Ca_Moeniiplasma	POTU_108
k:Bacteria	p:Tenericutes	c:Mollicutes	o:Mycoplasmatales	f:Mycoplasmataceae	g:Ca_Moeniiplasma	POTU_109
k:Bacteria	p:Tenericutes	c:Mollicutes	o:Mycoplasmatales	f:Mycoplasmataceae	g:Ca_Moeniiplasma	POTU_112
k:Bacteria	p:Tenericutes	c:Mollicutes	o:Mycoplasmatales	f:Mycoplasmataceae	g:Ca_Moeniiplasma	POTU_119
k:Bacteria	p:Tenericutes	c:Mollicutes	o:Mycoplasmatales	f:Mycoplasmataceae	g:Ca_Moeniiplasma	POTU_151
k:Bacteria	p:Tenericutes	c:Mollicutes	o:Mycoplasmatales	f:Mycoplasmataceae	g:Ca_Moeniiplasma	POTU_244
k:Bacteria	p:Tenericutes	c:Mollicutes	o:Mycoplasmatales	f:Mycoplasmataceae	g:Ca_Moeniiplasma	POTU_295
k:Bacteria	p:Tenericutes	c:Mollicutes	o:Mycoplasmatales	f:Mycoplasmataceae	g:Ca_Moeniiplasma	POTU_354
k:Bacteria	p:Tenericutes	c:Mollicutes	o:Mycoplasmatales	f:Mycoplasmataceae	g:Ca_Moeniiplasma	POTU_526
k:Bacteria	p:Tenericutes	c:Mollicutes	o:Mycoplasmatales	f:Mycoplasmataceae	g:Ca_Moeniiplasma	POTU_533
k:Bacteria	p:Tenericutes	c:Mollicutes	o:Mycoplasmatales	f:Mycoplasmataceae	g:Ca_Moeniiplasma	POTU_578
k:Bacteria	p:Tenericutes	c:Mollicutes	o:Mycoplasmatales	f:Mycoplasmataceae	g:Ca_Moeniiplasma	POTU_672

k:Bacteria	p:Tenericutes	c:Mollicutes	o:Mycoplasmatales	f:Mycoplasmataceae	g:Ca_Moeniiplasma	POTU_685
k:Bacteria	p:Tenericutes	c:Mollicutes	o:Mycoplasmatales	f:Mycoplasmataceae	g:Ca_Moeniiplasma	POTU_691
k:Bacteria	p:Tenericutes	c:Mollicutes	o:Mycoplasmatales	f:Mycoplasmataceae	g:Ca_Moeniiplasma	POTU_876
k:Bacteria	p:Tenericutes	c:Mollicutes	o:Mycoplasmatales	f:Mycoplasmataceae	g:Ca_Moeniiplasma	POTU_948
k:Bacteria	p:Tenericutes	c:Mollicutes	o:Mycoplasmatales	f:Mycoplasmataceae	g:Ca_Moeniiplasma	POTU_989
k:Bacteria	p:Tenericutes	c:Mollicutes	o:Mycoplasmatales	f:Mycoplasmataceae	g:Ca_Moeniiplasma	POTU_1023
k:Bacteria	p:Tenericutes	c:Mollicutes	o:Mycoplasmatales	f:Mycoplasmataceae	g:Ca_Moeniiplasma	POTU_1199
k:Bacteria	p:Tenericutes	c:Mollicutes	o:Mycoplasmatales	f:Mycoplasmataceae	g:Ca_Moeniiplasma	POTU_1262
k:Bacteria	p:Tenericutes	c:Mollicutes	o:Mycoplasmatales	f:Mycoplasmataceae	g:Ca_Moeniiplasma	POTU_1263
k:Bacteria	p:Tenericutes	c:Mollicutes	o:Mycoplasmatales	f:Mycoplasmataceae	g:Ca_Moeniiplasma	POTU_1536
k:Bacteria	p:Tenericutes	c:Mollicutes	o:Mycoplasmatales	f:Mycoplasmataceae	g:Ca_Moeniiplasma	POTU_1631
k:Bacteria	p:Tenericutes	c:Mollicutes	o:Mycoplasmatales	f:Mycoplasmataceae	g:Ca_Moeniiplasma	POTU_2083
k:Bacteria	p:Tenericutes	c:Mollicutes	o:Mycoplasmatales	f:Mycoplasmataceae	g:Ca_Moeniiplasma	POTU_2126
k:Bacteria	p:Tenericutes	c:Mollicutes	o:Mycoplasmatales	f:Mycoplasmataceae	g:Ca_Moeniiplasma	POTU_2276
k:Bacteria	p:Tenericutes	c:Mollicutes	o:Mycoplasmatales	f:Mycoplasmataceae	g:Ca_Moeniiplasma	POTU_2322
k:Bacteria	p:Tenericutes	c:Mollicutes	o:Mycoplasmatales	f:Mycoplasmataceae	g:Ca_Moeniiplasma	POTU_2489
k:Bacteria	p:Tenericutes	c:Mollicutes	o:Mycoplasmatales	f:Mycoplasmataceae	g:Ca_Moeniiplasma	POTU_2715
k:Bacteria	p:Tenericutes	c:Mollicutes	o:Mycoplasmatales	f:Mycoplasmataceae	g:Ca_Moeniiplasma	POTU_3014
k:Bacteria	p:Tenericutes	c:Mollicutes	o:Mycoplasmatales	f:Mycoplasmataceae	g:Ca_Moeniiplasma	POTU_3159
k:Bacteria	p:Tenericutes	c:Mollicutes	o:Mycoplasmatales	f:Mycoplasmataceae	g:Ca_Moeniiplasma	POTU_3239
k:Bacteria	p:Tenericutes	c:Mollicutes	o:Mycoplasmatales	f:Mycoplasmataceae	g:Ca_Moeniiplasma	POTU_4062
k:Bacteria	p:Tenericutes	c:Mollicutes	o:Mycoplasmatales	f:Mycoplasmataceae	g:Ca_Moeniiplasma	POTU_4065
k:Bacteria	p:Tenericutes	c:Mollicutes	o:Mycoplasmatales	f:Mycoplasmataceae	g:Ca_Moeniiplasma	POTU_5576
k:Bacteria	p:Tenericutes	c:Mollicutes	o:Mycoplasmatales	f:Mycoplasmataceae	g:Ca_Moeniiplasma	POTU_8537
k:Bacteria	p:Tenericutes	c:Mollicutes	o:Mycoplasmatales	f:Mycoplasmataceae	g:Ca_Moeniiplasma	POTU_9242
k:Bacteria	p:Tenericutes	c:Mollicutes	o:Mycoplasmatales	f:Mycoplasmataceae	g:Ca_Moeniiplasma	POTU_14920
k:Bacteria	p:Tenericutes	c:Mollicutes	o:Mycoplasmatales	f:Mycoplasmataceae	g:Ca_Moeniiplasma	POTU_25136
k:Bacteria	p:Proteobacteria	c:Deltaproteobacteria	o:Myxococcales	f:Sandaracinaceae	g:Unidentified	POTU_228
k:Bacteria	p:Proteobacteria	c:Deltaproteobacteria	o:Myxococcales	f:Haliangiaceae	g:Haliangium	POTU_373
k:Bacteria	p:Proteobacteria	c:Betaproteobacteria	o:Neisseriales	f:Neisseriaceae	g:Neisseria	POTU_195
k:Bacteria	p:Proteobacteria	c:Betaproteobacteria	o:Neisseriales	f:Neisseriaceae	g:Neisseria	POTU_416
k:Bacteria	p:Proteobacteria	c:Betaproteobacteria	o:Neisseriales	f:Neisseriaceae	g:Kingella	POTU_765
k:Bacteria	p:Proteobacteria	c:Betaproteobacteria	o:Neisseriales	f:Neisseriaceae	g:Vogesella	POTU_1241

k:Bacteria	p:Proteobacteria	c:Betaproteobacteria	o:Neisseriales	f:Neisseriaceae	g:Unidentified	POTU_1524
k:Bacteria	p:Proteobacteria	c:Betaproteobacteria	o:Neisseriales	f:Neisseriaceae	g:Unidentified	POTU_2941
k:Bacteria	p:Proteobacteria	c:Deltaproteobacteria	o:Oligoflexales	f:Unidentified	g:Unidentified	POTU_442
d:Fungi	p:Ascomycota	c:Eurotiomycetes	o:Onygenales	f:Onygenaceae	g:Uncinocarpus	FOTU_85
d:Fungi	p:Ascomycota	c:Eurotiomycetes	o:Onygenales	f:Unidentified	g:Unidentified	FOTU_185
d:Fungi	p:Glomeromycota	c:Paraglomeromycetes	o:Paraglomerales	f:Paraglomeraceae	g:Paraglomus	FOTU_1134
d:Fungi	p:Glomeromycota	c:Paraglomeromycetes	o:Paraglomerales	f:Paraglomeraceae	g:Paraglomus	FOTU_1278
k:Bacteria	p:Proteobacteria	c:Gammaproteobacteria	o:Pasteurellales	f:Pasteurellaceae	g:Haemophilus	POTU_139
k:Bacteria	p:Proteobacteria	c:Gammaproteobacteria	o:Pasteurellales	f:Pasteurellaceae	g:Unidentified	POTU_429
k:Bacteria	p:Proteobacteria	c:Gammaproteobacteria	o:Pasteurellales	f:Pasteurellaceae	g:Aggregatibacter	POTU_514
k:Bacteria	p:Proteobacteria	c:Gammaproteobacteria	o:Pasteurellales	f:Pasteurellaceae	g:Haemophilus	POTU_10174
d:Fungi	p:Ascomycota	c:Pezizomycetes	o:Pezizales	f:Pyronemataceae	g:Scutellinia	FOTU_481
k:Bacteria	p:Actinobacteria	c:Actinobacteria	o:Propionibacteriales	f:Propionibacteriaceae	g:Propionibacterium	POTU_130
k:Bacteria	p:Actinobacteria	c:Actinobacteria	o:Propionibacteriales	f:Nocardioidaceae	g:Kribbella	POTU_235
k:Bacteria	p:Actinobacteria	c:Actinobacteria	o:Propionibacteriales	f:Propionibacteriaceae	g:Propionibacterium	POTU_2994
k:Bacteria	p:Proteobacteria	c:Gammaproteobacteria	o:Pseudomonadales	f:Pseudomonadaceae	g:Pseudomonas	POTU_11
k:Bacteria	p:Proteobacteria	c:Gammaproteobacteria	o:Pseudomonadales	f:Pseudomonadaceae	g:Pseudomonas	POTU_30
k:Bacteria	p:Proteobacteria	c:Gammaproteobacteria	o:Pseudomonadales	f:Moraxellaceae	g:Acinetobacter	POTU_93
k:Bacteria	p:Proteobacteria	c:Gammaproteobacteria	o:Pseudomonadales	f:Moraxellaceae	g:Moraxella	POTU_175
k:Bacteria	p:Proteobacteria	c:Gammaproteobacteria	o:Pseudomonadales	f:Pseudomonadaceae	g:Pseudomonas	POTU_202
k:Bacteria	p:Proteobacteria	c:Gammaproteobacteria	o:Pseudomonadales	f:Pseudomonadaceae	g:Pseudomonas	POTU_581
k:Bacteria	p:Proteobacteria	c:Gammaproteobacteria	o:Pseudomonadales	f:Pseudomonadaceae	g:Pseudomonas	POTU_806
k:Bacteria	p:Proteobacteria	c:Gammaproteobacteria	o:Pseudomonadales	f:Moraxellaceae	g:Acinetobacter	POTU_1072
k:Bacteria	p:Proteobacteria	c:Gammaproteobacteria	o:Pseudomonadales	f:Pseudomonadaceae	g:Pseudomonas	POTU_1112
k:Bacteria	p:Proteobacteria	c:Gammaproteobacteria	o:Pseudomonadales	f:Pseudomonadaceae	g:Pseudomonas	POTU_3157
k:Bacteria	p:Proteobacteria	c:Gammaproteobacteria	o:Pseudomonadales	f:Pseudomonadaceae	g:Pseudomonas	POTU_3717
k:Bacteria	p:Proteobacteria	c:Gammaproteobacteria	o:Pseudomonadales	f:Pseudomonadaceae	g:Pseudomonas	POTU_7227
k:Bacteria	p:Proteobacteria	c:Gammaproteobacteria	o:Pseudomonadales	f:Pseudomonadaceae	g:Pseudomonas	POTU_25191
k:Bacteria	p:Actinobacteria	c:Actinobacteria	o:Pseudonocardiales	f:Pseudonocardiacea	g:Lentzea	POTU_94
k:Bacteria	p:Actinobacteria	c:Actinobacteria	o:Pseudonocardiales	f:Pseudonocardiacea	g:Amycolatopsis	POTU_100
k:Bacteria	p:Actinobacteria	c:Actinobacteria	o:Pseudonocardiales	f:Pseudonocardiacea	g:Pseudonocardia	POTU_101
k:Bacteria	p:Actinobacteria	c:Actinobacteria	o:Pseudonocardiales	f:Pseudonocardiacea	g:Unidentified	POTU_105
k:Bacteria	p:Actinobacteria	c:Actinobacteria	o:Pseudonocardiales	f:Pseudonocardiacea	g:Unidentified	POTU_1119

k:Bacteria	p:Actinobacteria	c:Actinobacteria	o:Pseudonocardiales	f:Pseudonocardiacea	g:Actinophytocola	POTU_1225
k:Bacteria	p:Actinobacteria	c:Actinobacteria	o:Pseudonocardiales	f:Pseudonocardiacea	g:Unidentified	POTU_1779
k:Bacteria	p:Actinobacteria	c:Actinobacteria	o:Pseudonocardiales	f:Pseudonocardiacea	g:Actinoalloteichus	POTU_2454
k:Bacteria	p:Actinobacteria	c:Actinobacteria	o:Pseudonocardiales	f:Pseudonocardiacea	g:Unidentified	POTU_2714
k:Bacteria	p:Actinobacteria	c:Actinobacteria	o:Pseudonocardiales	f:Pseudonocardiacea	g:Amycolatopsis	POTU_10983
k:Bacteria	p:Proteobacteria	c:Alphaproteobacteria	o:Rhizobiales	f:Rhizobiaceae	g:Rhizobium	POTU_19
k:Bacteria	p:Proteobacteria	c:Alphaproteobacteria	o:Rhizobiales	f:Bradyrhizobiaceae	g:Bradyrhizobium	POTU_45
k:Bacteria	p:Proteobacteria	c:Alphaproteobacteria	o:Rhizobiales	f:Rhizobiaceae	g:Rhizobium	POTU_58
k:Bacteria	p:Proteobacteria	c:Alphaproteobacteria	o:Rhizobiales	f:Hyphomicrobiaceae	g:Devosia	POTU_75
k:Bacteria	p:Proteobacteria	c:Alphaproteobacteria	o:Rhizobiales	f:Bradyrhizobiaceae	g:Bosea	POTU_200
k:Bacteria	p:Proteobacteria	c:Alphaproteobacteria	o:Rhizobiales	f:Phyllobacteriaceae	g:Nitratireductor	POTU_296
k:Bacteria	p:Proteobacteria	c:Alphaproteobacteria	o:Rhizobiales	f:Phyllobacteriaceae	g:Phyllobacterium	POTU_468
k:Bacteria	p:Proteobacteria	c:Alphaproteobacteria	o:Rhizobiales	f:Hyphomicrobiaceae	g:Pedomicrobium	POTU_482
k:Bacteria	p:Proteobacteria	c:Alphaproteobacteria	o:Rhizobiales	f:Rhizobiaceae	g:Shinella	POTU_501
k:Bacteria	p:Proteobacteria	c:Alphaproteobacteria	o:Rhizobiales	f:Rhizobiaceae	g:Rhizobium	POTU_1270
k:Bacteria	p:Proteobacteria	c:Alphaproteobacteria	o:Rhodobacterales	f:Rhodobacteraceae	g:Paracocccus	POTU_70
k:Bacteria	p:Proteobacteria	c:Alphaproteobacteria	o:Rhodobacterales	f:Rhodobacteraceae	g:Paracocccus	POTU_123
k:Bacteria	p:Proteobacteria	c:Alphaproteobacteria	o:Rhodobacterales	f:Rhodobacteraceae	g:Rubellimicrobium	POTU_403
k:Bacteria	p:Proteobacteria	c:Betaproteobacteria	o:Rhodocyclales	f:Rhodocyclaceae	g:Unidentified	POTU_1215
d:Fungi	p:Ascomycota	c:Saccharomycetes	o:Saccharomycetales	f:Saccharomycetales_	g:Candida	FOTU_2730
d:Fungi	p:Ascomycota	c:Saccharomycetes	o:Saccharomycetales	f:Unidentified	g:Unidentified	FOTU_5404
d:Fungi	p:Ascomycota	c:Saccharomycetes	o:Saccharomycetales	f:Saccharomycetales_	g:Candida	FOTU_8943
k:Bacteria	p:Tenericutes	c:Negativicutes	o:Selenomonadales	f:Veillonellaceae	g:Veillonella	POTU_668
k:Bacteria	p:Tenericutes	c:Negativicutes	o:Selenomonadales	f:Veillonellaceae	g:Veillonella	POTU_1456
d:Fungi	p:Ascomycota	c:Sordariomycetes	o:Sordariales	f:Chaetomiaceae	g:Unidentified	FOTU_41
d:Fungi	p:Ascomycota	c:Sordariomycetes	o:Sordariales	f:Unidentified	g:Unidentified	FOTU_643
d:Fungi	p:Ascomycota	c:Sordariomycetes	o:Sordariales	f:Unidentified	g:Unidentified	FOTU_1324
k:Bacteria	p:Proteobacteria	c:Alphaproteobacteria	o:Sphingomonadales	f:Sphingomonadacea	g:Sphingobium	POTU_757
d:Fungi	p:Basidiomycota	c:Microbotryomycetes	o:Sporidiobolales	f:Sporidiobolaceae	g:Rhodotorula	FOTU_768
k:Bacteria	p:Actinobacteria	c:Actinobacteria	o:Streptomycetales	f:Streptomycetaceae	g:Unidentified	POTU_32
k:Bacteria	p:Actinobacteria	c:Actinobacteria	o:Streptomycetales	f:Streptomycetaceae	g:Streptomyces	POTU_35
k:Bacteria	p:Actinobacteria	c:Actinobacteria	o:Streptomycetales	f:Streptomycetaceae	g:Unidentified	POTU_3223
k:Bacteria	p:Actinobacteria	c:Actinobacteria	o:Streptomycetales	f:Streptomycetaceae	g:Streptomyces	POTU_7668

k:Bacteria	p:Actinobacteria	c:Actinobacteria	o:Streptosporangiales	f:Streptosporangiacea	g:Nonomuraea	POTU_353
k:Bacteria	p:Actinobacteria	c:Actinobacteria	o:Streptosporangiales	f:Thermomonosporac	g:Actinomadura	POTU_611
k:Bacteria	p:Actinobacteria	c:Actinobacteria	o:Streptosporangiales	f:Thermomonosporac	g:Actinoallomurus	POTU_32259
k:Bacteria	p:Proteobacteria	c:Alphaproteobacteria	o:Unidentified	f:Unidentified	g:Unidentified	POTU_231
k:Bacteria	p:Proteobacteria	c:Unidentified	o:Unidentified	f:Unidentified	g:Unidentified	POTU_267
d:Unidentifi	p:Unidentified	c:Unidentified	o:Unidentified	f:Unidentified	g:Unidentified	POTU_705
k:Bacteria	p:Unidentified	c:Unidentified	o:Unidentified	f:Unidentified	g:Unidentified	POTU_992
k:Bacteria	p:Actinobacteria	c:Actinobacteria	o:Unidentified	f:Unidentified	g:Unidentified	POTU_1103
k:Bacteria	p:Proteobacteria	c:Unidentified	o:Unidentified	f:Unidentified	g:Unidentified	POTU_1311
k:Bacteria	p:Actinobacteria	c:Actinobacteria	o:Unidentified	f:Unidentified	g:Unidentified	POTU_1409
d:Fungi	p:Ascomycota	c:Dothideomycetes	o:Unidentified	f:Unidentified	g:Unidentified	FOTU_32
d:Unidentifi	p:Unidentified	c:Unidentified	o:Unidentified	f:Unidentified	g:Unidentified	FOTU_37
d:Unidentifi	p:Unidentified	c:Unidentified	o:Unidentified	f:Unidentified	g:Unidentified	FOTU_70
d:Unidentifi	p:Unidentified	c:Unidentified	o:Unidentified	f:Unidentified	g:Unidentified	FOTU_116
d:Unidentifi	p:Unidentified	c:Unidentified	o:Unidentified	f:Unidentified	g:Unidentified	FOTU_139
d:Unidentifi	p:Unidentified	c:Unidentified	o:Unidentified	f:Unidentified	g:Unidentified	FOTU_156
d:Unidentifi	p:Unidentified	c:Unidentified	o:Unidentified	f:Unidentified	g:Unidentified	FOTU_165
d:Unidentifi	p:Unidentified	c:Unidentified	o:Unidentified	f:Unidentified	g:Unidentified	FOTU_220
d:Unidentifi	p:Unidentified	c:Unidentified	o:Unidentified	f:Unidentified	g:Unidentified	FOTU_245
d:Fungi	p:Unidentified	c:Unidentified	o:Unidentified	f:Unidentified	g:Unidentified	FOTU_261
d:Fungi	p:Ascomycota	c:Dothideomycetes	o:Unidentified	f:Unidentified	g:Unidentified	FOTU_299
d:Fungi	p:Ascomycota	c:Unidentified	o:Unidentified	f:Unidentified	g:Unidentified	FOTU_649
d:Unidentifi	p:Unidentified	c:Unidentified	o:Unidentified	f:Unidentified	g:Unidentified	FOTU_877
d:Fungi	p:Ascomycota	c:Unidentified	o:Unidentified	f:Unidentified	g:Unidentified	FOTU_1015
d:Unidentifi	p:Unidentified	c:Unidentified	o:Unidentified	f:Unidentified	g:Unidentified	FOTU_1388
d:Unidentifi	p:Unidentified	c:Unidentified	o:Unidentified	f:Unidentified	g:Unidentified	FOTU_2239
d:Unidentifi	p:Unidentified	c:Unidentified	o:Unidentified	f:Unidentified	g:Unidentified	FOTU_7123
k:Bacteria	p:Proteobacteria	c:Gammaproteobacteria	o:Xanthomonadales	f:Xanthomonadaceae	g:Stenotrophomonas	POTU_308
k:Bacteria	p:Proteobacteria	c:Gammaproteobacteria	o:Xanthomonadales	f:Xanthomonadaceae	g:Lysobacter	POTU_431
k:Bacteria	p:Proteobacteria	c:Gammaproteobacteria	o:Xanthomonadales	f:Xanthomonadaceae	g:Stenotrophomonas	POTU_571
k:Bacteria	p:Proteobacteria	c:Gammaproteobacteria	o:Xanthomonadales	f:Xanthomonadaceae	g:Thermomonas	POTU_616

REFERENCES

Bianciotto, V., Bandi, C., Minerdi, D., Sironi, M., Tichy, H. V. and Bonfante, P. (1996) 'An obligately endosymbiotic mycorrhizal fungus itself harbors obligately intracellular bacteria', 62(8), pp. 3005-3010. Available at: https://aem.asm.org/content/aem/62/8/3005.full.pdf.

Bianciotto, V., Lumini, E., Lanfranco, L., Minerdi, D., Bonfante, P. and Perotto, S. (2000) 'Detection and identification of bacterial endosymbionts in arbuscular mycorrhizal fungi belonging to the family Gigasporaceae', *Appl Environ Microbiol,* 66(10), pp. 4503-9. DOI: 10.1128/aem.66.10.4503-4509.2000.

Blanchard, A., Yanez, A., Dybvig, K., Watson, H. L., Griffiths, G. and Cassell, G. H. (1993) 'Evaluation of intraspecies genetic variation within the 16S rRNA gene of Mycoplasma hominis and detection by polymerase chain reaction', *J Clin Microbiol,* 31(5), pp. 1358-61. DOI: 10.1128/jcm.31.5.1358-1361.1993.

Błaszkowski, J., Chwat, G. and Góralska, A. (2016) 'Dominikia lithuanica and Kamienskia divaricata: new species in the Glomeromycota', *Botany,* 94(12), pp. 1075-1085. DOI: 10.1139/cjb-2016-0167.

Blaszkowski, J., Niezgoda, P., Piatek, M., Magurno, F., Malicka, M., Zubek, S., Mleczko, P., Yorou, N. S., Jobim, K., Vista, X. M., Lima, J. L. R. and Goto, B. T. (2019) 'Rhizoglomus dalpeae, R. maiae, and R. silesianum, new species', *Mycologia,* 111(6), pp. 965-980. DOI: 10.1080/00275514.2019.1654637.

Blaszkowski, J., Sanchez-Garcia, M., Niezgoda, P., Zubek, S., Fernandez, F., Vila, A., Al-Yahya'ei, M. N., Symanczik, S., Milczarski, P., Malinowski, R., Cabello, M., Goto, B. T., Casieri, L., Malicka, M., Bierza, W. and Magurno, F. (2022) 'A new order, Entrophosporales, and three new Entrophospora species in Glomeromycota', *Front Microbiol,* 13, pp. 962856. DOI: 10.3389/fmicb.2022.962856.

Brett, P. J., DeShazer, D. and Woods, D. E. (1998) 'Burkholderia thailandensis sp. nov., a Burkholderia pseudomallei-like species', *Int J Syst Bacteriol,* 48 Pt 1, pp. 317-20. DOI: 10.1099/00207713-48-1-317.

Buttner, H., Pidot, S. J., Scherlach, K. and Hertweck, C. (2022) 'Endofungal bacteria boost anthelminthic host protection with the biosurfactant symbiosin', *Chem Sci,* 14(1), pp. 103-112. DOI: 10.1039/d2sc04167g.

Cabrera-Rangel, J. F., Mendoza-Servín, J. V., Córdova-López, G., Alcalde-Vázquez, R., García-Estrada, R. S., Winkler, R. and Partida-Martínez, L. P. (2022) 'Symbiotic and toxinogenic Rhizopus spp. isolated from soils of different papaya producing regions in Mexico', *Frontiers in Fungal Biology,* 3. DOI: 10.3389/ffunb.2022.893700.

Coenye, T., Laevens, S., Willems, A., Ohlen, M., Hannant, W., Govan, J. R., Gillis, M., Falsen, E. and Vandamme, P. (2001) 'Burkholderia fungorum sp. nov. and Burkholderia caledonica sp. nov., two new species isolated from the environment, animals and human clinical samples', *Int J Syst Evol Microbiol,* 51(Pt 3), pp. 1099-1107. DOI: 10.1099/00207713-51-3-1099.

Coleman-Derr, D., Desgarennes, D., Fonseca-Garcia, C., Gross, S., Clingenpeel, S., Woyke, T., North, G., Visel, A., Partida-Martinez, L. P. and Tringe, S. G. (2016) 'Plant compartment and biogeography affect microbiome composition in cultivated

and native Agave species', *New Phytol,* 209(2), pp. 798-811. DOI: 10.1111/nph.13697.

Corazon-Guivin, M. A., Vallejos-Tapullima, A., Rengifo-Del Aguila, S., Rondinel-Mendoza, N. V., Hernández-Cuevas, L. V., Carvajal-Vallejos, F. M. and Carballar-Hernández, S. (2022) 'Influence of Substrate Properties on Communities of Arbuscular Mycorrhizal Fungi Isolated from Agroecosystems in Peru', *Journal of Soil Science and Plant Nutrition,* 22(4), pp. 4784-4797. DOI: 10.1007/s42729-022-00960-3.

De Baere, T., Steyaert, S., Wauters, G., Des Vos, P., Goris, J., Coenye, T., Suyama, T., Verschraegen, G. and Vaneechoutte, M. (2001) 'Classification of Ralstonia pickettii biovar 3/'thomasii' strains (Pickett 1994) and of new isolates related to nosocomial recurrent meningitis as Ralstonia mannitolytica sp. nov', *Int J Syst Evol Microbiol,* 51(Pt 2), pp. 547-558. DOI: 10.1099/00207713-51-2-547.

Desiro, A., Faccio, A., Kaech, A., Bidartondo, M. I. and Bonfante, P. (2015) 'Endogone, one of the oldest plant-associated fungi, host unique Mollicutes-related endobacteria', *New Phytol,* 205(4), pp. 1464-1472. DOI: 10.1111/nph.13136.

Desiro, A., Salvioli, A., Ngonkeu, E. L., Mondo, S. J., Epis, S., Faccio, A., Kaech, A., Pawlowska, T. E. and Bonfante, P. (2014) 'Detection of a novel intracellular microbiome hosted in arbuscular mycorrhizal fungi', *ISME J,* 8(2), pp. 257-70. DOI: 10.1038/ismej.2013.151.

Dirks, A. C. and Jackson, R. D. (2020) 'Community Structure of Arbuscular Mycorrhizal Fungi in Soils of Switchgrass Harvested for Bioenergy', *Appl Environ Microbiol,* 86(19). DOI: 10.1128/AEM.00880-20.

Dolatabadi, S., Walther, G., Gerrits van den Ende, A. H. G. and de Hoog, G. S. (2014) 'Diversity and delimitation of Rhizopus microsporus', *Fungal Diversity,* 64(1), pp. 145-163. DOI: 10.1007/s13225-013-0229-6.

Fonseca-Garcia, C., Coleman-Derr, D., Garrido, E., Visel, A., Tringe, S. G. and Partida-Martinez, L. P. (2016) 'The Cacti Microbiome: Interplay between Habitat-Filtering and Host-Specificity', *Front Microbiol,* 7, pp. 150. DOI: 10.3389/fmicb.2016.00150.

Gao, C., Montoya, L., Xu, L., Madera, M., Hollingsworth, J., Purdom, E., Hutmacher, R. B., Dahlberg, J. A., Coleman-Derr, D., Lemaux, P. G. and Taylor, J. W. (2019) 'Strong succession in arbuscular mycorrhizal fungal communities', *ISME J,* 13(1), pp. 214-226. DOI: 10.1038/s41396-018-0264-0.

Glass, M. B., Steigerwalt, A. G., Jordan, J. G., Wilkins, P. P. and Gee, J. E. (2006) 'Burkholderia oklahomensis sp. nov., a Burkholderia pseudomallei-like species formerly known as the Oklahoma strain of Pseudomonas pseudomallei', *Int J Syst Evol Microbiol,* 56(Pt 9), pp. 2171-2176. DOI: 10.1099/ijs.0.63991-0.

Jansa, J., Mozafar, A., Anken, T., Ruh, R., Sanders, I. R. and Frossard, E. (2002) 'Diversity and structure of AMF communities as affected by tillage in a temperate soil', *Mycorrhiza,* 12(5), pp. 225-34. DOI: 10.1007/s00572-002-0163-z.

Kikuchi, Y., Hosokawa, T. and Fukatsu, T. (2011) 'An ancient but promiscuous host-symbiont association between Burkholderia gut symbionts and their heteropteran hosts', *ISME J,* 5(3), pp. 446-60. DOI: 10.1038/ismej.2010.150.

Kruger, M., Kruger, C., Walker, C., Stockinger, H. and Schussler, A. (2012) 'Phylogenetic reference data for systematics and phylotaxonomy of arbuscular mycorrhizal fungi from phylum to species level', *New Phytol,* 193(4), pp. 970-984. DOI: 10.1111/j.1469-8137.2011.03962.x.

Lackner, G., Mobius, N., Scherlach, K., Partida-Martinez, L. P., Winkler, R., Schmitt, I. and Hertweck, C. (2009) 'Global distribution and evolution of a toxinogenic Burkholderia-Rhizopus symbiosis', *Appl Environ Microbiol,* 75(9), pp. 2982-6. DOI: 10.1128/AEM.01765-08.

Li, C. H., Cervantes, M., Springer, D. J., Boekhout, T., Ruiz-Vazquez, R. M., Torres-Martinez, S. R., Heitman, J. and Lee, S. C. (2011) 'Sporangiospore size dimorphism is linked to virulence of Mucor circinelloides', *PLoS Pathog,* 7(6), pp. e1002086. DOI: 10.1371/journal.ppat.1002086.

Naumann, M., Schussler, A. and Bonfante, P. (2010) 'The obligate endobacteria of arbuscular mycorrhizal fungi are ancient heritable components related to the Mollicutes', *ISME J,* 4(7), pp. 862-71. DOI: 10.1038/ismej.2010.21

Ohshima, S., Sato, Y., Fujimura, R., Takashima, Y., Hamada, M., Nishizawa, T., Narisawa, K. and Ohta, H. (2016) 'Mycoavidus cysteinexigens gen. nov., sp. nov., an endohyphal bacterium isolated from a soil isolate of the fungus Mortierella elongata', *Int J Syst Evol Microbiol,* 66(5), pp. 2052-2057. DOI: 10.1099/ijsem.0.000990.

Okrasinska, A., Bokus, A., Duk, K., Gesiorska, A., Sokolowska, B., Milobedzka, A., Wrzosek, M. and Pawlowska, J. (2021) 'New Endohyphal Relationships between Mucoromycota and Burkholderiaceae Representatives', *Appl Environ Microbiol,* 87(7). DOI: 10.1128/AEM.02707-20.

Partida-Martinez, L. P. and Hertweck, C. (2005) 'Pathogenic fungus harbours endosymbiotic bacteria for toxin production', *Nature,* 437(7060), pp. 884-8. DOI: 10.1038/nature03997.

Partida-Martinez, L. P., Monajembashi, S., Greulich, K. O. and Hertweck, C. (2007) 'Endosymbiont-dependent host reproduction maintains bacterial-fungal mutualism', *Curr Biol,* 17(9), pp. 773-7. DOI: 10.1016/j.cub.2007.03.039.

Redecker, D., Raab, P., Oehl, F., Camacho, F. J. and Courtecuisse, R. (2007) 'A novel clade of sporocarp-forming species of glomeromycotan fungi in the Diversisporales lineage', *Mycological Progress,* 6(1), pp. 35-44. DOI: 10.1007/s11557-007-0524-2.

Sangabriel-Conde, W., Maldonado-Mendoza, I. E., Mancera-López, M. E., Cordero-Ramírez, J. D., Trejo-Aguilar, D. and Negrete-Yankelevich, S. (2015) 'Glomeromycota associated with Mexican native maize landraces in Los Tuxtlas, Mexico', *Applied Soil Ecology,* 87, pp. 63-71. DOI: https://doi.org/10.1016/j.apsoil.2014.10.017.

Savary, R., Masclaux, F. G. and Sanders, I. R. (2021) 'The model arbuscular mycorrhizal fungus Rhizophagus irregularis harbours endosymbiotic bacteria with a highly reduce genome', *bioRxiv*, pp. 2021.09.13.460061. DOI: 10.1101/2021.09.13.460061.

Schussler, A. and Walker, C. (2019) 'Archaeospora ecuadoriana sp. nov. from a mountainous biodiversity hotspot area in Ecuador, and transfer of Palaeospora spainiae to Archaeospora, as A. spainiae comb. nov', *Mycorrhiza,* 29(5), pp. 435-443. DOI: 10.1007/s00572-019-00913-2.

Schwarz, P., Bretagne, S., Gantier, J. C., Garcia-Hermoso, D., Lortholary, O., Dromer, F. and Dannaoui, E. (2006) 'Molecular identification of zygomycetes from culture and

experimentally infected tissues', *J Clin Microbiol,* 44(2), pp. 340-9. DOI: 10.1128/JCM.44.2.340-349.2006.

Schwarzott, D., Walker, C. and Schussler, A. (2001) 'Glomus, the largest genus of the arbuscular mycorrhizal fungi (Glomales), is nonmonophyletic', *Mol Phylogenet Evol,* 21(2), pp. 190-7. DOI: 10.1006/mpev.2001.1007.

Stockinger, H., Walker, C. and Schussler, A. (2009) ''Glomus intraradices DAOM197198', a model fungus in arbuscular mycorrhiza research, is not Glomus intraradices', *New Phytol,* 183(4), pp. 1176-1187. DOI: 10.1111/j.1469-8137.2009.02874.x.

Takashima, Y., Seto, K., Degawa, Y., Guo, Y., Nishizawa, T., Ohta, H. and Narisawa, K. (2018) 'Prevalence and Intra-Family Phylogenetic Divergence of Burkholderiaceae-Related Endobacteria Associated with Species of Mortierella', *Microbes Environ,* 33(4), pp. 417-427. DOI: 10.1264/jsme2.ME18081.

Toomer, K. H., Chen, X., Naito, M., Mondo, S. J., den Bakker, H. C., VanKuren, N. W., Lekberg, Y., Morton, J. B. and Pawlowska, T. E. (2015) 'Molecular evolution patterns reveal life history features of mycoplasma-related endobacteria associated with arbuscular mycorrhizal fungi', *Mol Ecol,* 24(13), pp. 3485-500. DOI: 10.1111/mec.13250.
